# *N*-Alkylated Iminosugar Based Ligands: Synthesis and Inhibition of Human Lysosomal β-Glucocerebrosidase

**DOI:** 10.3390/molecules25204618

**Published:** 2020-10-11

**Authors:** Andreas Wolfsgruber, Martin Thonhofer, Patrick Weber, Seyed A. Nasseri, Roland Fischer, Michael Schalli, Arnold E. Stütz, Stephen G. Withers, Tanja M. Wrodnigg

**Affiliations:** 1 Glycogroup, Institute of Chemistry and Technology of Biobased Systems, Graz University of Technology, Stremayrgasse 9, A-8010 Graz, Austria; andreas.wolfsgruber@tugraz.at (A.W.); thonhofer@tugraz.at (M.T.); patrick.weber@tugraz.at (P.W.); michael.schalli@medunigraz.at (M.S.); stuetz@tugraz.at (A.E.S.); 2 Chemistry Department, University of British Columbia, 2036 Main Mall, Vancouver, BC V6T 1Z1, Canada; snasseri@chem.ubc.ca (S.A.N.); withers@chem.ubc.ca (S.G.W.); 3 Institute of Inorganic Chemistry, Graz University of Technology, Stremayrgasse 9, A-8010 Graz, Austria; roland.fischer@tugraz.at

**Keywords:** *N*-alkylated iminosugars, inhibitors for β-glucocerebrosidase, tools for glycoprocessing enzymes, β-glucosidase inhibitors, glycomimetics

## Abstract

The scope of a series of *N*-alkylated iminosugar based inhibitors in the d-*gluco* as well as d-*xylo* configuration towards their interaction with human lysosomal β-glucocerebrosidase has been evaluated. A versatile synthetic toolbox has been developed for the synthesis of *N*-alkylated iminosugar scaffolds conjugated to a variety of terminal groups via a benzoic acid ester linker. The terminal groups such as nitrile, azide, alkyne, nonafluoro-*tert*-butyl and amino substituents enable follow-up chemistry as well as visualisation experiments. All compounds showed promising inhibitory properties as well as selectivities for β-glucosidases, some exhibiting activities in the low nanomolar range for β-glucocerebrosidase.

## 1. Introduction

**Figure 1:**
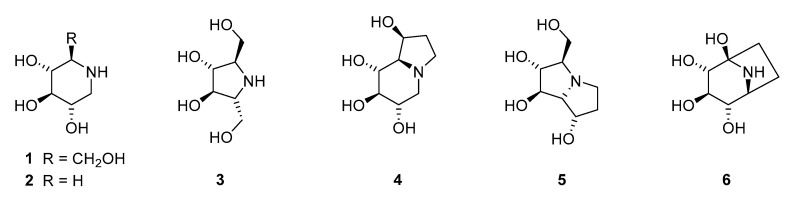
Paradigmatic structural examples of selected iminosugars **1**–**6**.

Iminoalditols, also called iminosugars, represent polyhydroxylated alkaloids and are structurally related to common carbohydrates in which the endocyclic oxygen atom is replaced by a basic trivalent nitrogen atom. This exchange endows this class of glycomimetics with basic properties that are responsible for their remarkable biological activity. Iminoalditols are widely distributed in nature and can be found in bacteria, fungi or plants [1]. Common structural features are monocyclic systems such as piperidines (e.g., compounds **1** and **2**) as well as pyrrolidines (e.g., **3**) and bicyclic scaffolds, for example indolizidines (e.g., **4**), pyrrolizidines (e.g., **5**), and *nor*tropanes (e.g., **6**) (see Figure 1).

The wide variety of iminosugars known today is also the result of synthetic efforts to replace the ring oxygen with heteroatoms such as nitrogen, sulfur or phosphorus in the early 1960s. In 1966, the first synthetically achieved iminosugar 1,5-dideoxy-1,5-imino-d-glucitol (**1**), better known as 1-deoxynojirimycin (1-DNJ), was reported [2,3] and with this structure, an entire interdisciplinary scientific research field was born [4,5,6].

In general, representatives of this substance class are known as ligands for carbohydrate-processing enzymes (CPEs), as they interact with the active site of the respective protein as mimics of the natural substrate [7,8,9,10]. Iminosugar-based structures are versatile probes and have been used in several different applications as inhibitors, [11,12] therapeutics [8,9,10] and probes for activity-based protein profiling [13,14]. In particular, these structures have proved interesting for the therapy of CPE- associated diseases such as cancer [15], bacterial infections [16], HIV [17], HPV [18], influenza [19], hepatitis [20], the dengue virus [21], malaria [22] and fungal infections [23]. In addition, iminosugar based glycomimetics have shown encouraging results as so-called pharmacological chaperones (PCs) in the treatment of lysosomal storage diseases (LSDs) [24,25,26]. Due to their strong interaction with the active site of their respective enzyme, they can stabilize the correctly folded structures of mutant lysosomal enzymes, thereby obviating their cellular clearance [24,27].

An interesting enzyme in this respect is human lysosomal β-glucocerebrosidase (glucosylceramidase, GCase, EC 3.2.1.45, glycosidase family 30 [28]). This enzyme catalyses the degradation of the β-glycosidic linkage of glucosylceramide or glucosylsphingosine into d-glucose units and ceramide or sphingosine, respectively, which is the last step of the lysosomal catabolism of glycosylsphingosines in the autophagy-lysosomal pathway. A malfunction of GCase causes Gaucher disease (GD), a severe lysosomal storage disorder with an incidence of approx. 1 in every 50,000 people worldwide [29]. Additionally, Parkinson’s as well as Alzheimer’s disease have been linked to GCase deficiency [30,31].

**Figure 2:**
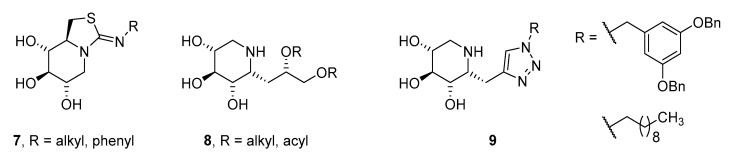
Selected structures of iminosugar based glycomimetics.

In this context, a large variety of iminosugar based glycomimetics have been investigated [4,32,33,34,35,36,37]. Paradigmatic examples are shown in Figure 2. Compounds **7** [38] have been introduced by Mellet and coworkers, d-*xylo* configured 1-*C*-alkyl iminosugars **8** [39] represent another important family which is also true for compounds **9** [40], all showing significant properties as pharmacological chaperones for mutant GCases.

**Figure 3:**
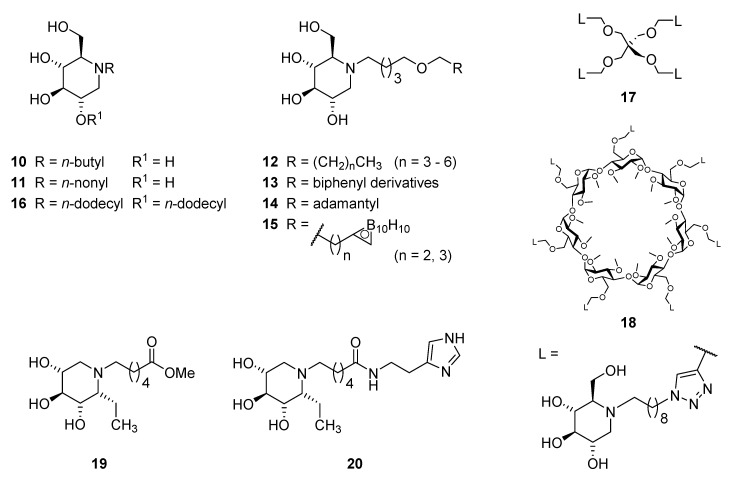
Selected structures of *N*-alkylated iminosugars.

We are particularly interested in iminoalditol structures, where the modification is located at the ring nitrogen in order to install customised features for different research related properties of GCase. In this context, well-known *N*-alkylated examples are compounds **10** and **11**, which inhibit GCase with *K*_i_ values of 116 µM and 0.3 µM, respectively [41] (Figure 3).A large variety of *N*-substituted iminosugar based glycomimetics was contributed by Overkleeft and co-workers, their compound collection of 1-DNJ derivatives contain various hydrophobic groups at the ring nitrogen such as alkyl chains **12** [42], different biphenyl derivatives **13** [43], an adamantly group **14** [44] and carborane-modified moieties **15** [45], just to mention a few. All of these compounds show *K*_i_-values in the low micro to nanomolar range against GCase (respective *K*_i_ values see references [42,43,44,45]). Additionally, *N*,*O*-dialkylated derivatives of 1-DNJ (e.g., compound **16**) were reported recently as potent ligands of ceramide transport protein [46]. Furthermore, multivalent iminosugar-based structures [47] (e.g., compounds **17** and **18**) were reported to exhibit multivalency effects on GCase inhibition with *K*_i_ values of 55 nM and 285 nM, respectively [48]. 1,5-Dideoxy-1,5-imino-d-xylitol-based *N*-alkylated derivatives **19** and **20** were found as selective inhibitors of GCase with *K*_i_ values of 57 µM and 4.1 µM, respectively. [36].

Now, we present a robust synthetic method for the simple and concise composition of diversity driven *N*-alkylated iminosugar based inhibitors, which have the potential to be used as powerful tools such as inhibitors, pharmacological chaperones or probes for investigations of glycoprocessing enzymes in general and GCase in particular.

## 2. Results

### 2.1. Synthesis

**Figure 4:**
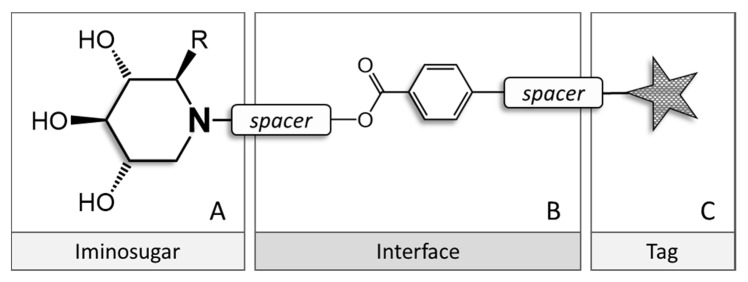
Building block concept for iminosugar-based ligands (R = CH_2_OH or H).

The developed synthetic concept relies on the coupling of three main building blocks, as shown in Figure 4: The iminosugar scaffold (**A**) acts as an active site ligand; the interface moiety (**B**) enables variation in length and consequently properties of the final product; the terminal building block (tag) (**C**) provides various functional groups that can be customised for further applications.

Since we are interested in inhibiting β-glucosidases, we employed d-*gluco* as well as d-*xylo* configured iminosugar scaffolds. The component constructed from the interface (**B**) and terminal building block or tag (**C**), including a six-carbon alkyl spacer for constant conjugation to the iminosugar (**A**), was intended to react either through an *N*-alkylation reaction or via reductive amination with the ring nitrogen of the respective iminosugar moiety. Therefore, various esters of modified benzoic acids and different ω-halogen alcohols (6-bromo-, and 6-chlorohexanol) act as suitable building blocks. The alkyl halide employed can either react directly in *N*-alkylation reactions, or can alternatively be converted into the corresponding aldehyde functionality through a Kornblum oxidation, as required for reductive amination reactions. As terminal tags we have chosen nitrile, azide, alkyne, nonafluoro-*tert*-butyl and *N*-dansyl functionalities which will enable follow-up chemistry as well as visualisation experiments in further investigations.

**Scheme 1:**
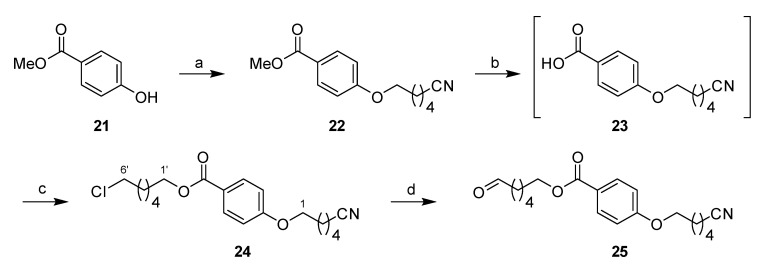
Reagents and Conditions: (a) 6-Bromohexanenitrile, K_2_CO_3_, acetone, 86%; (b) NaOH, dioxane/H_2_O; (c) 6-chlorohexanol, PPh_3_, DIAD, THF, 91% over 2 steps; (d) NaHCO_3_, DMSO, 120 °C, 71%.

To keep the synthetic approach as flexible as possible, the conceptual synthetic strategy has been designed to start with the central hydroxybenzoic acid motif, to which the two handles are introduced. The installation of the terminal nitrile via an ether bond can be realised by a simple Williamson ether synthesis employing 6-bromohexanenitrile and potassium carbonate with methyl hydroxybenzoate (**21**) to obtain compound **22** (Scheme 1). Saponification of the methyl ester leads to the previously reported benzoic acid derivative **23** [49]. Subsequent esterification under standard Mitsunobu conditions with 6-chlorohexanol, PPh_3_ and DIAD in THF provided compound **24**. In light of the poor reactivity of alkyl chlorides in *N*-alkylation reactions, a second parallel series of building blocks containing a more reactive aldehyde functionality was prepared via a Kornblum reaction [50,51]. This would allow coupling to the amine via a reductive amination reaction in the event that the alkyl halide route was unsuccessful. Therefore, compound **24** was treated with sodium hydrogen carbonate and DMSO at 120 °C to give aldehyde building block **25**. The nitrile can be used in orthogonal follow up chemistry such as (2+3) cycloaddition [52,53] for further labelling of the enzyme-ligand complex.

**Scheme 2:**
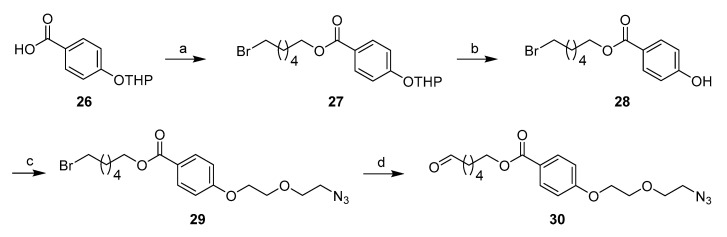
Reagents and Conditions: (a) 6-Bromohexanol, PPh_3_, DIAD, THF, 85%; (b) Amberlite^®^ IR-120H^+^, dioxane/H_2_O, 45 °C, 76%; (c) N_3_(CH_2_CH_2_O)_2_H, PPh_3_, DIAD, THF, 82%; (d) NaHCO_3_, DMSO, 120 °C, 86%.

Additionally, the reaction sequence can be rearranged in order to start with the installation of the ester bond in the first step followed by formation of the ether (cf. Scheme 1 and Scheme 2). To effect this the hydroxyl group of hydroxybenzoic acid was protected with a THP group under standard reaction conditions, giving compound **26** [54] which was then converted into benzoic ester **27** using 6-bromohexanol, PPh_3_ and DIAD in THF. Removal of the THP group under acidic conditions provided known compound **28** [55]. Subsequent etherification under standard Mitsunobu conditions with 2-(2-azidoethoxy)ethanol [56] gave intermediate **29**. Finally, the bromine functionality was transformed into an aldehyde by a Kornblum oxidation to give compound **30**. In order to evaluate different types of spacers, and to demonstrate the diversity of the presented synthetic tool box, a 2-(2-hydroxyethoxy)ethanol chain has been introduced in this interface (compound **30**). The azide group allows for subsequent click chemistry tagging of the ligand-enzyme complex after incubation for labelling or quantification experiments [57].

**Scheme 3:**
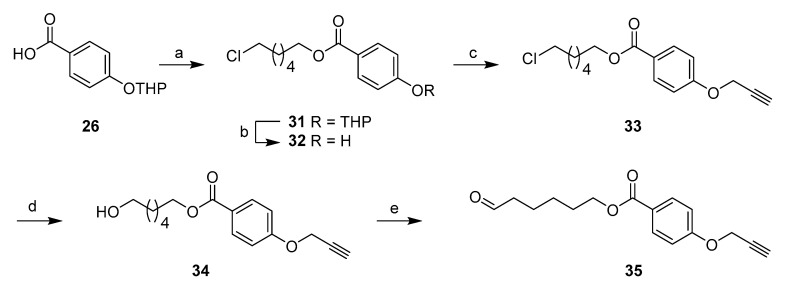
Reagents and Conditions: (a) 6-Chlorohexanol, PPh_3_, DIAD, THF, 51%; (b) Amberlite^®^ IR-120H^+^, dioxane/H_2_O, 40 °C, 96%; (c) 3-bromoprop-1-yne, K_2_CO_3_, acetone, 82%; (d) NaHCO_3_, DMSO/H_2_O, 100 °C, 77%; (e) DMP, CH_2_Cl_2_, 69%.

The same approach has been followed for the introduction of a terminal alkyne group to enable orthogonal follow up click chemistry (Scheme 3). Therefore, 6-chlorohexanol has been employed in a Mitsunobu reaction with THP protected hydroxybenzoic acid **26** to yield ester **31**. Removal of the THP group under acidic conditions using ion exchange resin liberated alcohol **32**. Subsequent etherification with 3-bromoprop-1-yne and potassium carbonate in acetone led to structure **33**. To demonstrate an additional procedure for the preparation of the desired aldehyde, the alkyl chloride was first hydrolysed to alcohol **34** using modified Kornblum reaction conditions. Therefore, compound **33** was treated with NaHCO_3_ in a mixture of DMSO and water at 100 °C to yield alcohol **34** which, in a second step, was oxidized with Dess Martin’s reagent (DMP) to desired component **35**. X-ray diffraction (XRD) studies unambiguously confirmed the structure of **35** (CCDC 2021385, see SM Figure S1).

**Scheme 4:**
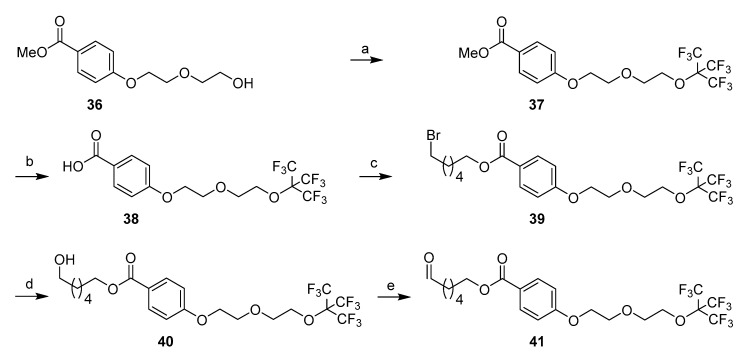
Reagents and Conditions: (a) (CF_3_)_3_COH, PPh_3_, DIAD, THF, 77%; (b) NaOH, dioxane/H_2_O; (c) 6-bromohexanol, PPh_3_, DIAD, THF; (d) NaHCO_3_, DMSO/H_2_O, 100 °C, 49% over 3 steps; (e) DMP, CH_2_Cl_2_, 84%.

Next, we wanted to introduce a reporter group to allow subsequent mass spectrometric monitoring of reactions by introducing a heavy substituent, in particular a nonafluoro-*tert*-butoxy group (Scheme 4) [58]. Therefore, diethylenoxy benzoic acid methyl ester **36** was synthesised as described previously from methyl hydroxybenzoate (**21**) and 2-(2-chloroethoxy)ethanol [59]. Compound **36** underwent a Mitsunobu reaction employing nonafluoro-*tert*-butanol, PPh_3_ and DIAD in THF to give compound **37**. Follow-up chemistry for the introduction of the aldehyde at the other terminus of the hydroxybenzoic ester moiety was performed as described before. Saponification of the methyl ester provided benzoic acid derivative **38**, followed by esterification under Mitsunobu conditions using 6-bromohexanol to obtain alkyl bromide **39**. As seen in the 2 step conversion of the halocarbon into the corresponding aldehyde (compare Scheme 3), the bromine (compound **39**) could be successfully hydrolyzed to alcohol **40** then oxidized to aldehyde **41** using Dess Martin´s reagent. Alcohol **40** can be formed starting from either the bromide or chloride of type **39**.

**Scheme 5:**
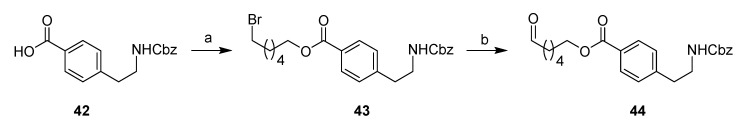
Reagents and Conditions: (a) 6-Bromohexanol, PPh_3_, DIAD, THF, 68%; (b) NaHCO_3_, DMSO, 120 °C, 49%.

In order to gain access to an amino substituent at the terminal end ready for further functionalisations with for example fluorescent dyes, benzoic acid moiety **42** [60] carrying a Cbz-protected aminoethyl spacer-arm in its para position was introduced (Scheme 5). Esterification of compound **42** to compound **43** followed by oxidation, as before, yielded aldehyde **44**.

**Scheme 6:**
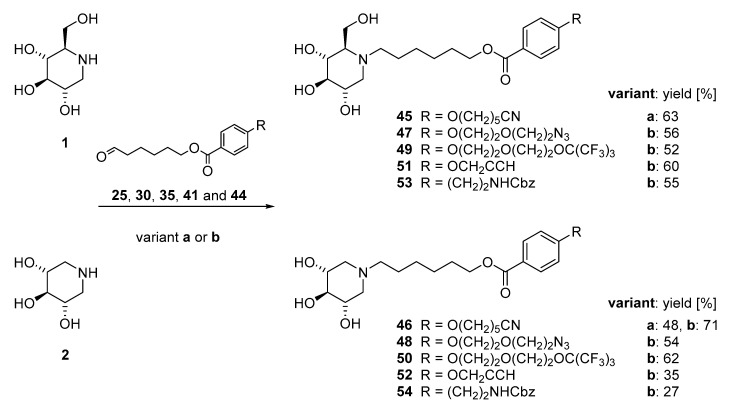
Reagents and Conditions: Overview of various reductive amination reactions and respective conditions: (a) Pd/C (10%), H_2_-atm., AcOH cat., MeOH; (b) NaBH_3_CN, AcOH cat., MeOH.

Assembly of the different building blocks via halides **24**, **29**, **33**, **39** and **43**, turned out to be problematic since the halides were rather unreactive, requiring elevated temperatures and extended reaction times, hence preparatively unsatisfying yields below 10% were obtained. Therefore, a reductive amination reaction between the aldehydes of the respective components **25**, **30**, **35**, **41** and **44** and the ring nitrogen of the iminosugars 1-DNJ (**1**) and 1,5-dideoxy-1,5-imino-d-xylitol (DIX, **2**) was employed since this method allowed smoother and faster reactions leading to far better yields compared to the *N*-alkylation approach (Scheme 6). The two chosen iminosugars, **1** [61] and **2** [62] were synthesised as previously described by our group.

Two different reducing methods were employed for the reductive amination reaction between the aldehyde building block and the iminosugar scaffold, depending on the nature of the functional group on the phenyl linker. Reaction of the cyano group-containing building block **25** with iminosugars **1** and **2** was carried out under an atmosphere of H_2_ with Pd/C (10%) as catalyst (variant **a**, Scheme 6), yielding the corresponding d-*gluco* and d-*xylo* configured structures **45** (63%) and **46** (48%), respectively.

All other reductive aminations were performed with NaBH_3_CN in methanol (variant **b**, Scheme 6) to avoid reduction of other substituents present in the respective molecules. In this way compounds **47** and **48** were obtained in yields of 56% and 54%, respectively. Likewise the alkyne-containing building block **35** and NHCbz-carrying component **44** reacted with iminosugars **1** and **2** to give compounds **51** (60%), **52** (35%), **53** (55%) and **54** (27%) in the yields shown using NaBH_3_CN. Indeed the same method also worked well with nonafluoro-*tert*-butyl group reagent **41** yielding iminosugar derivatives **49** and **50** in yields of 52% and 62% respectively while the yield of compound **46** was increased up to 71% employing this methodology, compared to 48% with Pd/C and H_2_.

**Scheme 7:**
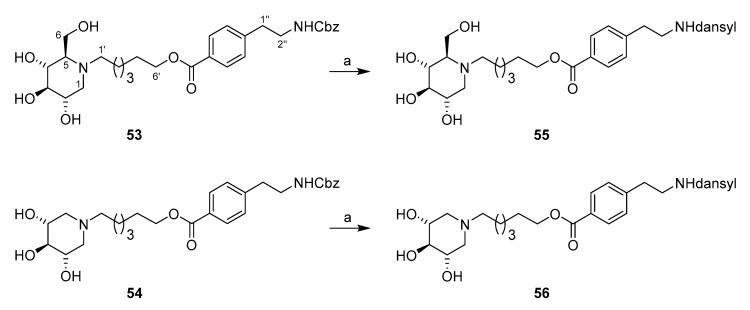
Reagents and Conditions: (a) Pd (10%), H_2_-atm., MeOH, AcOH then dansyl chloride, Et_3_N, MeOH (50% for **55**; 45% for **56**, over two steps).

*N*-Cbz-Deprotection of compounds **53** and **54** was achieved using Pd/C (10%) under an atmosphere of H_2,_ allowing reaction of the amines with dansyl chloride in the presence of triethylamine in MeOH to give the desired dansylated inhibitors of both d-*gluco* and d-*xylo* configuration, compounds **55** and **56**, respectively (Scheme 7).

### 2.2. Biological Evaluation

Inhibition constants were determined for the interaction of each of a selected set of glycoside hydrolases (GHs) with the *N*-alkylated iminosugar based glycomimetics **45**–**56** (Table 1). With the exception of the terminal alkyne **51**, all compounds were better inhibitors of the human lysosomal β-glucosidase GCase (GH30) than any of the other enzymes, including the bacterial β-glucosidase from *Agrobacterium* species. (Abg, GH1). In all cases, the d-*gluco* configured iminosugar based compounds showed better activities against both β-glucosidases GCase and Abg compared to the d-*xylo* analogs. None of the compounds were particularly useful inhibitors of either the human lysosomal α-galactosidase (Fabrazyme, GH27) or the β-galactosidase from *Escherichia. coli* (*E. coli*, GH2). This is not surprising since these enzymes are fairly specific galactosidases. However, all compounds turned out to inhibit the GH35 bovine liver β-galactosidase in the micromolar range. The presence of aromatic substituents caused some unexpected inhibition, most notably with α-glucosidase from *Saccharomyces cerevisiae* (*S.cer*., GH13) and β-galactosidase from *E. coli* likely due to adventitious interactions. This additional binding interaction is also seen with the “productive” inhibitor/enzyme combinations, with the 1-DNJ derivatives **53** (NHCbz) and **55** (NHdansyl), increasing affinity for GCase down to *K*_i_ values of 22 and 18 nM, respectively. This follows a trend we have observed with most of our compounds, that the dansyl moiety contributes significantly to a better interaction with β-glucosidases [37,63]. Interestingly, β-glucosidase from Abg shows a slight preference for a shorter handle between the interface and the terminal building block as in compounds **51**–**56** with *K*_i_ values in the nanomolar range (Table 1) compared to compounds **45**–**50** wherein the handle contains a six membered chain and has *K*_i_ values in the low micromolar range. This trend cannot be detected with GCase.

## 3. Materials and Methods

### 3.1. General Methods

NMR Spectra were recorded on an INOVA 500 spectrometer (Varian, Palo Alto, CA, USA) operating at 499.82 MHz (^1^H), and at 470.3 MHz (^19^F) or on a Bruker (Billerica, MA, USA) Ultrashield spectrometer at 300.36 (^1^H) and 75.53 (^13^C) MHz, respectively. CDCl_3_ was employed for aromatic compounds and CD_3_OD for unprotected ligands as indicated. Chemical shifts are listed in ppm employing residual, non-deuterated solvent as the internal standard. Structures of crucial intermediates were unambiguously assigned APT, COSY and HSQC experiments. Carbon and hydrogen numbering in NMR spectra was conducted as indicated in representative structures shown above (Scheme 1, Scheme 7). Optical rotations were measured on a Perkin Elmer 341 polarimeter (Perkin Elmer, Waltham, MA, USA) at the wavelength of 589 nm and a path length of 10 cm at 20 °C. MALDI-TOF Mass Spectrometry was performed on a Micromass TofSpec 2E Time-of-Flight Mass Spectrometer (Waters Corporation, Milford, MA, USA). Analytical TLC was performed on precoated aluminum plates Silica Gel 60 F254 (E. Merck 5554, E. Merck, Darmstadt, Germany), detected with UV light (254 nm). For staining, a solution of vanillin (9 g) in a mixture of H_2_O (950 mL)/EtOH (750 mL)/H_2_SO_4_ (120 mL) or ceric ammonium molybdate (100 g ammonium molybdate/8 g ceric sulphate in 1 L 10% H_2_SO_4_) were employed followed by heating on a hotplate. For column chromatography, silica gel 60 (230–400 mesh, E. Merck 9385) or silica gel 60 (Acros Organics, AC 24036, Thermo Fisher Scientific Inc., Waltham, MA, USA) were used. Unless otherwise specified, all starting materials, reagents and solvents are commercially available and were used without further purification. Reactions were performed at ambient temperature and ambient pressure. Otherwise, conditions are explicitly specified. Reaction monitoring was performed by TLC. NMR spectra for new compounds and XRD data for structure **35** are presented in the Supplementary Materials.

### 3.2. General Synthetic Procedures

#### 3.2.1. General Procedure A: (Mitsunobu Reaction)

A 10% solution of the respective starting material (1.0 equiv.) in THF, Ph_3_P (1.0 equiv.), diisopropyl azodicarboxylate (DIAD, 1.0 equiv.) and the respective alcohol (1.0 equiv.) was stirred until completed conversion of the reactants was detected. Subsequently, the reaction mixture was diluted with CH_2_Cl_2_ and washed consecutively with aqueous HCl (2 *N*) and saturated NaHCO_3_. After drying over Na_2_SO_4_, the filtrate was concentrated under reduced pressure to provide the corresponding crude product.

#### 3.2.2. General Procedure B: (Kornblum Oxidation)

Variant 1: (Conversion of a halocarbon to the corresponding aldehyde)

A 10% solution of the respective halocarbon (1.0 equiv.) in DMSO was stirred with NaHCO_3_ (4.0-6.0 equiv.) at 120 °C until completed conversion of the starting material was detected. After allowing the system to cool to room temperature, the reaction mixture was diluted with CH_2_Cl_2_ and subsequently washed with water. The combined organic layers were dried over Na_2_SO_4_, filtered and concentrated under reduced pressure providing the corresponding crude product.

Variant 2: (Conversion of a halocarbon to the corresponding alcohol)

Alternatively, the respective halocarbon was dissolved in a mixture of DMSO and water (10:1 *v*/*v*) instead of pure DMSO. The remaining protocol is identical to variant 1.

#### 3.2.3. General Procedure C: (Dess-Martin Oxidation)

To a 10% solution of the respective alcohol (1 equiv.) in CH_2_Cl_2_, Dess-Martin periodinane (1.1 equiv.) was added. After completed conversion of the starting material, the reaction mixture was carefully quenched with saturated NaHCO_3_. After separation and drying over Na_2_SO_4_, the organic layers were filtered off and concentrated under reduced pressure to obtain the corresponding crude aldehyde.

#### 3.2.4. General Procedure D: (Reductive Amination employing NaBH_3_CN)

A 20% solution of the respective aldehyde (1.0 equiv.) and iminosugar (1.0–1.2 equiv.) in MeOH (containing a catalytic amount of AcOH) was stirred for 15 min before NaBH_3_CN (1.5–3.0 equiv.) was added. After completed conversion of the starting materials was detected, the reaction mixture was concentrated under reduced pressure to provide the corresponding crude title compound.

*Methyl 4-((5-cyanopentyl)oxy)benzoate* (**22**). To a stirred solution of commercially available methyl 4-hydroxybenzoate (**21**, 2.01 g, 13.2 mmol) in acetone (30 mL), K_2_CO_3_ (4.40 g, 31.8 mmol) and 6-bromohexanenitrile (2.61 mL, 19.7 mmol) were added. After complete conversion of the starting material, the reaction mixture was filtered off, diluted with CH_2_Cl_2_ and washed three times with H_2_O. The combined organic layers were dried over Na_2_SO_4_, filtered off and the solvent was removed under reduced pressure. Purification on silica gel (cyclohexane-ethyl acetate 12:1) provided compound **22** (2.86 g, 11.6 mmol, 87.5%) as a colourless syrup. ^1^H-NMR (300 MHz, CDCl_3_) δ = 7.97 (d, 2 H, Ar), 6.89 (d, 2 H, Ar), 4.02 (t, 2 H, H-1), 3.87 (s, 3 H, OCH_3_), 2.38 (t, 2 H, H-5), 1.88–1.59 (po, 6 H, H-2, H-3, H-4); ^13^C-NMR (75.5 MHz, CDCl_3_) δ = 166.9 (OC = O), 162.7 (ipso), 131.7 (Ar), 122.7 (ipso), 119.6 (CN), 114.1 (Ar), 67.6 (C-1), 51.9 (OCH_3_), 28.5 (C-2), 25.5, 25.3 (C-3, C-4), 17.2 (C-5); MS: Calcd for [C_14_H_17_NO_3_Na]: *m/z* 270.1106 [M + Na]^+^; Found [M + Na]^+^ 270.1106.

*6-Chlorohexyl 4-((5-cyanopentyl)oxy)benzoate* (**24**). A solution of **22** (509 mg, 2.06 mmol) in dioxane/H_2_O (3:1 *v*/*v*, 40 mL) was treated with solid NaOH (329 mg, 8.23 mmol) and stirred for 3 h. After completed conversion of the starting material, the reaction mixture was neutralised by addition of acidic ion exchange resin (Amberlite^®^ IR-120H^+^ washed with H_2_O), filtrated and evaporated to dryness. The resulting intermediate **23** [49] was immediately used in the next step without further purification. Following general procedure A, **23** was treated with 6-chlorohexanol (275 µL, 2.06 mmol), PPh_3_ (540 mg, 2.06 mmol) and DIAD (404 µL, 2.06 mmol). Purification on silica gel (cyclohexane-ethyl acetate 10:1) afforded title compound **24** (659 mg, 1.87 mmol, 90.8% over two steps) as a colourless wax. ^1^H-NMR (300 MHz, CDCl_3_) δ = 7.94 (d, 2 H, Ar), 6.86 (d, 2 H, Ar), 4.24 (t, 2 H, H-1´), 3.98 (t, 2 H, H-1), 3.52 (t, 2 H, H-6´), 2.35 (t, 2 H, H-5), 1.83-1.35 (po, 14 H, H-2´, H-3´, H-4´, H-5´, H-2, H-3, H-4); ^13^C-APT NMR (75.5 MHz, CDCl_3_) δ = 166.3 (OC = O), 162.6 (ipso), 131.5 (Ar), 122.8 (ipso), 119.5 (CN), 114.0 (Ar), 67.5 (C-1), 64.5 (C-1´), 44.9 (C-6´), 32.4, 28.6, 28.3, 26.5, 25.4, 25.3, 25.1 (C-2´, C-3´, C-4´, C-5´, C-2, C-3, C-4), 17.0 (C-5); MS: Calcd for [C_19_H_26_ClNO_3_Na]: *m/z* 374.1499 [M + Na]^+^; Found [M + Na]^+^ 374.1496.

*6-Oxohexyl-4-((5-cyanopentyl)oxy)benzoate* (**25**). Following general procedure B1, **24** (659 mg, 1.87 mmol) was reacted with NaHCO_3_ (630 mg, 7.50 mmol). Purification on silica gel (cyclohexane-ethyl acetate 5:1) afforded compound **25** (438 mg, 1.32 mmol, 70.6%) as a colourless oil. ^1^H-NMR (300 MHz, CDCl_3_) δ = 9.77 (s, 1 H, HC = O), 7.97 (d, 2 H, Ar), 6.89 (d, 2 H, Ar), 4.28 (t, 2 H, H-1´), 4.02 (t, 2 H, H-1), 2.47 (m, 2 H, H-5´), 2.39 (t, 2 H, H-5), 1.89–1.40 (po, 12 H, H-2´, H-3´, H-4´, H-2, H-3, H-4); ^13^C-NMR (75.5 MHz, CDCl_3_) δ = 202.5 (HC = O), 166.5 (OC = O), 162.8 (ipso), 131.7 (Ar), 122.9 (ipso), 119.6 (CN), 114.2 (Ar), 67.6 (C-1), 64.5 (C-1´), 43.9 (C-5´), 28.7, 28.5, 25.8, 25.5, 25.3, 21.8 (C-2´, C-3´, C-4´, C-2, C-3, C-4), 17.3 (C-5); MS: Calcd for [C_19_H_25_NO_4_Na]: *m/z* 354.1681 [M + Na]^+^; Found [M + Na]^+^ 354.1666.

*6-Bromohexyl 4-((tetrahydro-2H-pyran-2-yl)oxy)benzoate* (**27**). Following general procedure A, compound 26 [54 (982 mg, 4.42 mmol) was treated with 6-bromohexanol (578 µL, 4.42 mmol), PPh_3_ (1.16 g, 4.42 mmol) and DIAD (868 µL, 4.42 mmol). Purification on silica gel (cyclohexane-ethyl acetate 20:1) provided compound **27** (1.45 g, 3.76 mmol, 85.1%) as a colourless syrup. ^1^H-NMR (300 MHz, CDCl_3_) δ = 7.97 (d, 2 H, Ar), 7.06 (d, 2 H, Ar), 5.50 (dd, 1 H, THP), 4.28 (t, 2 H, H-1´), 3.85 (m, 1 H, THP), 3.61 (m, 1 H, THP), 3.41 (t, 2 H, H-6´), 2.06-1.37 (po, 14 H, H-2´, H-3´, H-4´, H-5´, 3× CH_2_-THP); ^13^C-NMR (75.5 MHz, CDCl_3_) δ = 166.5 (OC = O), 161.0 (ipso), 131.5 (Ar), 123.6 (ipso), 116.0 (Ar), 96.2 (THP), 64.6 (C-1´), 62.2 (THP), 33.8 (C-6´), 32.8, 30.3, 28.8, 28.0, 25.4, 25.2 (C-2´, C-3´, C-4´, C-5´, 2× THP), 18.7 (THP); MS: Calcd for [C_18_H_25_BrO_4_Na]: *m/z* 407.0834 [M + Na]^+^; Found [M + Na]^+^ 407.0840.

*6-Bromohexyl 4-hydroxybenzoate* (**28**). A solution of **27** (1.05 g, 2.73 mmol) in 40 mL dioxane/H_2_O (3:1 *v*/*v*) was treated with acidic ion exchange resin (Amberlite^®^ IR-120H^+^ washed with H_2_O) at 45 °C. After completed conversion of the starting material, the reaction mixture was filtered and the solvents were removed under reduced pressure. Purification on silica gel (cyclohexane-ethyl acetate 10:1) provided known compound **28** [55] (622 mg, 2.07 mmol, 75.8%) as a colourless wax. ^1^H-NMR (300 MHz, CDCl_3_) δ = 7.94 (d, 2 H, Ar), 7.76 (s, 1 H, OH), 6.92 (d, 2 H, Ar), 4.30 (t, 2 H, H-1´), 3.41 (t, 2 H, H-6´), 1.84-1.72 (m, 2 H, H-5´), 1.71-1.59 (m, 2 H, H-2´), 1.54-1.34 (po, 4 H, H-3´, H-4´); ^13^C-NMR (75.5 MHz, CDCl_3_) δ = 167.6 (OC = O), 161.0 (ipso), 132.0 (Ar), 122.0 (ipso), 115.5 (Ar), 65.1 (C-1´), 33.8 (C-6´), 32.7 (C-5´), 28.6 (C-2´), 27.9, 25.3 (C-3´, C-4´); MS: Calcd for [C_13_H_17_BrO_3_Na]: *m/z* 323.0259 [M + Na]^+^; Found [M + Na]^+^ 323.0258.

*6-Bromohexyl 4-(2-(2-azidoethoxy)ethoxy)benzoate* (**29**). According to general procedure A, compound **28** (290 mg, 0.962 mmol) was reacted with 2-(2-azidoethoxy)ethanol [56] (126 mg, 0.962 mmol), PPh_3_ (252 mg, 0.962 mmol) and DIAD (189 µL, 0.962 mmol). Silica gel chromatography (cyclohexane-ethyl acetate 20:1) of the resulting mixture gave structure **29** (325 mg, 0.784 mmol, 81.5%) as a colourless syrup. ^1^H-NMR (300 MHz, CDCl_3_) δ = 7.98 (d, 2 H, Ar), 6.94 (d, 2 H, Ar), 4.28 (t, 2 H, H-1´), 4.19 (m, 2 H, H-1), 3.89 (m, 2 H, H-2), 3.75 (m, 2 H, H-3), 3.41 (po, 4 H, H-6´, H-4), 1.87 (m, 2 H, H-5´), 1.78 (m, 2 H, H-2´), 1.55-1.40 (po, 4 H, H-3´, H-4´); ^13^C-NMR (75.5 MHz, CDCl_3_) δ = 166.5 (OC = O), 162.6 (ipso), 131.7 (Ar), 123.3 (ipso), 114.3 (Ar), 70.5 (C-3), 69.7 (C-2), 67.7 (C-1), 64.7 (C-1´), 50.8 (C-4), 33.8 (C-6´), 32.8 (C-5´), 28.8 (C-2´), 28.0, 25.4 (C-3´, C-4´); MS: Calcd for [C_17_H_24_BrN_3_O_4_Na]: *m/z* 436.0848 [M + Na]^+^; Found [M + Na]^+^ 436.0860.

*6-Oxohexyl 4-(2-(2-azidoethoxy)ethoxy)benzoate* (**30**). Following general procedure B1, **29** (223 mg, 0.538 mmol) was reacted with NaHCO_3_ (226 mg, 2.69 mmol). Purification on silica gel (cyclohexane-ethyl acetate 5:1) afforded compound **30** (161 mg, 0.461 mmol, 85.7%) as a pale yellow syrup. ^1^H-NMR (300 MHz, CDCl_3_) δ = 9.75 (s, 1 H, HC = O), 7.96 (d, 2 H, Ar), 6.92 (d, 2 H, Ar), 4.25 (t, 2 H, H-1´), 4.17 (m, 2 H, H-1), 3.87 (m, 2 H, H-2), 3.73 (m, 2 H, H-3), 3.40 (m, 2 H, H-4), 2.45 (m, 2 H, H-5´), 1.82-1.63 (po, 4 H, H-2´, H-4´), 1.46 (m, 2 H, H-3´); ^13^C-NMR (75.5 MHz, CDCl_3_) δ = 202.4 (HC = O), 166.4 (OC = O), 162.5 (ipso), 131.6 (Ar), 123.1 (ipso), 114.3 (Ar), 70.4 (C-3), 69.6 (C-2), 67.6 (C-1), 64.4 (C-1´), 50.8 (C-4), 43.8 (C-5´), 28.7 (C-2´), 25.8, 21.8 (C-3´, C-4´); MS: Calcd for [C_17_H_23_N_3_O_5_Na]: *m/z* 372.1535 [M + Na]^+^; Found [M + Na]^+^ 372.1557.

*6-Chlorohexyl 4-((tetrahydro-2H-pyran-2-yl)oxy)benzoate* (**31**). According to general procedure A, carboxylic acid **26** [54] (305 mg, 1.37 mmol) was reacted with 6-chlorohexanol (183 µL, 1.37 mmol), PPh_3_ (359 mg, 1.37 mmol) and DIAD (269 µL, 1.37 mmol). Silica gel chromatography (cyclohexane-ethyl acetate 13:1) afforded structure **31** (240 mg, 0.704 mmol, 51.4%) as a colourless syrup. ^1^H-NMR (300 MHz, CDCl_3_) δ = 7.97 (d, 2 H, Ar), 7.06 (d, 2 H, Ar), 5.49 (dd, 1 H, THP), 4.28 (t, 2 H, H-1´), 3.84 (m, 1 H, THP), 3.59 (m, 1 H, THP), 3.52 (t, 2 H, H-6´), 2.10-1.40 (po, 14 H, H-2´, H-3´, H-4´, H-5´, 3x CH_2_-THP); ^13^C-NMR (75.5 MHz, CDCl_3_) δ = 166.4 (OC = O), 160.9 (ipso), 131.5 (Ar), 123.6 (ipso), 115.9 (Ar), 96.1 (THP), 64.6 (C-1´), 62.1 (THP), 45.0 (C-6´), 32.6, 30.2, 28.7, 26.6, 25.5, 25.2 (C-2´, C-3´, C-4´, C-5´, 2x THP), 18.6 (THP); MS: Calcd for [C_18_H_25_ClO_4_Na]: *m/z* 363.1339 [M + Na]^+^; Found [M + Na]^+^ 363.1337.

*6-Chlorohexyl 4-hydroxybenzoate* (**32**). A stirred solution of **31** (102 mg, 0.299 mmol) in dioxane/H_2_O (1:1 *v*/*v*, 6 mL) was treated with acidic ion exchange resin (Amberlite^®^ IR-120H^+^ washed with H_2_O) at 40 °C. After completed removal of the THP group was detected, the reaction mixture was filtered off and the solvents were removed *in vacuo*. Purification on silica gel (cyclohexane-ethyl acetate 10:1) afforded **32** (73.7 mg, 0.287 mmol, 96.0%) as a colourless wax. ^1^H-NMR (300 MHz, CDCl_3_) δ = 7.95 (d, 2 H, Ar), 6.89 (d, 2 H, Ar), 6.52 (s, 1 H, OH), 4.30 (t, 2 H, H-1´), 3.54 (t, 2 H, H-6´), 1.79 (po, 4 H, H-2´, H-5´) 1.49 (po, 4 H, H-3´, H-4´); ^13^C-NMR (75.5 MHz, CDCl_3_) δ = 167.2 (OC = O), 160.4 (ipso), 132.1 (Ar), 122.7 (ipso), 115.4 (Ar), 65.0 (C-1´), 45.1 (C-6´), 32.6 (C-5´), 28.7 (C-2´), 26.7, 25.5 (C-3´, C-4´); MS: Calcd for [C_13_H_17_ClO_3_Na]: *m/z* 279.0764 [M + Na]^+^; Found [M + Na]^+^ 279.0765. 

*6-Chlorohexyl 4-(propargyloxy)benzoate* (**33**). To a stirred solution of **32** (1.33 g, 5.18 mmol) in acetone (40 mL), K_2_CO_3_ (2.86 g, 20.7 mmol) and 3-bromoprop-1-yne (669 µL, 6.21 mmol, 80% in toluene) were added. After completed conversion of the starting material, the reaction mixture was filtrated, diluted with CH_2_Cl_2_ and washed consecutively with HCl (2 *N*) and saturated aqueous NaHCO_3_. After drying over Na_2_SO_4_, the filtrate was evaporated to dryness. Purification on silica gel (cyclohexane-ethyl acetate 15:1) provided compound **33** (1.25 g, 4.24 mmol, 81.9%) as a colourless syrup. ^1^H-NMR (300 MHz, CDCl_3_) δ = 8.00 (d, 2 H, Ar), 6.99 (d, 2 H, Ar), 4.74 (d, 2 H, H-1), 4.29 (t, 2 H, H-1´), 3.54 (t, 2 H, H-6´), 2.55 (s, 1 H, H-3), 1.77 (po, 4 H, H-2´, H-5´) 1.49 (po, 4 H, H-3´, H-4´); ^13^C-NMR (75.5 MHz, CDCl_3_) δ = 166.3 (OC = O), 161.3 (ipso), 131.6 (Ar), 123.9 (ipso), 114.6 (Ar), 78.0 (C-2), 76.2 (C-3), 64.7 (C-1´), 56.0 (C-1), 45.1 (C-6´), 32.6 (C-5´), 28.8 (C-2´), 26.7, 25.6 (C-3´, C-4´); MS: Calcd for [C_16_H_19_ClO_3_Na]: *m/z* 317.0920 [M + Na]^+^; Found [M + Na]^+^ 317.0922. 

*6-Hydroxyhexyl 4-(propargyloxy)benzoate* (**34**). Following general procedure B2, halocarbon **33** (451 mg, 1.53 mmol) was treated with NaHCO_3_ (514 mg, 6.12 mmol). Separation on silica gel (cyclohexane-ethyl acetate 10:1) provided alcohol **34** (322 mg, 1.17 mmol, 76.5%) as a colourless oil. ^1^H-NMR (300 MHz, CDCl_3_) δ = 7.98 (d, 2 H, Ar), 6.97 (d, 2 H, Ar), 4.71 (d, 2 H, H-1), 4.26 (t, 2 H, H-1´), 3.61 (t, 2 H, H-6´), 2.55 (s, 1 H, H-3), 2.00 (s, 1 H, OH), 1.74 (m, 2 H, H-2´), 1.55 (m, 2 H, H-5´), 1.42 (po, 4 H, H-3´, H-4´); ^13^C-NMR (75.5 MHz, CDCl_3_) δ = 166.4 (OC = O), 161.2 (ipso), 131.6 (Ar), 123.8 (ipso), 114.5 (Ar), 77.9 (C-2), 76.2 (C-3), 64.8 (C-1´), 62.7 (C-6´), 55.9 (C-1), 32.6 (C-5´), 28.8 (C-2´), 25.9, 25.5 (C-3´, C-4´); MS: Calcd for [C_16_H_20_O_4_Na]: *m/z* 299.1259 [M + Na]^+^; Found [M + Na]^+^ 299.1259. 

*6-Oxohexyl 4-(propargyloxy)benzoate* (**35**). According to general procedure C, alcohol **34** (355 mg, 1.28 mmol) was oxidised employing Dess- Martin periodinane (598 mg, 1.41 mmol). Purification on silica gel (cyclohexane-ethyl acetate 10:1) realised aldehyde **35** (243 mg, 0.886 mmol, 69.2%) as amorphous solid. Recrystallisation from cyclohexane/ethyl acetate afforded colourless crystals (CCDC 2021385, see SM Figure S1). m.p.: 64-66 °C; ^1^H-NMR (300 MHz, CDCl3) δ = 9.76 (s, 1 H, HC = O), 7.99 (d, 2 H, Ar), 6.99 (d, 2 H, Ar), 4.73 (d, 2 H, H-1), 4.28 (t, 2 H, H-1´), 2.54 (s, 1 H, H-3), 2.46 (m, 2 H, H-5´), 1.80-1.65 (po, 4 H, H-2´, H-4´), 1.47 (m, 2 H, H-3´); ^13^C-NMR (75.5 MHz, CDCl3) δ = 202.4 (HC = O), 166.3 (OC = O), 161.3 (ipso), 131.6 (Ar), 123.8 (ipso), 114.6 (Ar), 77.9 (C-2), 76.2 (C-3), 64.5 (C-1´), 55.9 (C-1), 43.8 (C-5´), 28.7 (C-2´), 25.8 (C-3´), 21.8 (C-4´); MS: Calcd for [C16H18O4Na]: *m*/*z* 297.1103 [M + Na]^+^; Found [M + Na]^+^ 297.1104.

*Methyl 4-(2-(2-((nonafluoro-tert-butyl)oxy)ethoxy)ethoxy)benzoate* (**37**). Following general procedure A, compound **36** [59] (1.30 g, 5.41 mmol) was treated with nonafluoro-*tert*-butyl alcohol (754 µL, 5.41 mmol), PPh_3_ (1.42 g, 5.41 mmol) and DIAD (1.06 mL, 5.41 mmol). Purification on silica gel (cyclohexane-ethyl acetate 20:1) provided compound **37** (1.92 g, 4.19 mmol, 77.4%) as a colourless syrup. ^1^H-NMR (300 MHz, CDCl_3_) δ = 7.93 (d, 2 H, Ar), 6.86 (d, 2 H, Ar), 4.12 (po, 2 H, H-1), 4.08 (po, 2 H, H-4), 3.82 (po, 5 H, H-2, OCH_3_), 3.74 (m, 2 H, H-3); ^13^C-NMR (75.5 MHz, CDCl_3_) δ = 166.8 (OC = O), 162.6 (ipso), 131.6 (Ar), 122.9 (ipso), 120.4 (q, 3 C, *J*_C,F_ 292.5 Hz, CF_3_), 114.1 (Ar), 79.9 (q, 1 C, *J*_C,F_ 29.7 Hz, C(CF_3_)_3_), 69.9, 69.7, 69.4, 67.6 (C-1, C-2, C-3, C-4), 51.6 (OCH_3_).

*6-Hydroxyhexyl 4-(2-(2-((nonafluoro-tert-butyl)oxy)ethoxy)ethoxy)benzoate* (**40**). A solution of **37** (700 mg, 1.53 mmol) in 40 mL dioxane/H_2_O (3:1 *v*/*v*) was treated with solid NaOH (306 mg, 7.64 mmol) and stirred until completed conversion of the starting material was detected. After neutralisation with acidic ion exchange resin (Amberlite^®^ IR-120H^+^ washed with H_2_O), the reaction mixture was filtrated and evaporated to dryness providing intermediate **38**. This compound was directly converted to halocarbon **39** following general procedure A employing 6-bromohexanol (200 µL, 1.53 mmol), PPh_3_ (401 mg, 1.53 mmol) and DIAD (300 µL, 1.53 mmol). Due to problems during the separation of the resulting reaction mixture, crude product **39** was immediately hydrolysed according to general procedure B2 employing NaHCO_3_ (514 mg, 6.12 mmol). Purification on silica gel (cyclohexane-ethyl acetate 10:1) provided alcohol **40** (412 mg, 0.757 mmol, 49.5% over 3 steps) as a colourless oil. ^1^H-NMR (300 MHz, CDCl_3_) δ = 7.98 (d, 2 H, Ar), 6.92 (d, 2 H, Ar), 4.28 (t, 2 H, H-1´), 4.16 (po, 4 H, H-1, H-4), 3.89 (m, 2 H, H-2), 3.80 (m, 2 H, H-3), 3.64 (t, 2 H, H-6´), 1.94 (bs, 1 H, OH), 1.76 (m, 2 H, H-2´), 1.59 (m, 2 H, H-5´), 1.45 (po, 4 H, H-3´, H-4´); ^13^C-NMR (75.5 MHz, CDCl_3_) δ = 166.6 (OC = O), 162.6 (ipso), 131.7 (Ar), 123.3 (ipso), 120.3 (q, 3 C, *J*_C,F_ 293.0 Hz, CF_3_), 114.3 (Ar), 79.8 (q, 1 C, *J*_C,F_ 28.9 Hz, C(CF_3_)_3_), 70.1 (C-3), 69.8 (C-2), 69.5, 67.7 (C-1, C-4), 64.8 (C-1´), 62.9 (C-6´), 32.8 (C-5´), 28.9 (C-2´), 26.0, 25.6 (C-3´, C-4´).

*6-Oxohexyl 4-(2-(2-((nonafluoro-tert-butyl)oxy)ethoxy)ethoxy)*benzoate (**41**). Following general procedure C, alcohol **40** (91.0 mg, 0.167 mmol) was treated with Dess-Martin periodinane (78.0 mg, 0.184 mmol). Purification on silica gel (cyclohexane-ethyl acetate 15:1) afforded aldehyde **41** (76.2 mg, 0.140 mmol, 83.8%) as a colourless oil. ^1^H-NMR (300 MHz, CDCl_3_) δ = 9.75 (s, 1 H, HC = O), 7.96 (d, 2 H, Ar), 6.91 (d, 2 H, Ar), 4.27 (t, 2 H, H-1´), 4.15 (po, 4 H, H-1, H-4), 3.88 (m, 2 H, H-2), 3.79 (m, 2 H, H-3), 2.45 (m, 2 H, H-5´), 1.83-1.63 (po, 4 H, H-2´, H-4´) 1.46 (m, 2 H, H-3´); ^13^C-NMR (75.5 MHz, CDCl_3_) δ = 202.4 (HC = O), 166.4 (OC = O), 162.6 (ipso), 131.6 (Ar), 123.2 (ipso), 120.4 (q, 3 C, *J*_C,F_ 292.6 Hz, CF_3_), 114.3 (Ar), 79.9 (q, 1 C, *J*_C,F_ 29.7 Hz, C(CF_3_)_3_), 70.1 (C-3), 69.8 (C-2), 69.5, 67.7 (C-1, C-4), 64.5 (C-1´), 43.8 (C-5´), 28.7 (C-2´), 25.8 (C-3´), 21.8 (C-4´).

*6-Bromohexyl 4-(2-(((benzyloxy)carbonyl)amino)ethyl)benzoate* (**43**). According to general procedure A, literature known compound **42** [60] (585 mg, 1.96 mmol) was treated with 6-bromohexanol (256 µl, 1.96 mmol), PPh_3_ (514 mg, 1.96 mmol) and DIAD (385 µl, 1.96 mmol). Silica gel chromatography (cyclohexane-ethyl acetate 13:1) provided structure **43** (615 mg, 1.33 mmol, 67.9%) as a slightly yellow syrup. ^1^H-NMR (300 MHz, CDCl_3_) δ = 7.89 (d, 2 H, Ar), 7.26 (s, 5 H, NH-COOCH_2_Ph), 7.17 (d, 2 H, Ar), 5.02 (s, 2 H, NH-COOCH_2_Ph), 4.72 (bs, 1 H, NH), 4.24 (t, 2 H, H-1´), 3.37 (po, 4 H, H-2, H-6´), 2.80 (m, 2 H, H-1), 1.80 (m, 2 H, H-5´), 1.69 (m, 2 H, H-2´), 1.38 (po, 4 H, H-3´, H-4´); ^13^C-NMR (75.5 MHz, CDCl_3_) δ = 166.6 (OC = O), 156.4 (NH-COOCH_2_Ph), 144.2 (ipso), 136. 6 (ipso), 130.0-128.2 (Ar), 66.9 (NH-COOCH_2_Ph), 64.9 (C-1´), 42.0 (C-2), 36.3 (C-1), 33.8 (C-6´), 32.8 (C-5´), 28.7 (C-2´), 28.0, 25.4 (C-3´, C-4´); MS: Calcd for [C_23_H_28_BrNO_4_Na]: *m/z* 484.1099 [M + Na]^+^; Found [M + Na]^+^ 484.1098. 

*6-Oxohexyl 4-(2-(((benzyloxy)carbonyl)amino)ethyl)benzoate* (**44**). Following general procedure B1, halocarbon **43** (798 mg, 1.73 mmol) was treated with NaHCO_3_ (874 mg, 10.4 mmol). Purification on silicagel (cyclohexane-ethyl acetate 8:1) gave aldehyde **44** (335 mg, 0.843 mmol, 48.7%) as a colourless oil. ^1^H-NMR (300 MHz, CDCl_3_) δ = 9.70 (s, 1 H, HC = O), 7.89 (d, 2 H, Ar), 7.27 (s, 5 H, NH-COOCH_2_Ph), 7.18 (d, 2 H, Ar), 5.02 (s, 2 H, NH-COOCH_2_Ph), 4.71 (bs, 1 H, NH), 4.24 (t, 2 H, H-1´), 3.40 (m, 2 H, H-2), 2.81 (m, 2 H, H-1), 2.40 (t, 2 H, H-5´), 1.75-1.57 (po, 4 H, H-2´, H-4´), 1.41 (m, 2 H, H-3´); ^13^C-NMR (75.5 MHz, CDCl_3_) δ = 202.4 (HC = O), 166.6 (OC = O), 156.4 (NH-COOCH_2_Ph), 144.3 (ipso), 136.6 (ipso), 130.0-128.2 (Ar), 66.9 (NH-COOCH_2_Ph), 64.7 (C-1´), 43.9 (C-5´), 42.0 (C-2), 36.3 (C-1), 28.7 (C-2´), 25.8 (C-3´), 21.8 (C-4´); MS: Calcd for [C_23_H_27_NO_5_Na]: *m/z* 420.1787 [M + Na]^+^; Found [M + Na]^+^ 420.1783. 

*N-(6-((4-((5-Cyanopentyl)oxy)benzoyl)oxy)hexyl)-1,5-dideoxy-1,5-imino-d-glucitol* (**45**). A solution of aldehyde **25** (60.2 mg 0.182 mmol), AcOH (25 µL) and 1-deoxynojirimycin (**1** [61], 38.6 mg, 0.237 mmol) in 3 mL MeOH was stirred with Pd/C (10%) under an atmosphere of H_2_ at ambient pressure until completed conversion of the starting material. After removal of the catalyst, the filtrate was concentrated under reduced pressure. Purification on silica gel (ethyl acetate-MeOH 8:1) provided compound **45** (55.1 mg, 0.115 mmol, 63.2%) as amorphous white solid. ${\left[a\right]}_{D}^{20}$: −5.4 (c = 1.22, MeOH); ^1^H-NMR (300 MHz, CD_3_OD) δ = 7.91 (d, 2 H, Ar), 6.94 (d, 2 H, Ar), 4.25 (t, 2 H, H-6´), 4.02 (t, 2 H, H-1´´), 3.89 (m, 2 H, H-6a, H-6b), 3.55 (ddd, 1 H, *J*_1a,2_ 4.9 Hz, *J*_1b,2_ 9.8 Hz, *J*_2,3_ 8.7 Hz, H-2), 3.44 (dd, 1 H, *J*_4,5_ 9.3 Hz, H-4), 3.18 (po, 2 H, H-1a, H-3), 3.01 (m, 1 H, H-1´), 2.82 (m, 1 H, H-1´), 2.46 (po, 4 H, H-1b, H-5, H-5´´), 1.87-1.29 (po, 14 H, H-2´, H-3´, H-4´, H-5´, H-2´´, H-3´´, H-4´´); ^13^C-NMR (75.5 MHz, CD_3_OD) δ = 168.0 (OC = O), 164.5 (ipso), 132.5 (Ar), 123.6 (ipso), 121.1 (CN), 115.3 (Ar), 79.5 (C-3), 70.7 (C-4), 69.5 (C-2), 68.9 (C-1´´), 67.4 (C-5), 65.7 (C-6´), 57.7 (C-6), 56.4 (C-1), 53.8 (C-1´), 29.7, 29.4, 27.8, 26.9, 26.3, 26.2, 24.7 (C-2´, C-3´, C-4´, C-5´, C-2´´, C-3´´, C-4´´), 17.3 (C-5´´); MS: Calcd for [C_25_H_38_N_2_O_7_Na]: *m/z* 501.2577 [M + Na]^+^; Found [M + Na]^+^ 501.2578. 

*N-(6-((4-((5-Cyanopentyl)oxy)benzoyl)oxy)hexyl)-1,5-dideoxy-1,5-imino-d-xylitol* (**46**). Method A: Following general procedure D, aldehyde **25** (30.5 mg, 92.0 µmol) was treated with 1,5-imino-1,5-dideoxy-d-xylitol (2 [62], 13.4 mg, 0.101 mmol) and NaBH_3_CN (17.3 mg, 0.276 mmol). Purification on silica gel (ethyl acetate-MeOH 8:1) provided compound **46** (29.3 mg, 65.3 µmol, 71.0%) as a white solid. ^1^H-NMR (300 MHz, CD_3_OD) δ = 7.96 (d, 2 H, Ar), 6.99 (d, 2 H, Ar), 4.29 (t, 2 H, H-6´), 4.08 (m, 2 H, H-1´´), 3.50 (ddd, 2 H, *J*_1a/5a,2/4_ 4.5 Hz, J_1b/5b,2/4_ 10.0 Hz *J*_2/4,3_ 8.9 Hz, H-2/4), 3.11 (dd, 1 H, H-3), 2.99 (dd, 2 H, *J*_1a/1b,5a/5b_ 10.6 Hz H-1a/5a), 2.46 (po, 4 H, H-1´, H-5´´), 1.94 (dd, 2 H, H-1b/5b), 1.88-1.36 (po, 14 H, H-2´, H-3´, H-4´, H-5´, H-2´´, H-3´´, H-4´´); ^13^C-NMR (75.5 MHz, CD_3_OD) δ = 168.0 (OC = O), 164.5 (ipso), 132.5 (Ar), 123.7 (ipso), 121.1 (CN), 115.3 (Ar), 80.3 (C-3), 71.4 (C-2/4), 68.9 (C-1´´), 65.8 (C-6´), 59.4 (C-1/5), 58.9 (C-1´), 29.8, 29.4, 28.1, 27.6, 27.0, 26.4, 26.3, (C-2´, C-3´, C-4´, C-5´, C-2´´, C-3´´, C-4´´), 17.3 (C-5´´); MS: Calcd for [C_24_H_36_N_2_O_6_H]: *m/z* 449.2652 [M + H]^+^; Found [M + H]^+^ 449.2649. 

Method B: A solution of aldehyde **25** (51.0 mg 0.154 mmol), AcOH (20 µL) and iminosugar **2** (24.6 mg, 0.185 mmol) in 2.5 mL MeOH was stirred with Pd/C (10%) under an atmosphere of H_2_ at ambient pressure. After completed conversion of the starting material, the reaction mixture was filtrated and concentrated under reduced pressure. Purification on silica gel (ethyl acetate-MeOH 8:1) gave compound **46** (33.0 mg, 73.6 µmol, 47.8%) as a white solid.

*N-(6-((4-(2-(2-Azidoethoxy)ethoxy)benzoyl)oxy)hexyl)-1,5-dideoxy-1,5-imino-d-glucitol* (**47**). Following general procedure D, aldehyde **30** (98.0 mg, 0.280 mmol) was treated with iminosugar **1** (54.8 mg, 0.336 mmol) and NaBH_3_CN (35.2 mg, 0.560 mmol). Purification on silica gel (ethyl acetate-MeOH 8:1) provided compound **47** (78.1 mg, 0.157 mmol, 56.1%) as a white solid. ${\left[a\right]}_{D}^{20}$: -3.4 (c = 1.47, MeOH); ^1^H-NMR (300 MHz, CD_3_OD) δ = 7.98 (d, 2 H, Ar), 7.04 (d, 2 H, Ar), 4.30 (t, 2H, H-6´), 4.23 (m, 2 H, H-1´´), 3.89 (po, 4 H, H-2´´,H-6a, H-6b), 3.76 (m, 2 H, H-3´´), 3.52 (ddd, 1 H, *J*_1a,2_ 4.7 Hz, *J*_1b,2_ 10.2 Hz, H-2), 3.40 (po, 3 H, H-4, H-4´´), 3.19 (dd, 1 H, *J*_2,3_ = *J*_3,4_ 9.1 Hz, H-3), 3.08 (dd, 1 H, H-1a), 2.91 (m, 1 H, H-1´), 2.70 (m, 1 H, H-1´), 2.32 (po, 2 H, H-1b, H-5), 1.80 (m, 2 H, H-5´), 1.68-1.34 (po, 6 H, H-2´, H-3´, H-4´); ^13^C-NMR (75.5 MHz, CD_3_OD) δ = 168.0 (OC = O), 164.3 (ipso), 132.5 (Ar), 123.9 (ipso), 115.4 (Ar), 80.2 (C-3), 71.6 (C-4), 71.4 (C-3´´), 70.6 (C-2´´), 70.3 (C-2), 68.9 (C-1´´), 67.4 (C-5), 65.8 (C-6´), 58.9 (C-6), 57.3 (C-1), 53.7 (C-1´), 51.8 (C-4´´), 29.8 (C-5´), 28.1, 27.0, 25.1 (C-2´, C-3´, C-4´); MS: Calcd for [C_23_H_36_N_4_O_8_Na]: *m/z* 519.2431 [M + Na]^+^; Found [M + Na]^+^ 519.2431.

*N-(6-((4-(2-(2-Azidoethoxy)ethoxy)benzoyl)oxy)hexyl)-1,5-dideoxy-1,5-imino-d-xylitol* (**48**). According to general procedure D, aldehyde **30** (25.0 mg, 71.6 µmol) was treated with iminosugar **2** (11.4 mg, 85.9 µmol) and NaBH_3_CN (13.5 mg, 0.215 mmol). Purification on silica gel (ethyl acetate-MeOH 8:1) afforded xylitol derivative **48** (18.1 mg, 38.8 µmol, 54.2%) as a white solid. ^1^H-NMR (300 MHz, CD_3_OD) δ = 8.01 (d, 2 H, Ar), 7.05 (d, 2 H, Ar), 4.31 (t, 2 H, H-6´), 4.24 (m, 2 H, H-1´´), 3.90 (m, 2 H, H-2´´), 3.77 (m, 2 H, H-3´´), 3.53 (ddd, 2 H, *J*_2/4,1a/5a_ 9.6 Hz, *J*_2/4,1b/5b_ 3.3 Hz, *J*_2/4,3_ 8.5 Hz, H-2/4), 3.42 (m, 2 H, H-4´´), 3.15 (dd, 1 H, H-3), 3.03 (dd, 2 H, *J*_1a/5a,1b/5b_ 10.9 Hz, H-1a/5a), 2.51 (m, 2 H, H-1´), 2.04 (dd, 2 H, H-1b/5b), 1.81 (m, 2 H, H-5´), 1.66-1.37 (po, 6 H, H-2´, H-3´, H-4´); ^13^C-NMR (75.5 MHz, CD_3_OD) δ = 168.0 (OC = O), 164.3 (ipso), 132.5 (Ar), 124.0 (ipso), 115.4 (Ar), 79.1 (C-3), 71.4 (C-3´´), 71.2 (C-2/4), 70.6 (C-2´´), 68.9 (C-1´´), 65.8 (C-6´), 59.1 (C-1/5), 58.8 (C-1´), 51.8 (C-4´´), 29.8 (C-5´), 28.1, 27.4, 27.0 (C-2´, C-3´, C-4´); MS: Calcd for [C_22_H_34_N_4_O_7_H]: *m/z* 467.2506 [M + H]^+^; Found [M + H]^+^ 467.2507. 

*N-(6-((4-(2-(2-(Nonafluoro-tert-butyloxy)ethoxy)ethoxy)benzoyl)oxy)hexyl)-1,5-dideoxy-1,5-imino-d-glucitol* (**49**). Following general procedure D, aldehyde **41** (52.0 mg, 95.9 µmol) was treated with iminosugar **1** (18.8 mg, 0.115 mmol) and NaBH_3_CN (18.1 mg, 0.288 mmol). Purification on silica gel (ethyl acetate-MeOH 8:1) gave structure **49** (34.3 mg, 49.7 µmol, 51.8%) as a white solid. ${\left[a\right]}_{D}^{20}$: -4.4 (c = 1.17, MeOH); ^1^H-NMR (300 MHz, CD_3_OD) δ = 7.96 (d, 2 H, Ar), 7.03 (d, 2 H, Ar), 4.31 (m, 2 H, H-6´), 4.22 (po, 4 H, H-1´´, H-4´´), 3.85 (po, 6 H, H-2´´, H-3´´, H-6a, H-6b), 3.51 (ddd, 1 H, *J*_1a, 2_ 4.8 Hz, *J*_1b,2_ 10.1 Hz, H-2), 3.38 (dd, 1 H, *J*_3,4_ = *J*_4,5_ 9.2Hz, H-4), 3.17 (dd, 1 H, *J*_2,3_ 9 Hz, H-3), 3.05 (dd, 1 H, H-1), 2.88 (m, 1 H, H-1´), 2.67 (m, 1 H, H-1´), 2.25 (po, 2 H, H-1b, H-5), 1.80 (m, 2 H, H-5´), 1.66-1.34 (po, 6 H, H-2´, H-3´, H-4´); ^13^C-NMR (75.5 MHz, CD_3_OD) δ = 168.0 (OC = O), 164.3 (ipso), 132.5 (Ar), 124.0 (ipso), 123.7, 121.8 (q, 3 C, *J*_C,F_ 291.9 Hz, CF_3_), 115.4 (Ar), 80.4 (C-3), 71.8 (C-4), 71.2, 70.9, 70.7, 68.9 (C-1´´, C-2´´, C-3´´, C-4´´), 70.5 (C-2), 67.4 (C-5), 65.8 (C-6´), 59.2 (C-6), 57.5 (C-1), 53.7 (C-1´), 29.8 (C-5´), 28.2, 27.0, 25.1 (C-2´, C-3´, C-4´); MS: Calcd for [C_27_H_36_F_9_NO_9_H]: *m/z* 690.2325 [M + H]^+^; Found [M + H]^+^ 690.2316. 

*N-(6-((4-(2-(2-(Nonafluoro-tert-butyloxy)ethoxy)ethoxy)benzoyl)oxy)hexyl)-1,5-dideoxy-1,5-imino-d-xylitol* (**50**). Following general procedure D, aldehyde **41** (43.0 mg, 79.3 µmol) was treated with iminosugar **2** (11.6 mg, 95.2 µmol) and NaBH_3_CN (15.0 mg, 0.238 mmol). Purification on silica gel (ethyl acetate-MeOH 8:1) provided compound **50** (32.4 mg, 49.2 µmol, 62.0%) as a white solid.^1^H-NMR (300 MHz, CD_3_OD) δ = 7.97 (d, 2 H, Ar), 7.02 (d, 2 H, Ar), 4.31 (t, 2H, H-6´), 4.22 (po, 4 H, H-1´´, H-4´´), 3.87 (t, 2 H, H-2´´), 3.82 (t, 2 H, H-3´´), 3.56 (ddd, 2 H, *J*_1a/5a,2_ 3.5 Hz, *J*_1b/5b,2_ 9.1 Hz, *J*_2/4,3_ 8.4 Hz, H-2/4), 3.21 (dd, 1 H, H-3), 3.07 (dd, 2 H, *J*_1a/5a,1b/5b_ 10.9 Hz, H-1a/5a), 2.60 (m, 2 H, H-1´), 2.18 (dd, 2 H, H-1b/5b), 1.80 (m, 2 H, H-5´), 1.68-1.35 (po, 6 H, H-2´, H-3´, H-4´); ^13^C-NMR (75.5 MHz, CD_3_OD) δ = 168.0 (OC = O), 164.3 (ipso), 132.5 (Ar), 124.0 (ipso), 121.8 (q, 3 C, *J*_C,F_ 290.9 Hz, CF_3_), 115.4 (Ar), 79.1 (C-3), 71.1, 70.9, 70.9, 70.7, 68.9 (C-2/4, C-1´´, C-2´´, C-3´´, C-4´´), 65.8 (C-6´), 58.7, 58.6 (C-1/5, C-1´), 29.8 (C-5´), 28.0, 27.1, 26.9 (C-2´, C-3´, C-4´); MS: Calcd for [C_26_H_34_F_9_NO_8_Na]: *m/z* 682.2039 [M + Na]^+^; Found [M + Na]^+^ 682.2039. 

*N-(6-((4-(Propargyloxy)benzoyl)oxy)hexyl)-1,5-dideoxy-1,5-imino-d-glucitol* (**51**). Following general procedure D, aldehyde **35** (62.6 mg 0.228 mmol) was treated with iminosugar **1** (37.2 mg, 0.228 mmol) and NaBH_3_CN (21.5 mg, 0.342 mmol). Silica gel chromatography (ethyl acetate-MeOH 7:1) gave structure **51** (57.4 mg, 0.136 mmol, 59.6%) as a white solid. ${\left[a\right]}_{D}^{20}$: -6.0 (c = 1.08, MeOH); ^1^H-NMR (300 MHz, CD_3_OD) δ = 7.98 (d, 2 H, Ar), 7.07 (d, 2 H, Ar), 4.82 (m, 2 H, H-1´´), 4.30 (m, 2 H, H-6´), 3.88 (m, 2 H, H-6a, H-6b), 3.52 (ddd, 1 H, *J*_1a,2_ 4.9 Hz, *J*_1b,2_ 9.7 Hz, H-2), 3.39 (dd, 1 H, *J*_3,4_ = *J*_4,5_ 9.3 Hz, H-4), 3.18 (dd, 1 H, *J*_2,3_ 9.0 Hz, H-3), 3.11 (dd, 1 H, *J*_1a,1b_ 11.3 Hz, *J*_1a,2_ 4.9 Hz, H-1a), 3.02-2.90 (m, 2 H, H-1´, H-3´´), 2.72 (m, 1 H, H-1´), 2.35 (po, 2 H, H-1b, H-5), 1.78 (m, 2 H, H-5´), 1.69-1.32 (po, 6 H, H-2´, H-3´, H-4´); ^13^C-NMR (75.5 MHz, CD_3_OD) δ = 167.9 (OC = O), 163.0 (ipso), 132.4 (Ar), 124.5 (ipso), 115.8 (Ar), 80.0 (C-3), 79.1 (C-2´´), 77.3 (C-3´´), 71.3 (C-4), 70.1 (C-2), 67.4 (C-5), 65.8 (C-6´), 58.5 (C-6), 57.0 (C-1), 56.8 (C-1´´), 53.7 (C-1´), 29.8 (C-5´), 28.0, 27.0, 25.0 (C-2´, C-3´, C-4´); MS: Calcd for [C_22_H_31_NO_7_H]: *m/z* 422.2179 [M + H]^+^; Found [M + H]^+^ 422.2179.

*N-(6-((4-(Propargyloxy)benzoyl)oxy)hexyl)-1,5-dideoxy-1,5-imino-d-xylitol* (**52**). Following general procedure D, aldehyde **35** (104 mg, 0.379 mmol) was treated with iminosugar **2** (50.5 mg, 0.379 mmol) and NaBH_3_CN (35.8 mg, 0.569 mmol). Purification on silica gel (ethyl acetate-MeOH 10:1) afforded compound **52** (52.4 mg, 0.134 mmol, 35.4%) as a white solid.^1^H-NMR (300 MHz, CD_3_OD) δ = 7.99 (d, 2 H, Ar), 7.08 (d, 2 H, Ar), 4.83 (m, 2 H, H-1´´), 4.30 (t, 2 H, H-6´), 3.54 (m, 2 H, H-2/4), 3.16 (dd, 1 H, *J*_2/4,3_ 8.1 Hz, H-3), 3.02 (po, 3 H, H-1a/5a, H-3´´), 2.48 (m, 2 H, H-1´), 2.02 (m, 2 H, H-1b/5b), 1.79 (m, 2 H, H-5´), 1.66-1.33 (po, 6 H, H-2´, H-3´, H-4´); ^13^C-NMR (75.5 MHz, CD_3_OD) δ = 167.8 (OC = O), 163.0 (ipso), 132.4 (Ar), 124.5 (ipso), 115.8 (Ar), 79.8 (C-3), 79.2 (C-2´´), 77.3 (C-3´´), 71.1 (C-2/4), 65.8 (C-6´), 59.1 (C-1/5), 58.8 (C-1´), 56.8 (C-1´´), 29.7 (C-5´), 28.1, 27.4, 27.0 (C-2´, C-3´, C-4´); MS: Calcd for [C_21_H_29_NO_6_Na]: *m/z* 414.1893 [M + Na]^+^; Found [M + Na]^+^ 414.1894. 

*N-(6-((4-(2-(((Benzyloxy)carbonyl)amino)ethyl)benzoyl)oxy)hexyl)-1,5-dideoxy-1,5-imino-d-glucitol* (**53**). Following general procedure D, aldehyde **44** (92.2 mg, 0.232 mmol) was treated with iminosugar **1** (37.9 mg, 0.232 mmol) and NaBH_3_CN (21.9 mg, 0.348 mmol). Purification on silica gel (ethyl acetate-MeOH 10:1) provided compound **53** (69.8 mg, 0.128 mmol, 55.2%) as a white solid. ${\left[a\right]}_{D}^{20}$: -5.0 (c = 1.04, MeOH); ^1^H-NMR (300 MHz, CD_3_OD) δ = 7.87 (d, 2 H, Ar), 7.27 (m, 7 H, Ar), 4.97 (s, 2 H, NH-COOCH_2_Ph), 4.27 (t, 2 H, H-6´), 3.93 (dd, 1 H, *J*_5,6a_ 2.5 Hz, *J*_6a,6b_ 12.3 Hz, H-6a,), 3.82 (dd, 1 H, *J*_5,6b_ 2.5 Hz, H-6b), 3.56 (ddd, 1 H, *J*_1a,2_ 3.9 Hz, *J*_2,3_ 9.9 Hz, H-2), 3.47 (dd, 1 H, *J*_3,4_ = *J*_4,5_ 9.5 Hz, H-4), 3.27 (po, 4 H, H-1a, H-3, H-1´´), 3.07 (m, 1 H, H-1´a), 2.90 (m, 1 H, H-1´b), 2.81 (m, 2 H, H-2´´), 2.62 (po, 2 H, H-1b, H-5), 1.73 (m, 2 H, H-5´), 1.64 (m, 2 H, H-2´), 1.55–1.29 (po, 4 H, H-3´,H-4´); ^13^C-NMR (75.5 MHz, CD_3_OD) δ = 168.1 (OC = O), 158.8 (NH-COOCH_2_Ph), 146.4 (ipso), 138.4 (ipso), 130.6-128.7 (Ar), 79.1 (C-3), 70.1 (C-4), 69.0 (C-2), 67.4 (C-5), 67.3 (NH-COOCH_2_Ph), 65.9 (C-6´), 57.0 (C-6), 55.9 (C-1), 53.8 (C-1´), 42.9 (C-1´´), 37.0 (C-2´´), 29.7 (C-5´), 27.7, 26.8, (C-3´, C-4´), 24.5 (C-5´); MS: Calcd for [C_29_H_40_N_2_O_8_Na]: *m/z* 567.2682 [M + Na]^+^; Found [M + Na]^+^ 567.2681. 

*N-(6-((4-(2-(((Benzyloxy)carbonyl)amino)ethyl)benzoyl)oxy)hexyl)-1,5-dideoxy-1,5-imino-D-xylitol* (**54**). Following general procedure D, aldehyde **44** (88.3 mg, 0.222 mmol) was treated with iminosugar **2** (29.6 mg, 0.222 mmol) and NaBH_3_CN (20.9 mg, 0.333 mmol). Silica gel chromatography (ethyl acetate-MeOH 7:1) provided compound **54** (30.6 mg, 59.5 µmol, 26.8%) as a white solid.^1^H-NMR (300 MHz, CD_3_OD) δ = 7.94 (d, 2 H, Ar), 7.33 (m, 7 H, Ar), 5.06 (s, 2 H, NH-COOCH_2_Ph), 4.32 (m, 2 H, H-6´), 3.51 (m, 2 H, *J*_1a/5a,2/4_ 4.4 Hz, *J*_1b/5b,2/4_ 9.8 Hz, *J*_2/4,3_ 8.9 Hz, H-2/4), 3.38 (m, 2 H, H-1´´), 3.10 (dd, 1 H, H-3), 2.99 (m, 2 H, *J*_1a/5a,1b/5b_ 10.7 Hz, H-1a/5a), 2.87 (m, 2 H, H-2´´), 2.43 (m, 2 H, H-1´), 1.94 (dd, 2 H, H-1b/5b), 1.80 (m, 2 H, H-5´), 1.63-1.35 (po, 6 H, H-2´, H-3´, H-4´); ^13^C-NMR (75.5 MHz, CD_3_OD) δ = 168.1 (OC = O), 158.8 (NH-COOCH_2_Ph), 146.4 (ipso), 138.5 (ipso), 130.6-128.7 (Ar), 80.4 (C-3), 71.4 (C-2/4), 67.3 (NH-COOCH_2_Ph), 66.0 (C-6´), 59.4 (C-1/5), 58.8 (C-1´), 42.9 (C-1´´), 37.0 (C-2´´), 29.7 (C-5´), 28.1, 27.6, 27.0 (C-2´, C-3´, C-4´); MS: Calcd for [C_28_H_38_N_2_O_7_Na]: *m/z* 537.2577 [M + Na]^+^; Found [M + Na]^+^ 537.2578. 

*N-(6-((4-(2-(Dansylamino)ethyl)benzoyl)oxy)hexyl)-1,5-dideoxy-1,5-imino-d-glucitol* (**55**). A 10% solution of *N*-Cbz protected compound **53** (84.9 mg, 0.156 mmol) in MeOH containing AcOH (20 µL) was stirred with Pd/C (10%) under an atmosphere of H_2_ at ambient pressure until completed liberation of the corresponding amine was detected. After removal of the catalyst, the filtrate was immediately treated with Et_3_N (100 µL) and dansyl chloride (46.4 mg, 0.172 mmol). Additional stirring for 90 min, evaporation of the solvents followed by purification on silica gel (ethyl acetate-MeOH 3:1) provided title compound **55** (50.2 mg, 78.0 µmol, 50.0% over 2 steps) as a slightly yellow wax. ${\left[a\right]}_{D}^{20}$: -5.6 (c = 1.08, MeOH); ^1^H-NMR (300 MHz, CD_3_OD) δ = 8.53 (d, 1 H, dansyl), 8.19 (m, 2 H, dansyl), 7.67 (d, 2 H, Ar), 7.52 (m, 2 H, dansyl), 7.20 (d, 1 H, dansyl), 7.00 (d, 2 H, Ar), 4.29 (t, 2H, H-6´), 3.88 (m, 2 H, H-6a, H-6b), 3.51 (ddd, 1 H, *J*_1b,2_ 10.0 Hz, *J*_2,3_ 9.3 Hz, H-2), 3.35 (m, 1 H, H-4), 3.16 (po, 3 H, H-3, H-1´´), 3.01 (dd, 1 H, *J*_1a,1b_ 11.0 Hz, *J*_1b,2_ 4.7 Hz, H-1a), 2.84 (po, 7 H, H-1´, dansyl), 2.65 (po, 3 H, H-1´, H-2´´), 2.16 (po, 2 H, H-1b, H-5), 1.76 (m, 2 H, H-5´), 1.63-1.31 (po, 6 H, H-2´, H-3´, H-4´); ^13^C-NMR (75.5 MHz, CD_3_OD) δ = 168.0 (OC = O), 153.1-116.3 (dansyl, Ar), 80.5 (C-3), 72.0 (C-4), 70.7 (C-2), 67.4 (C-5), 66.0 (C-6´), 59.4 (C-6), 57.6 (C-1), 53.7 (C-1´), 45.8 (dansyl), 44.9 (C-1´´), 36.7 (C-2´´), 29.8 (C-5´), 28.2, 27.1, 25.2 (C-2´, C-3´, C-4´); MS: Calcd for [C_33_H_45_N_3_O_8_SNa]: *m/z* 666.2825 [M + Na]^+^; Found [M + Na]^+^ 666.2825. 

*N-(6-((4-(2-(Dansylamino)ethyl)benzoyl)oxy)hexyl)-1,5-dideoxy-1,5-imino-d-xylitol* (**56**). A 10% solution of *N*-Cbz protected compound **54** (54.0 mg, 0.105 mmol) in MeOH containing AcOH (20 µL) was stirred with Pd/C (10%) under an atmosphere of H_2_ at ambient pressure until completed liberation of the corresponding amine was detected. After removal of the catalyst, the filtrate was immediately treated with Et_3_N (100 µL) and dansyl chloride (31.3 mg, 0.116 mmol). Additional stirring for 90 min, evaporation of the solvents followed by purification on silica gel (ethyl acetate-MeOH 3:1) provided title compound **56** (28.7 mg, 46.8 µmol, 44.6% over 2 steps) as a slightly yellow wax. ^1^H-NMR (500 MHz, CD_3_OD) δ = 8.54 (d, 1 H, dansyl), 8.19 (m, 2 H, dansyl), 7.67 (d, 2 H, Ar), 7.52 (m, 2 H, dansyl), 7.23 (d, 1 H, dansyl), 7.00 (d, 2 H, Ar), 4.35 (t, 2 H, H-6´), 3.55 (ddd, 2 H, *J*_1a/5a,2/4_ 4.8 Hz, *J*_1b/5b,2/4_ 9.7 Hz, H-2/4), 3.24 (m, 2 H, H-1´´), 3.15 (dd, 1 H, *J*_2/4,3_ 8.8 Hz, H-3), 3.03 (dd, 2 H, *J*_1a/5a,1b/5b_ 10.8 Hz, H-1b/5b), 2.94 (s, 6 H, dansyl), 2.73 (m, 2 H, H-2´´), 2.47 (m, 2 H, H-1´), 1.97 (m, 2 H, H-1b, H-5b), 1.84 (m, 2 H, H-5´), 1.63-1.36 (po, 6 H, H-2´, H-3´, H-4´); ^13^C-NMR (75.5 MHz, CD_3_OD) δ = 168.0 (OC = O), 153.1-116.3 (dansyl, Ar), 80.5 (C-3), 71.4 (C-2/4), 66.0 (C-6´), 59.5 (C-1/5), 58.9 (C-1´), 45.8 (dansyl), 44.9 (C-1´´), 36.7 (C-2´´), 29.8 (C-5´), 28.2, 27.7, 27.0 (C-2´, C-3´, C-4´); MS: Calcd for [C_32_H_43_N_3_O_7_SNa]: *m/z* 636.2720 [M + Na]^+^; Found [M + Na]^+^ 636.2720. 

### 3.3. Kinetic Studies

Kinetic studies were performed at room temperature in an appropriate buffer for each enzyme (specific conditions can be found below). All the reactions were performed in half-area 96-well-plates (Corning, Corning, NY, USA) and monitored with a Synergy H1 plate reader (BioTek Instruments, Winooski, VT, USA). In each experiment, the appropriate concentration of the enzyme was incubated with different concentrations of the inhibitors for 2–5 min before initiating the reaction by the addition of substrate. The initial rate was then measured by monitoring the increase in absorbance as a result of the release of 4-nitrophenol at 405 nm for up to five minutes. *K*_i_ determinations were performed using two different substrate concentrations. For each substrate concentration, a range of three to six inhibitor concentrations was used. Dixon plots (1/v vs. [I]) were constructed to validate the use of competitive inhibition model and to assess the fit of the data. The data were then fit to a competitive inhibition model using non-linear regression analysis with Grafit 7.0.3 (Erithacus Software, East Grinstead, UK).

#### Specific Assay Conditions for Each Enzyme:

*Agrobacterium* sp. β-glucosidase (Abg): [64,65] 50 mM sodium phosphate buffer (pH 7). Substrate: *p*NP β-Gal, *K*_m_ = 4.1 mM.

*E. coli* lac *z* β-galactosidase: 50 mM sodium phosphate, 1.0 mM MgCl_2_ (pH 7). Substrate: *p*NP β-Gal, *K*_m_ = 60 µM.

*Bovine liver* β-galactosidase: 50 mM sodium phosphate buffer (pH 7). Substrate: *p*NP β-Gal, *K*_m_ = 0.65 mM.

Fabrazyme (Acid α-galactosidase): 20 mM sodium citrate, 50 mM sodium phosphate, 1.0 mM tetrasodium EDTA, 0.25% *v*/*v* Triton X-100^®^ and 0.25% *w*/*v* taurocholic acid buffer (pH 5.5). Substrate: 2,4-DNP α-Gal, *K*_m_ = 0.65 mM.

*S. cerevisiae* α-Glucosidase: 50 mM sodium phosphate buffer (pH 7.0). Substrate: *p*NP α-Glc, *K*_m_ = 0.75 mM.

GCase (β-glucocerebrosidase): 20 mM sodium citrate, 50 mM sodium phosphate, 1.0 mM tetrasodium EDTA, 0.25% *v*/*v* Triton X-100^®^ and 0.25% *w*/*v* taurocholic acid buffer (pH 7). Substrate: 2,4-DNP β-Glc, *K*_m_ = 2.7 mM.

## 4. Conclusions

We have developed a robust and flexible conceptual synthetic protocol towards *N*-alkylated iminosugar based inhibitors for glycoside hydrolases and have probed this concept on D-*gluco* (**45**, **47**, **49**, **51**, **53**, **55**) and d-*xylo* (**46**, **48**, **50**, **52**, **54**, **56**) configured iminosugar scaffolds. The sequence of the composition of the different building blocks allows for flexibility in choosing the spacer length and terminal tag on the non iminosugar hemisphere of the compound. Furthermore, we introduced different terminal tags such as nitrile, azide, alkyne, nonafluoro-*tert*-butyl and amino substituents, which allows for simple follow-up chemistry customised for different applications such as orthogonal labelling with fluorescent dyes as reporter groups or ligation reactions. The biological evaluation with a set of different glycoside hydrolases showed that all synthesised compounds proved to bind tightly to GCase with *K*_i_ values in the low micro and nanomolar range. Most of them exhibit also good selectivities, thereby clearly underlining the potential of this compound class to be used as tools and therapeutics in the context of human lysosomal β-glucocerebrosidase. Potential applications are their use as enzyme inhibitors, pharmacological chaperons and active site directed ligands for enzyme labelling.

## Figures and Tables

**Table 1:** *K*_i_-values [µM] of compounds **45**–**56** with Abg = β-glucosidase/β-galactosidase from *Agrobacterium* sp.; *E. coli* = *lac Z* β-galactosidase from *E. coli*; Bovine liv. = β-galactosidase from bovine liver; Fabrazyme = commercial recombinant human lysosomal α-galactosidase; *S. cer.* = α-glucosidase from *Saccharomyces cerevisiae*; GCase = recombinant human lysosomal β-glucocerebrosidase; N.I. = no or weak inhibition, with *K*_i_ > 1 mM;

Compound					Enzyme (GH Family)					
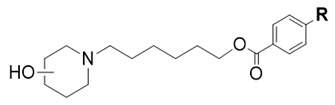										
	
			β-Glc/Gal		β-Gal	β-Gal	α-Gal	α-Glc	β-Glc	
R =	*Config.*	Nr.	Abg (GH1)		*E*. *coli* (GH2)	Bovine liv. (GH35)	Fabrazyme (GH27)	*S. cer.* (GH13)	GCase (GH30)	
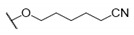	***Glc***	**45**	1.31		N.I.	2.42	N.I.	23.7	0.28	
	***Xyl***	**46**	12.10		N.I.	7.76	N.I.	N.I.	0.62	
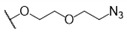	***Glc***	**47**	1.70		N.I.	1.74	N.I.	221	0.09	
	***Xyl***	**48**	32.6		N.I.	11.76	N.I.	N.I.	1.40	
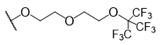	***Glc***	**49**	3.60		N.I.	6.94	N.I.	N.I.	0.35	
	***Xyl***	**50**	23.70		255	8.36	N.I.	N.I.	0.40	
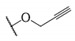	***Glc***	**51**	0.0022		N.I.	3.25	N.I.	4.9	0.37	
	***Xyl***	**52**	17		N.I.	7.41	N.I.	267	0.74	
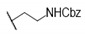	***Glc***	**53**	0.06		424	2.13	N.I.	8.1	0.022	
	***Xyl***	**54**	0.58		N.I.	4.65	N.I.	N.I.	0.31	
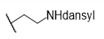	***Glc***	**55**	0.06		89	1.82	N.I.	8.7	0.018	
	***Xyl***	**56**	0.76		198	10.36	N.I.	N.I.	2.60	

## Supplementary Materials

The following are available online, NMR spectra for new compounds. Figure S1: Structure of compound 35 confirmed by XRD analysis (CCDC 2021385).
