# Supplementary material for: N-Alkylated Iminosugar Based Ligands: Synthesis and Inhibition of Human Lysosomal β-Glucocerebrosidase
Source: Molecules. 2020 Oct 11;25(20):4618. doi: 10.3390/molecules25204618 (PMC7594070; doi:10.3390/molecules25204618)
Supplement: Supplementary file 1 [file molecules-25-04618-s001.pdf]

Supplementary Materials:

N-Alkylated Iminosugar Based Ligands: Synthesis and Inhibition of Human Lysosomal β-Glucocerebrosidase

Andreas Wolfsgruber [1], Martin Thonhofer [1], Patrick Weber [1], Seyed A. Nasseri [2], Roland Fischer [3], Michael Schalli [1], Arnold E. Stütz [1], Stephen G. Withers [2] and Tanja M. Wrodnigg [1,*]

[1] Glycogroup, Institute of Chemistry and Technology of Biobased Systems, Graz University of Technology, Stremayrgasse 9, A-8010 Graz, Austria; andreas.wolfsgruber@tugraz.at (A.W.); thonhofer@tugraz.at (M.T.); patrick.weber@tugraz.at (P.W.); michael.schalli@medunigraz.at (M.S.); stuetz@tugraz.at (A.E.S.);

[2] Chemistry Department, University of British Columbia, 2036 Main Mall, Vancouver, BC V6T 1Z1, Canada; withers@chem.ubc.ca (S.G.W.); snasseri@chem.ubc.ca (S.A.N.)

[3] Institute of Inorganic Chemistry, Graz University of Technology, Stremayrgasse 9, A-8010 Graz, Austria; roland.fischer@tugraz.at

* Correspondence: t.wrodnigg@tugraz.at; Tel.: +43-316-873-32073

Academic Editor: László Somsák
Received: 8 September 2020; Accepted: 25 September 2020; Published: 11 October 2020

Supplementary data: ^{1}H-NMR, ^{13}C-NMR, ^{13}C-APT NMR, ^{19}F-NMR, HSQC, COSY spectra and XRD data

1) NMR spectra of new compounds:

Methyl 4-((5-cyanopentyl)oxy)benzoate (22)

^{1}H-NMR (300 MHz, $CDCl_3$): Compound **22**

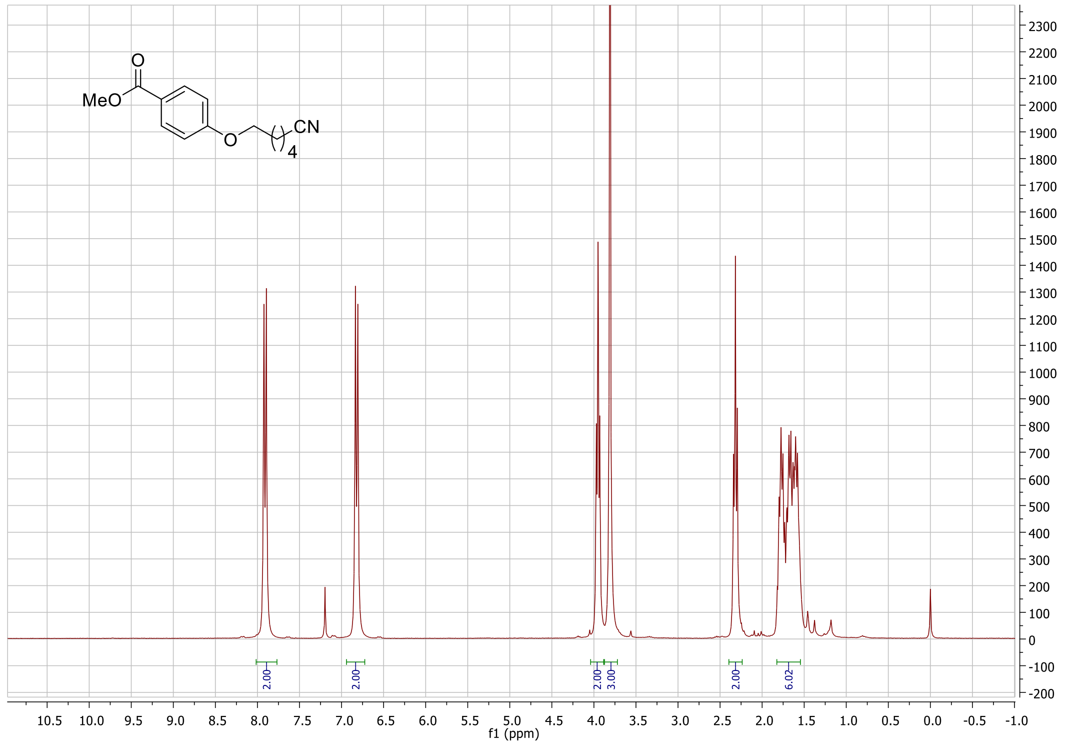

^{13}C-NMR (75.5 MHz, $CDCl_3$): Compound **22**

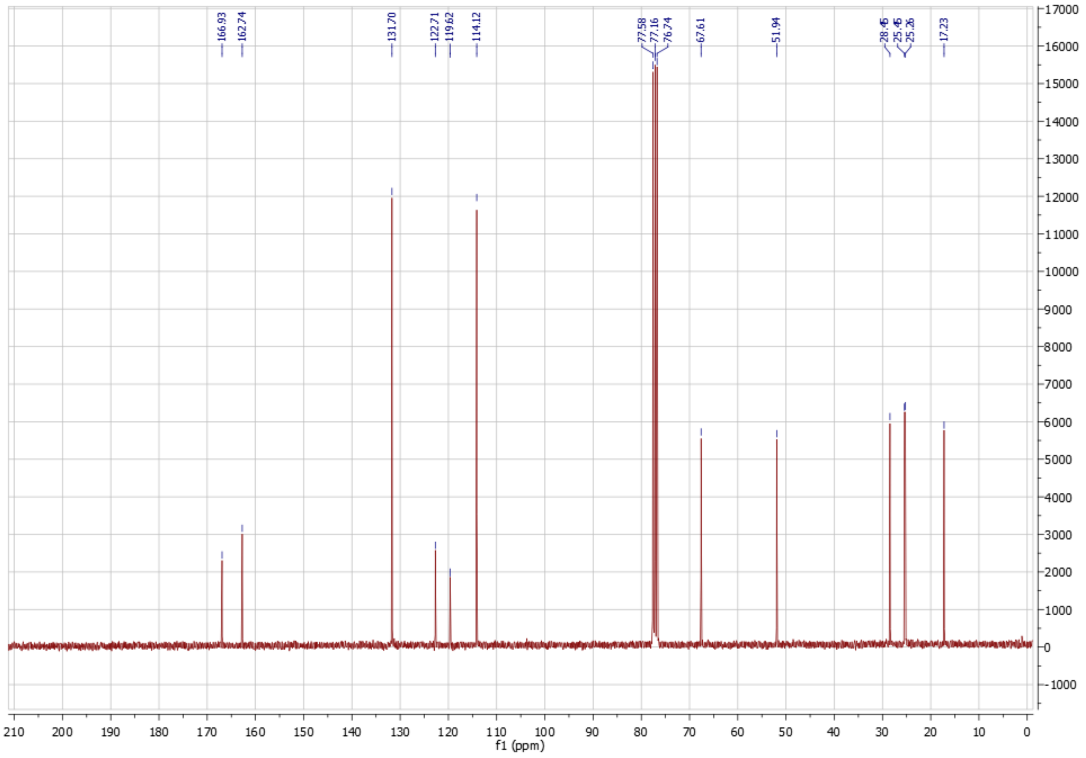

COSY (CDCl3): Compound **22**

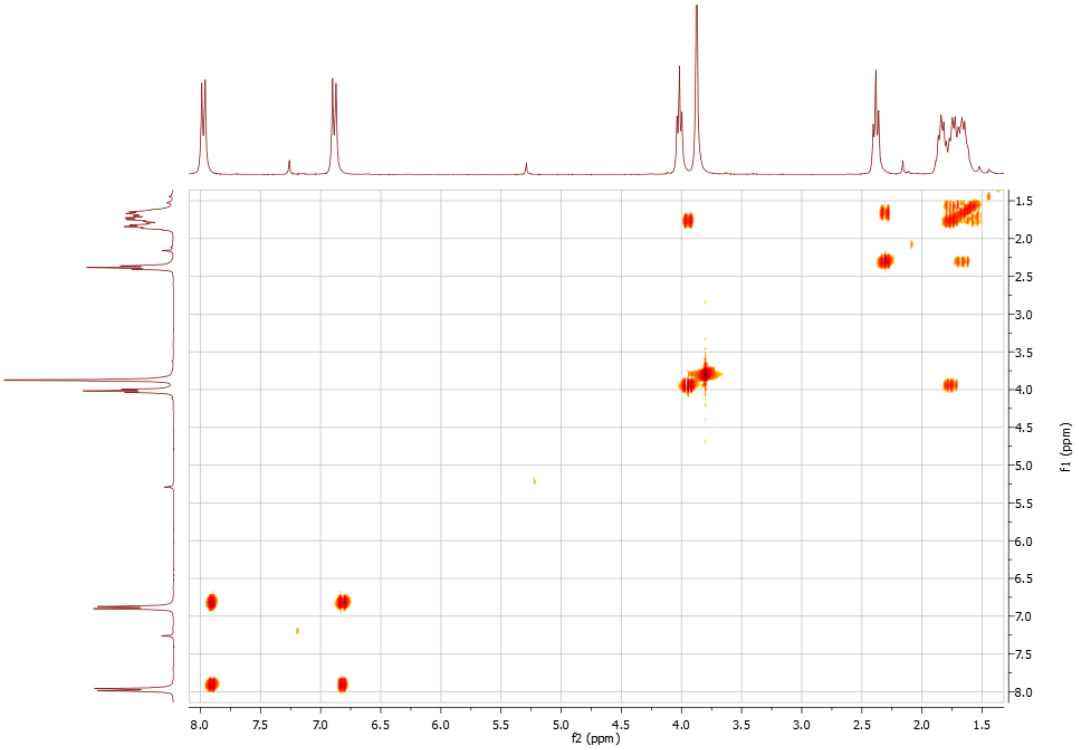

HSQC (CDCl3): Compound **22**

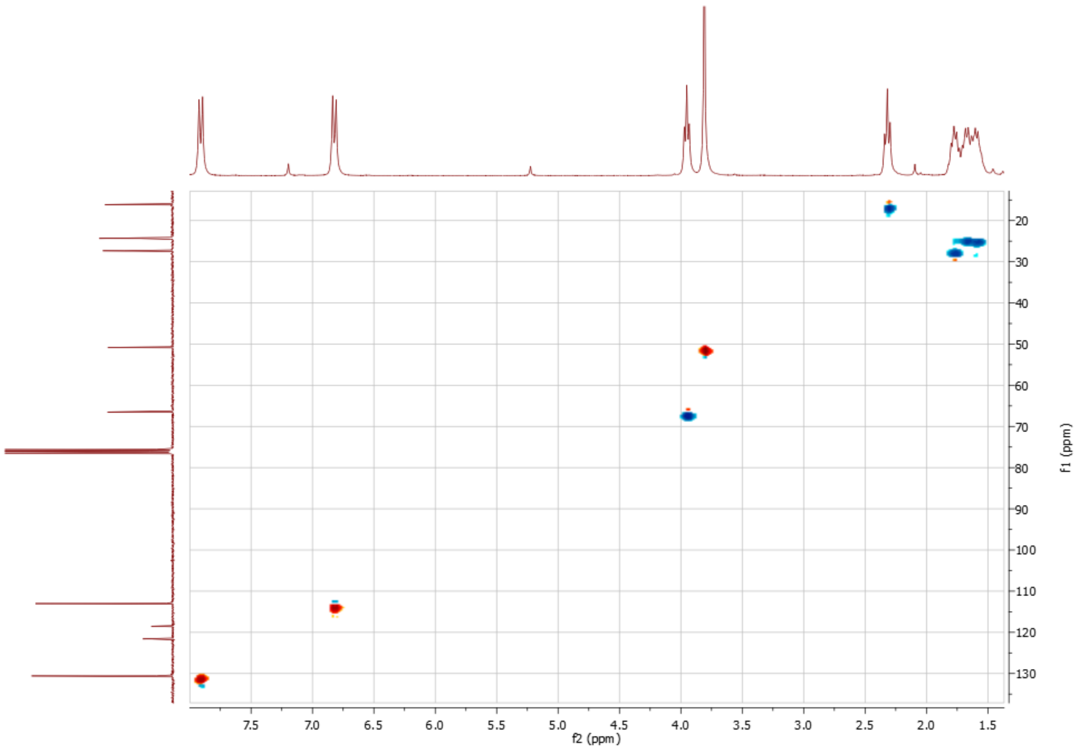

6-Chlorohexyl 4-((5-cyanopentyl)oxy)benzoate (24)

^{1}**H-NMR** (300 MHz, $CDCl_3$): Compound **24**

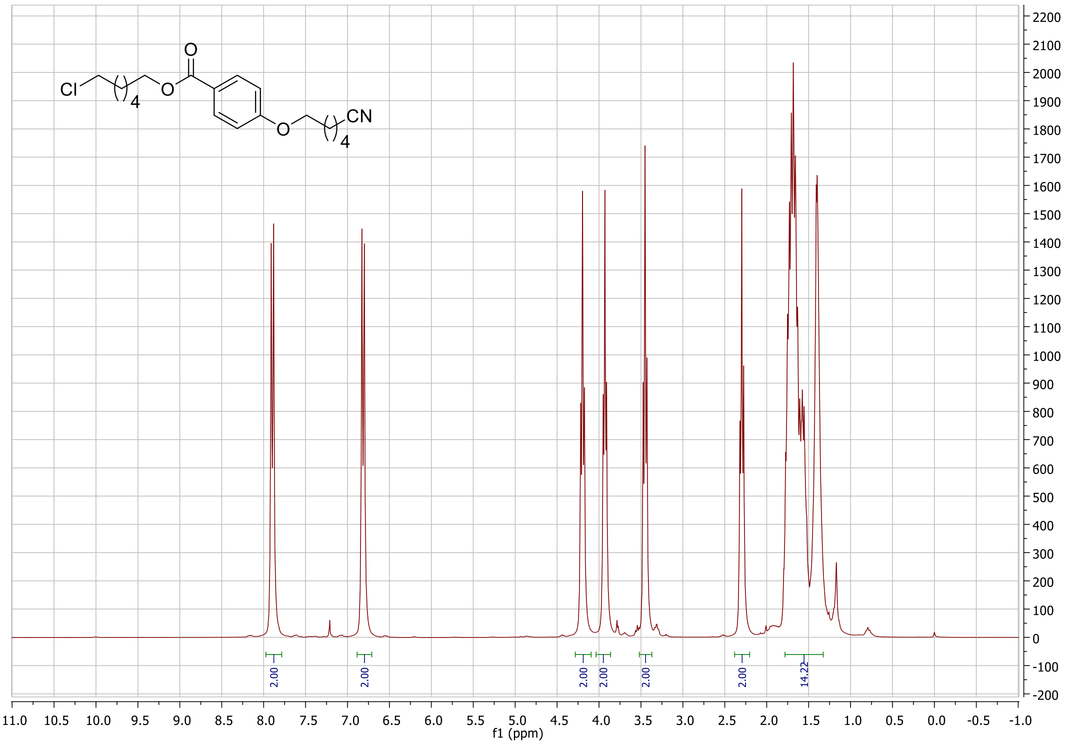

^{13}**C-APT NMR** (75.5 MHz, $CDCl_3$): Compound **24**

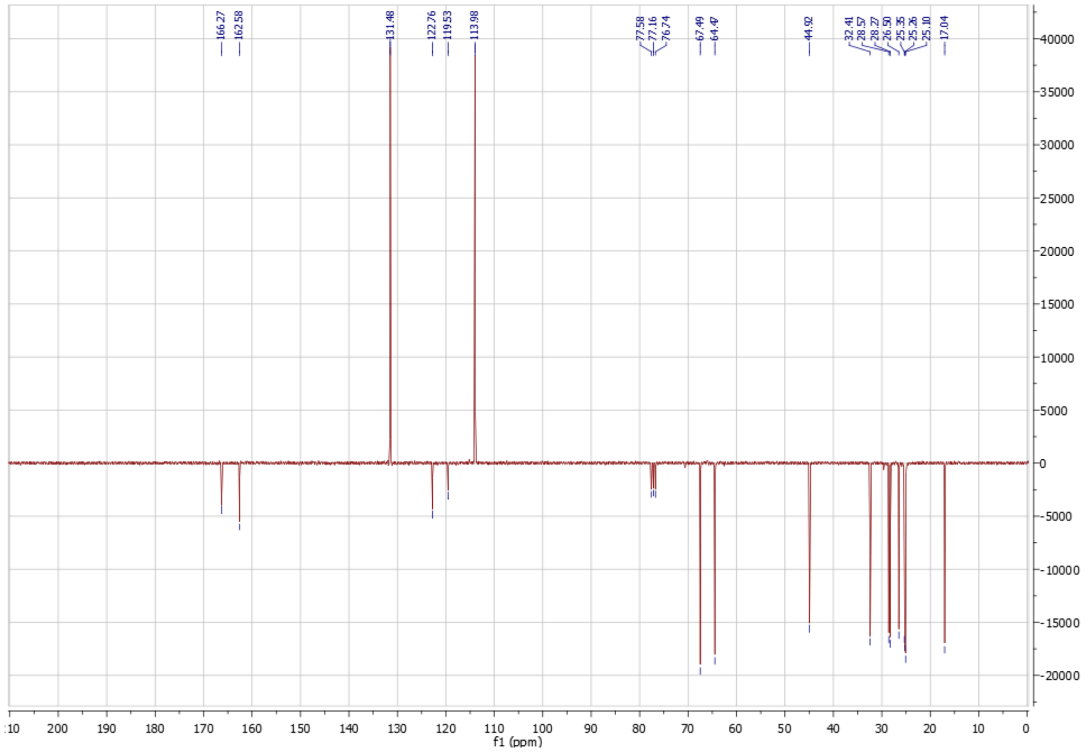

COSY ($CDCl_3$): Compound **24**

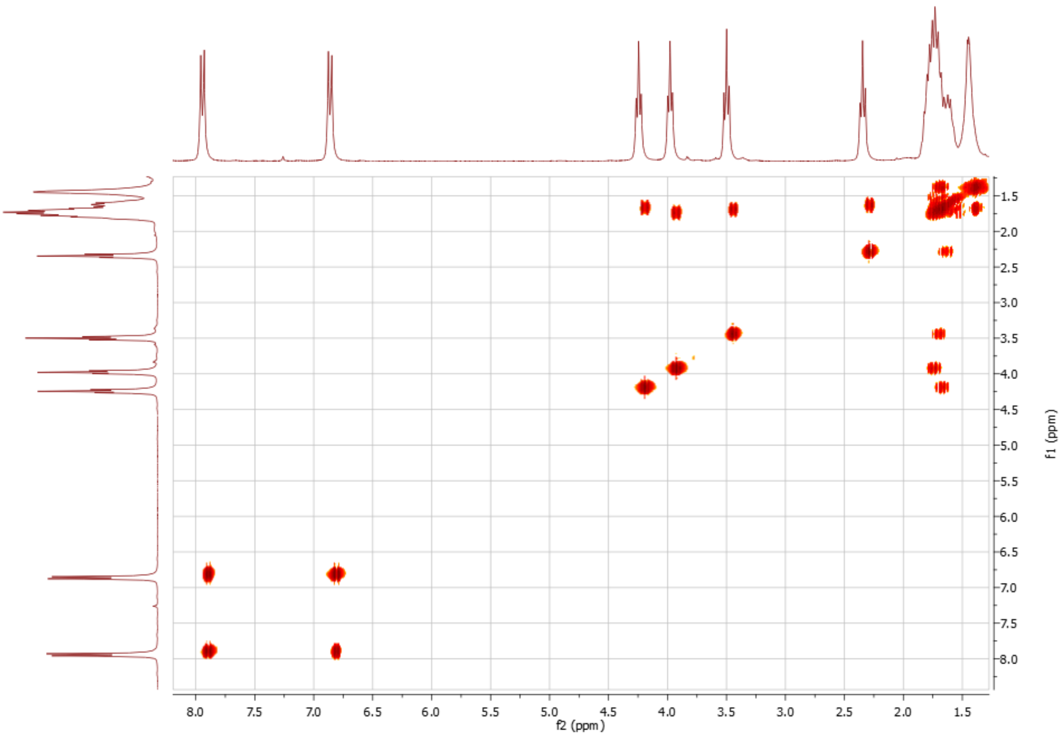

HSQC ($CDCl_3$): Compound **24**

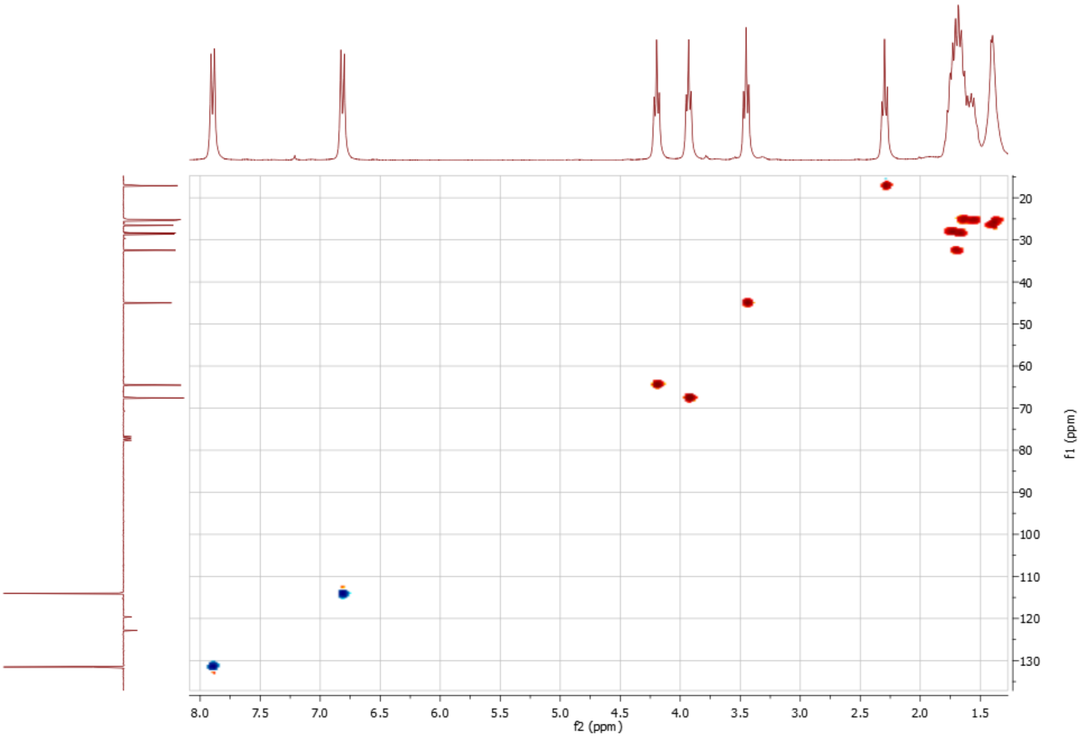

6-Oxohexyl-4-((5-cyanopentyl)oxy)benzoate (25)

^{1}H-NMR (300 MHz, $CDCl_3$): Compound **25**

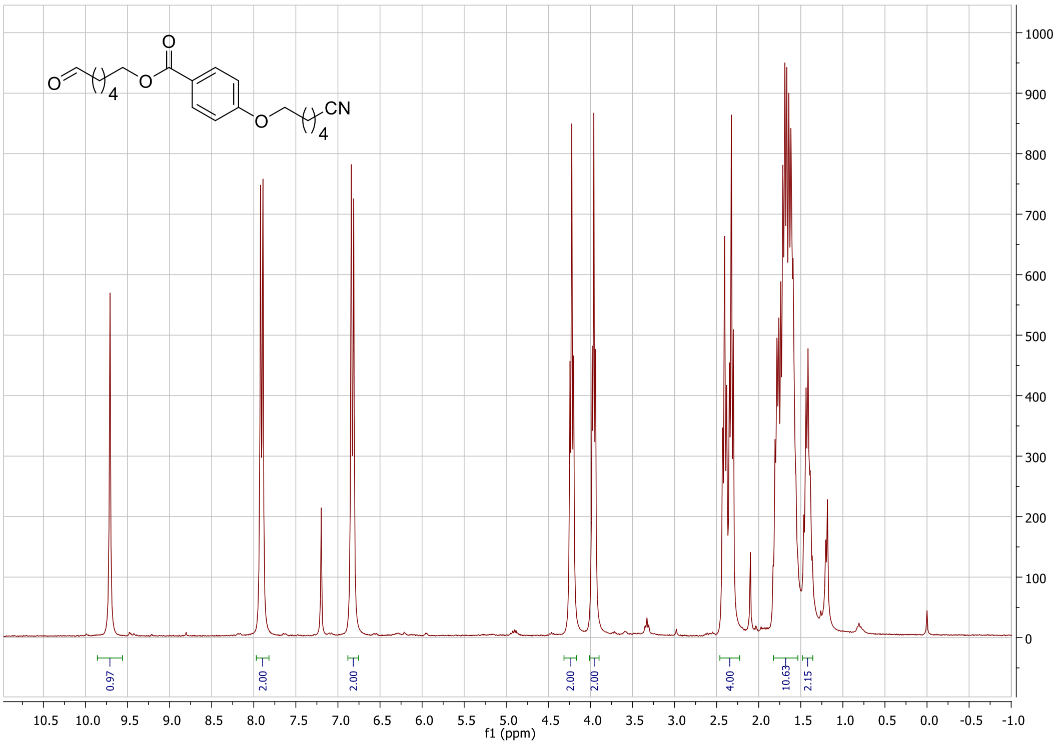

^{13}C NMR (75.5 MHz, $CDCl_3$): Compound **25**

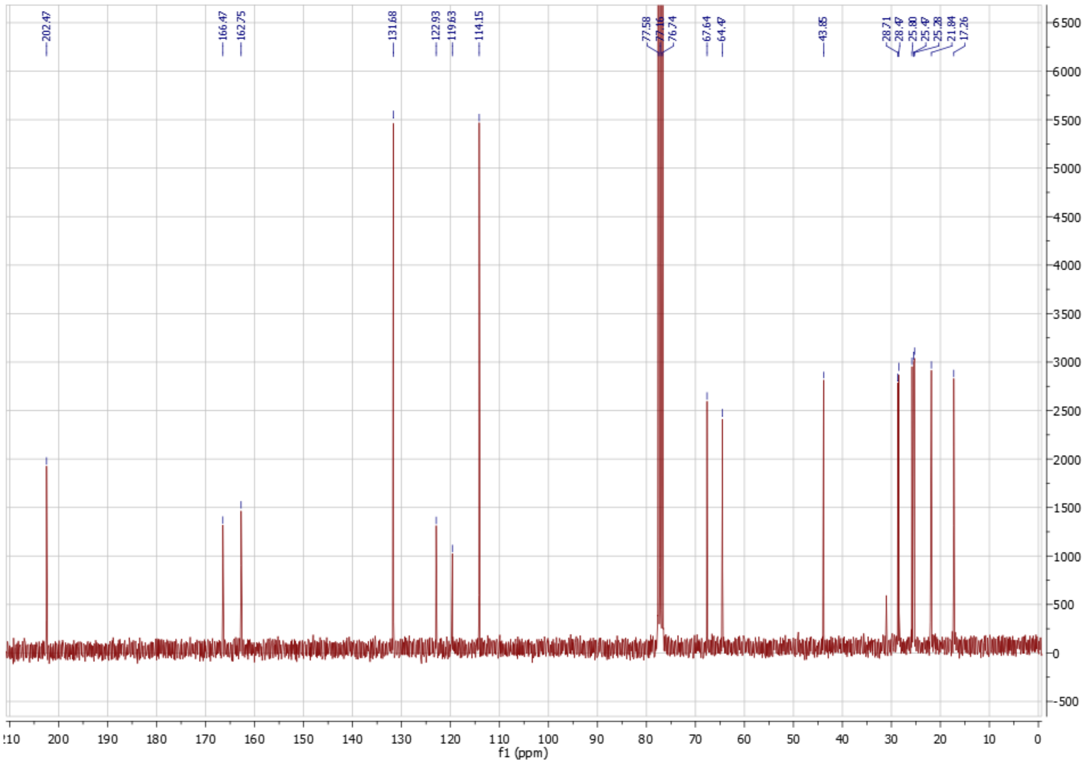

COSY ($CDCl_3$): Compound **25**

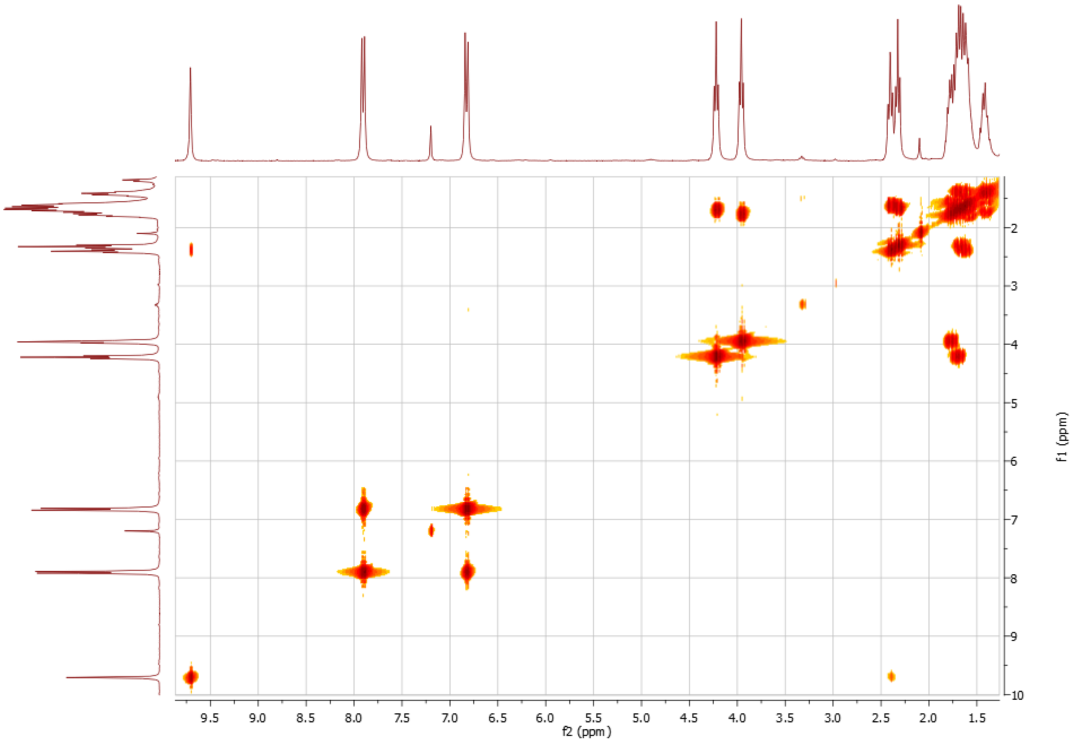

HSQC ($CDCl_3$): Compound **25**

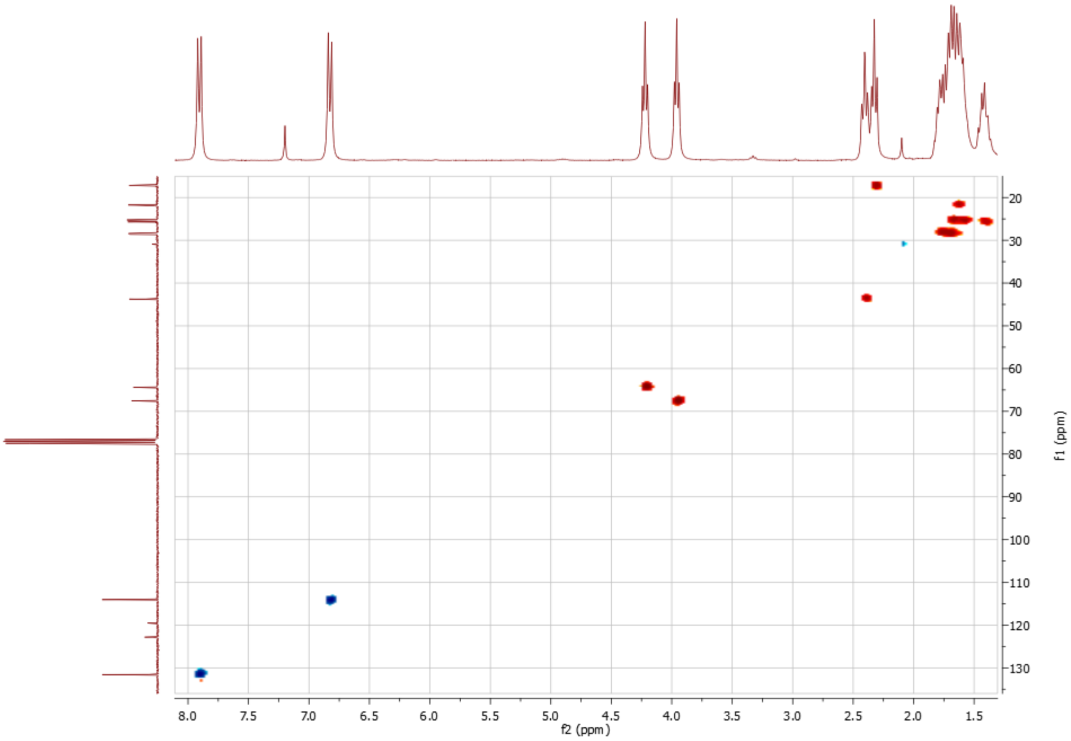

6-Bromohexyl 4-((tetrahydro-2*H*-pyran-2-yl)oxy)benzoate (27)

^{1}**H-NMR** (300 MHz, $CDCl_3$): Compound **27**

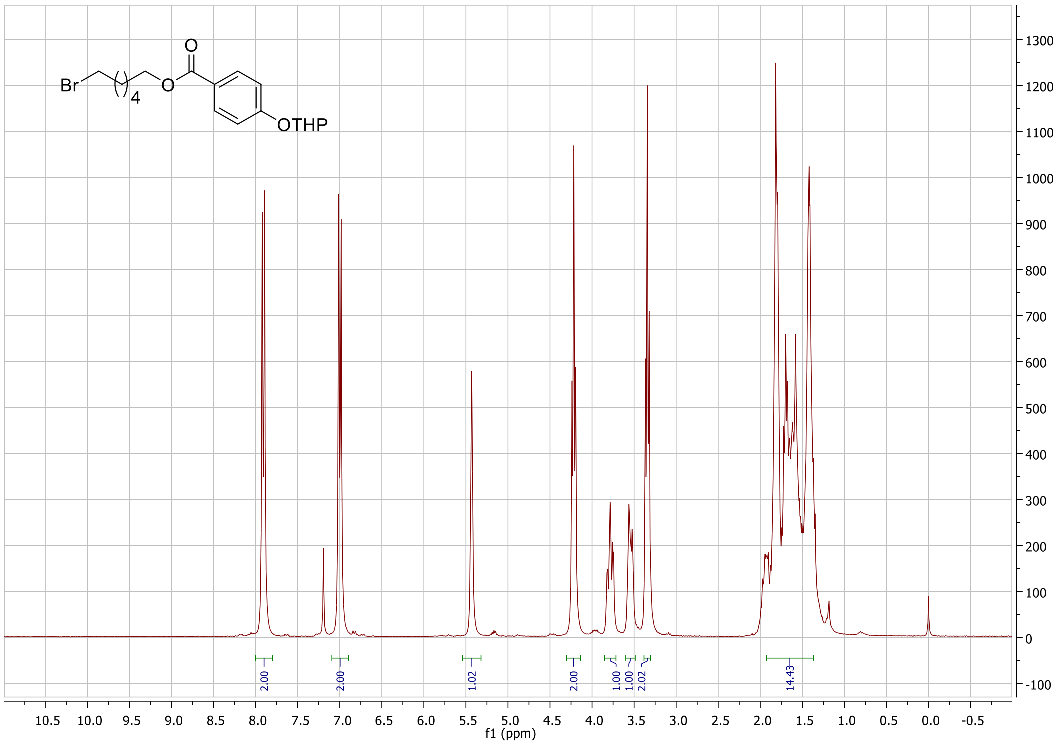

^{13}**C-NMR** (75.5 MHz, $CDCl_3$): Compound **27**

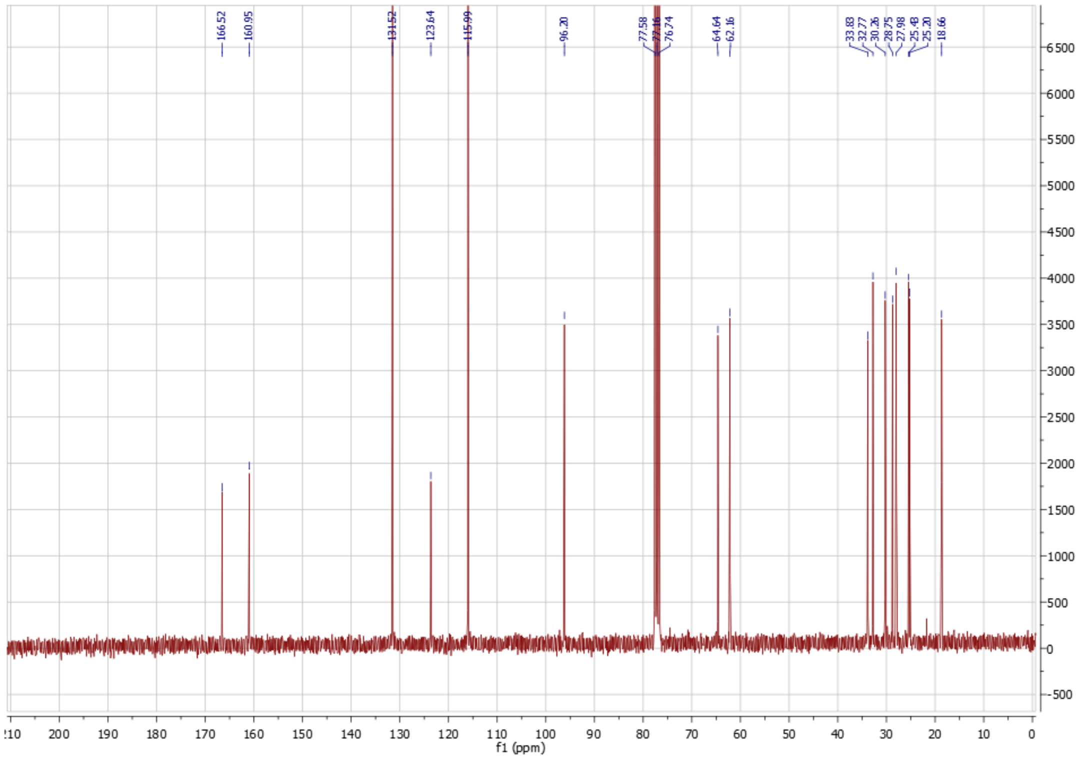

COSY ($CDCl_3$): Compound **27**

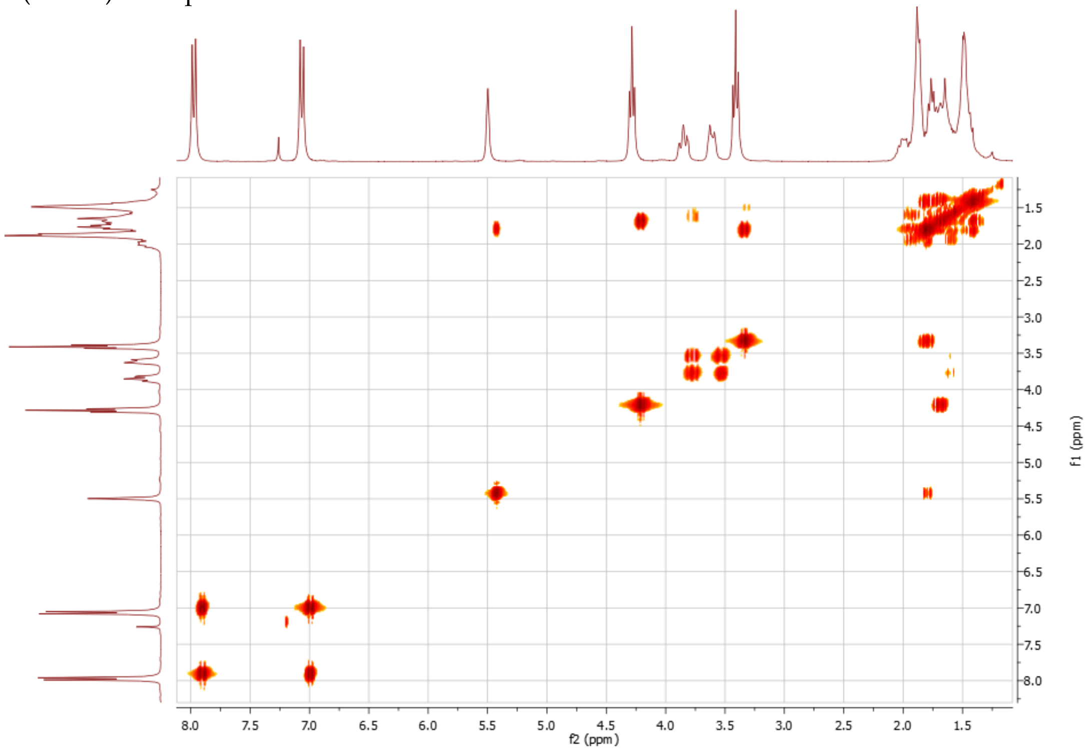

HSQC ($CDCl_3$): Compound **27**

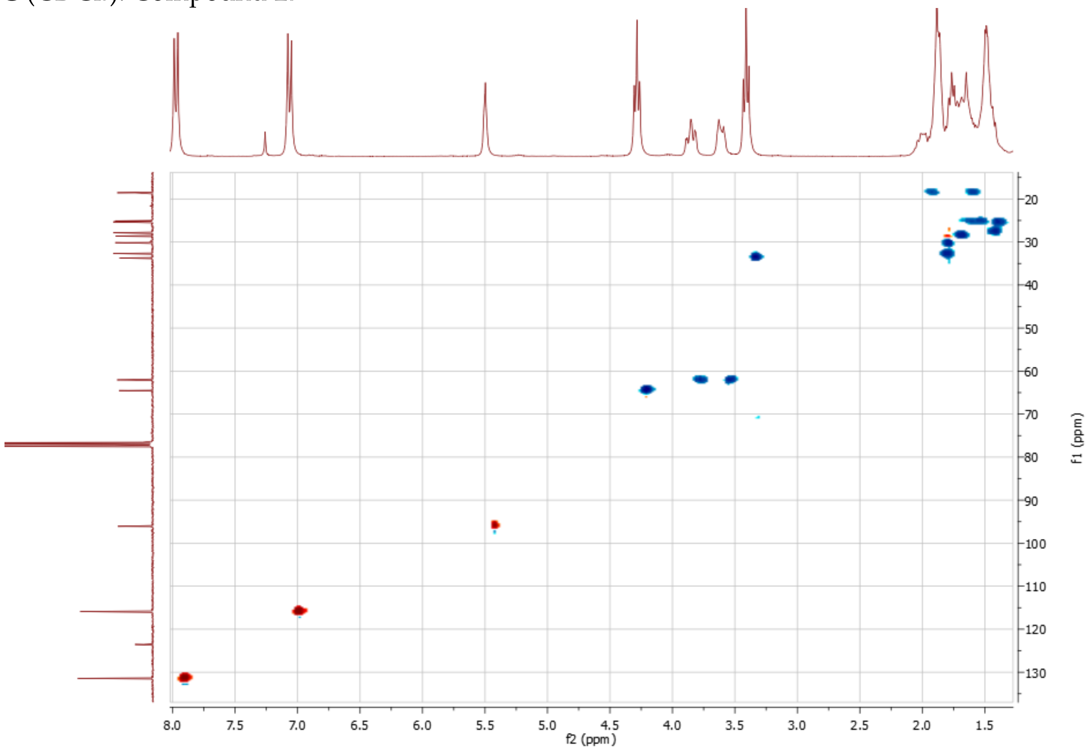

6-Bromohexyl 4-hydroxybenzoate (28)

[1]**H-NMR** (300 MHz, $CDCl_3$): Compound **28**

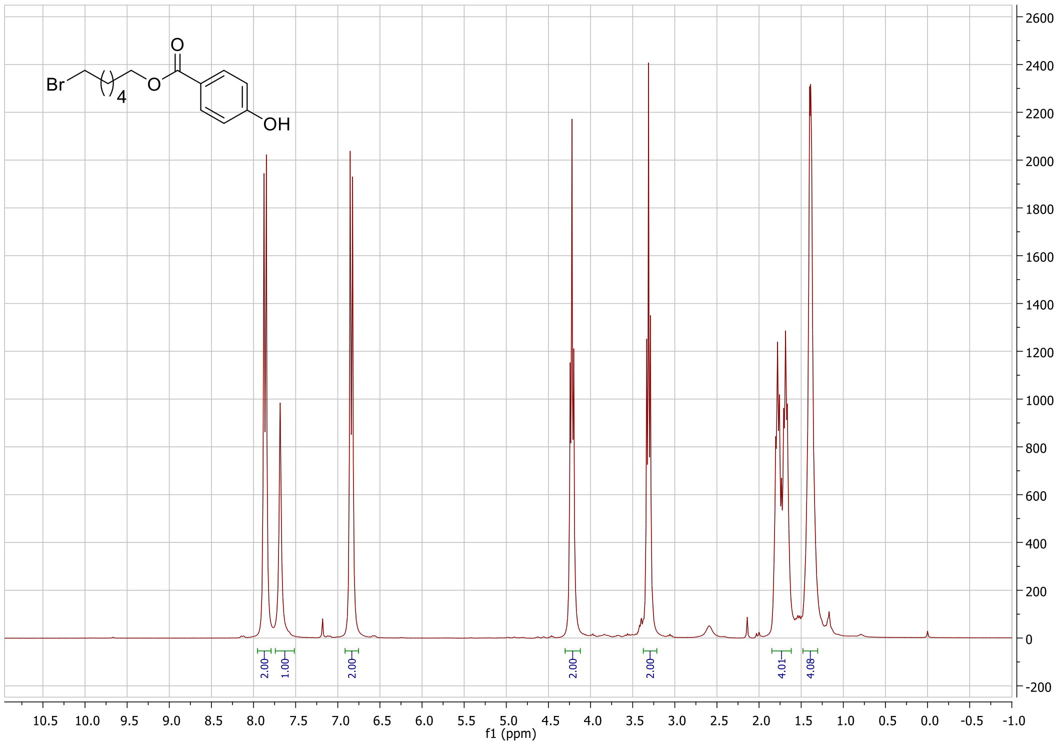

[13]**C-NMR** (75.5 MHz, $CDCl_3$): Compound **28**

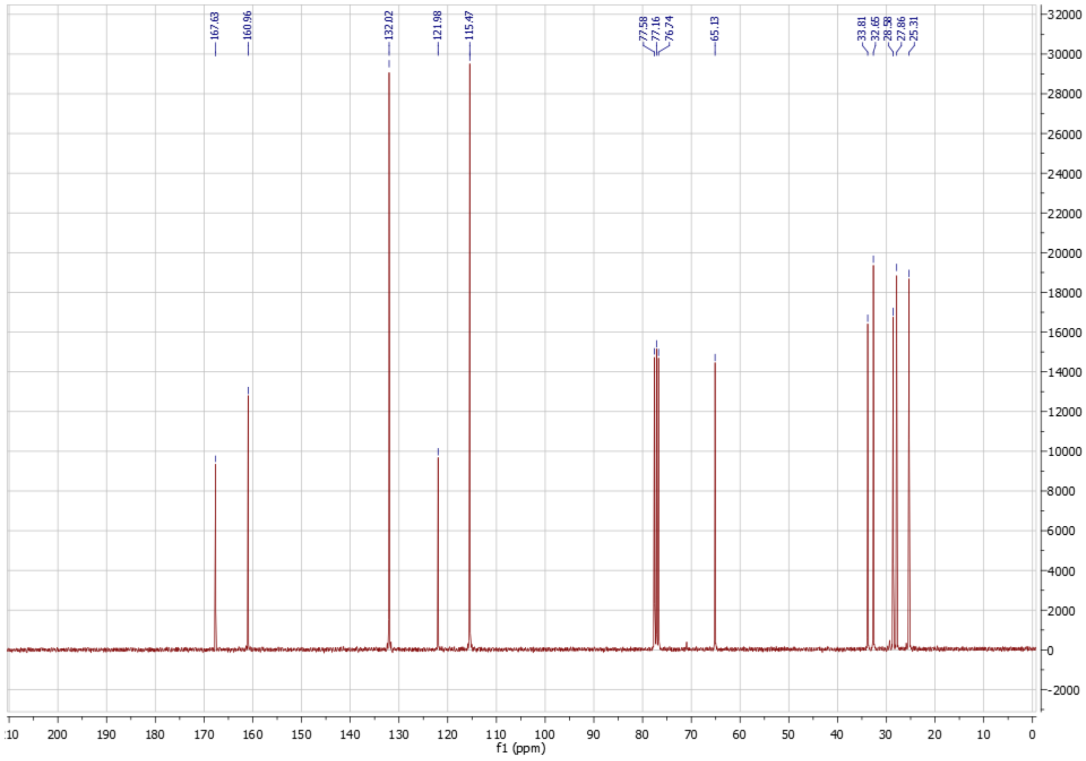

COSY (CDCl₃): Compound **28**

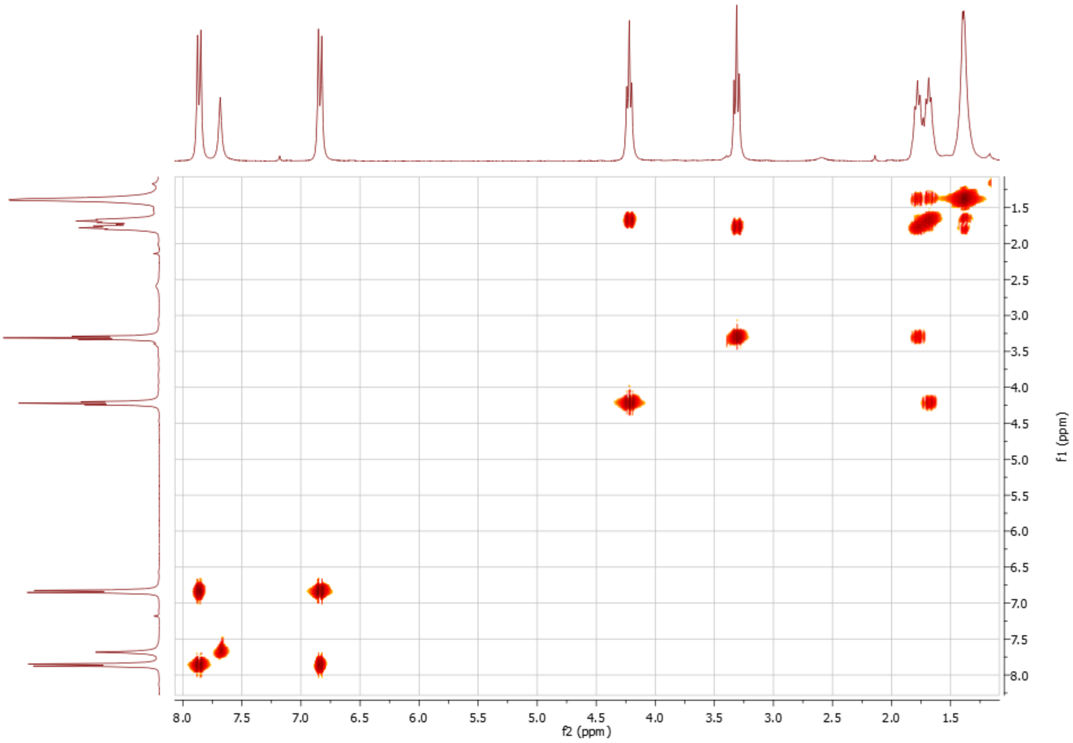

HSQC (CDCl₃): Compound **28**

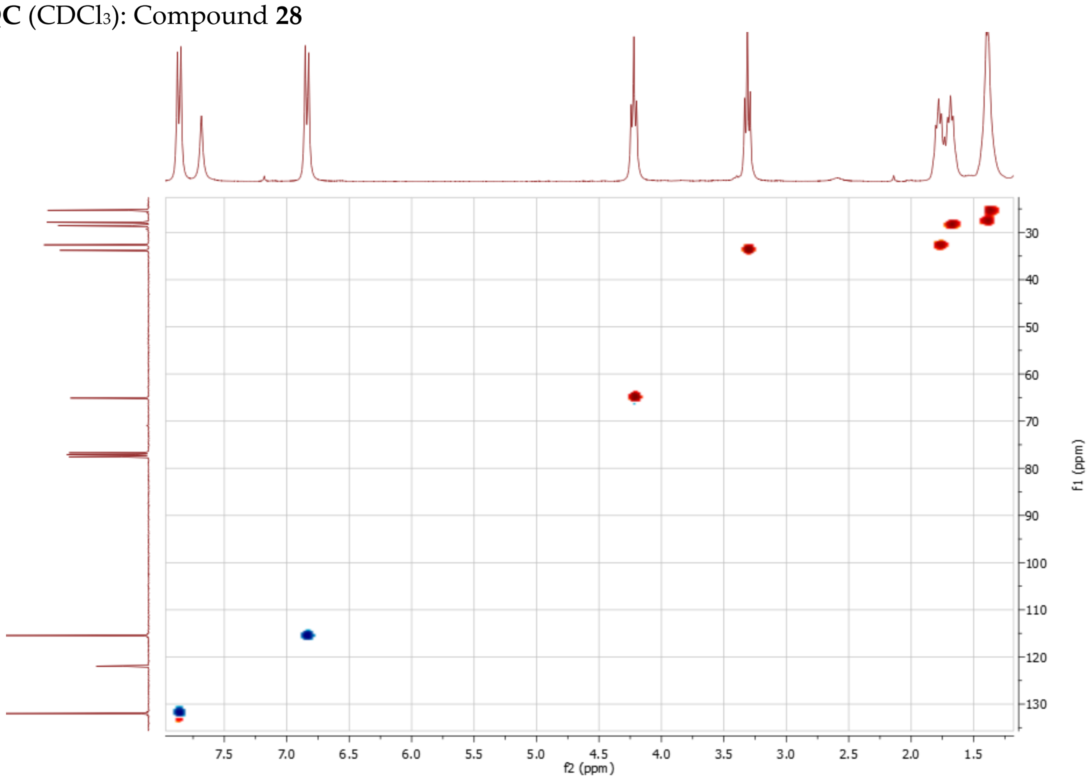

6-Bromohexyl 4-(2-(2-azidoethoxy)ethoxy)benzoate (29)

[1]**H-NMR** (300 MHz, $CDCl_3$): Compound **29**

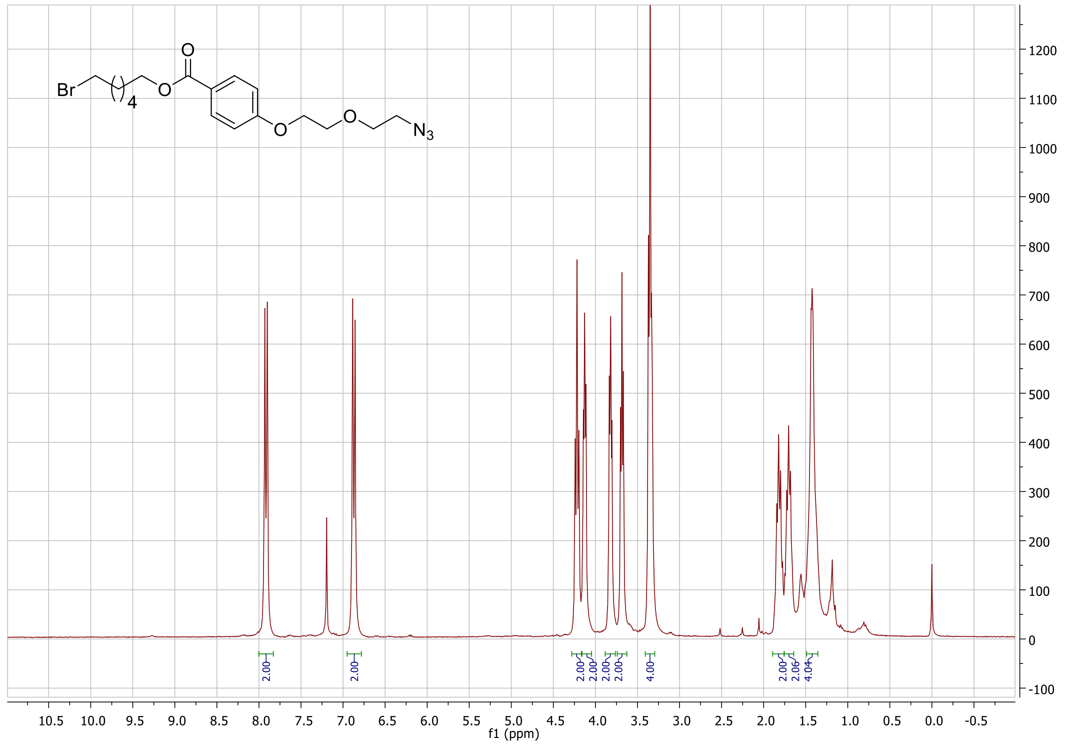

[13]**C-NMR** (75.5 MHz, $CDCl_3$): Compound **29**

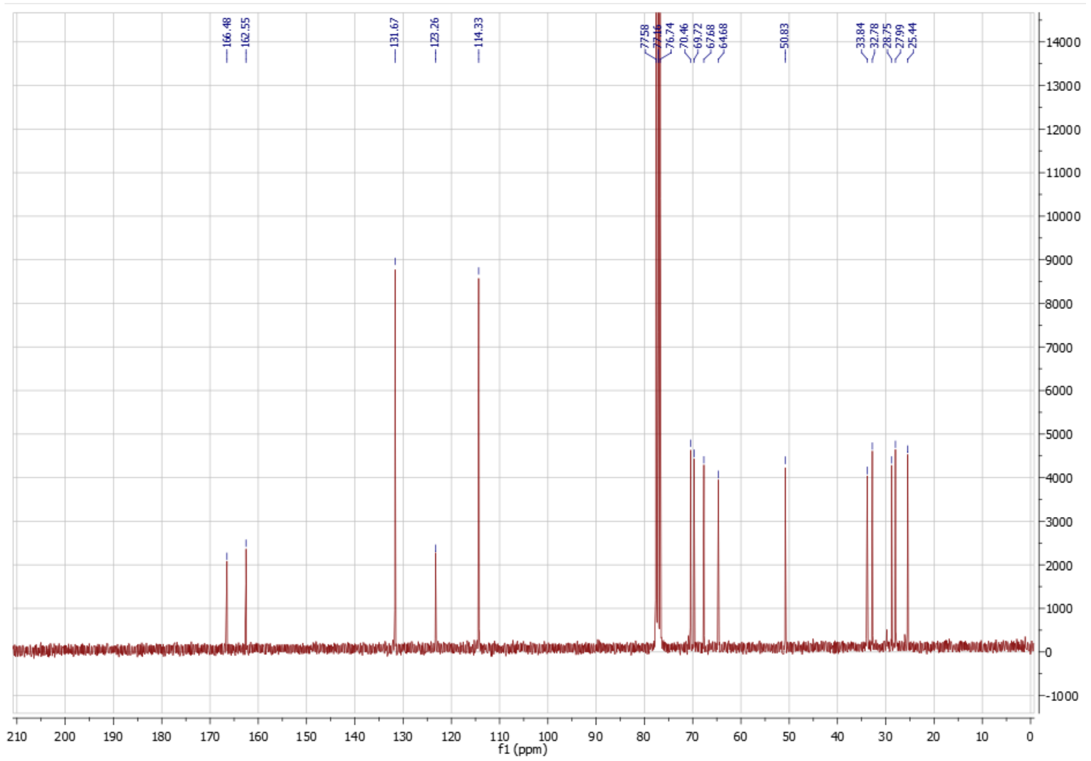

COSY (CDCl3): Compound **29**

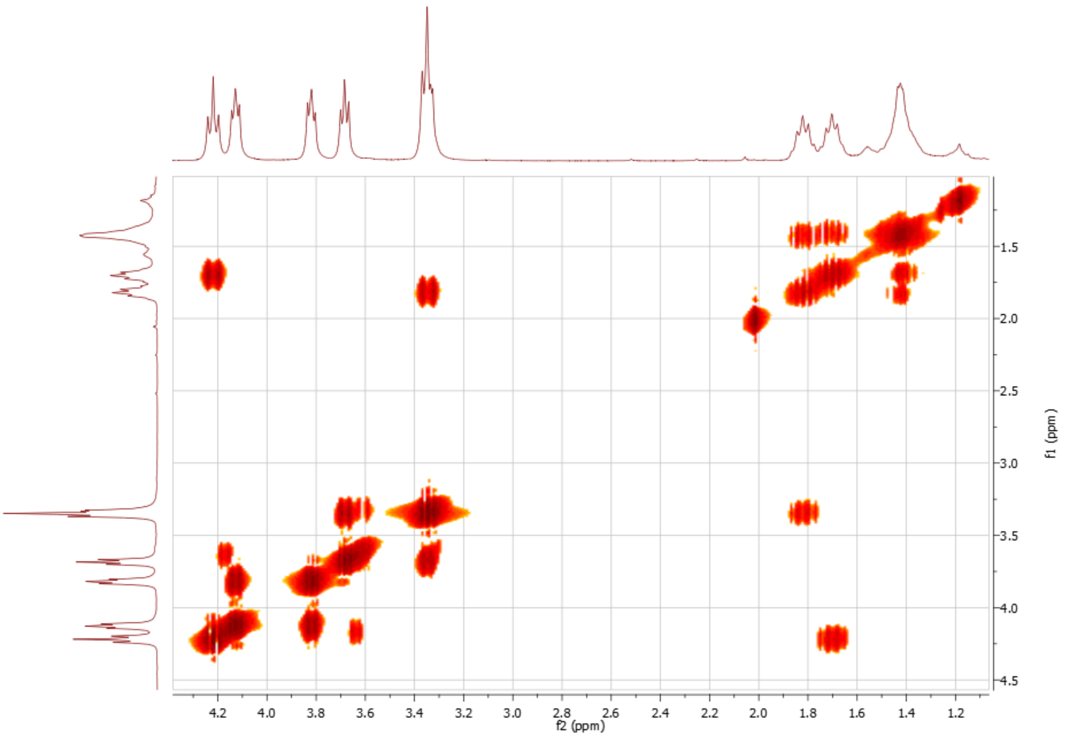

HSQC (CDCl3): Compound **29**

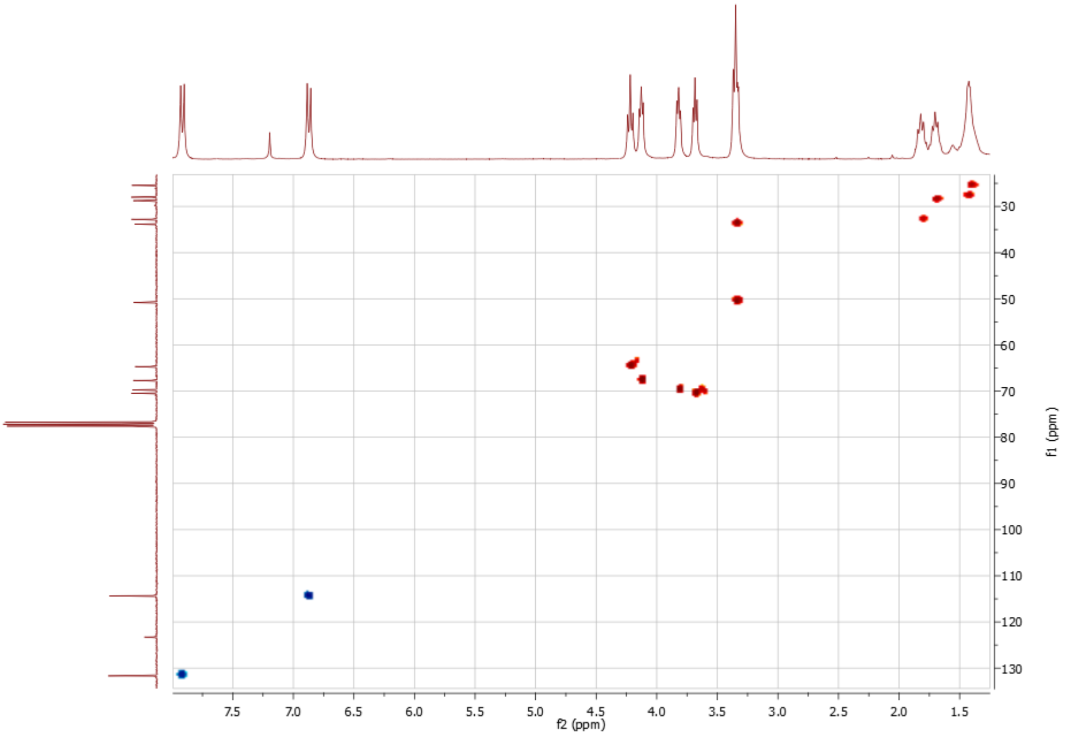

6-Oxohexyl 4-(2-(2-azidoethoxy)ethoxy)benzoate (30)

[1]**H-NMR** (300 MHz, CDCl3): Compound **30**

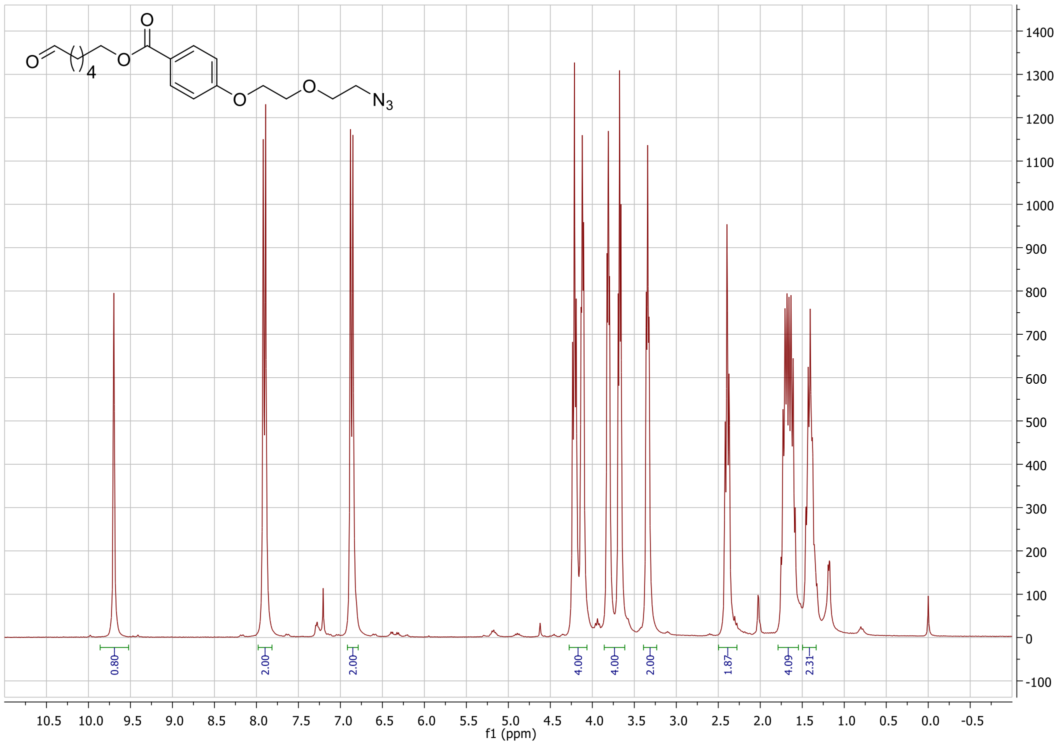

[13]**C-NMR** (75.5 MHz, CDCl3): Compound **30**

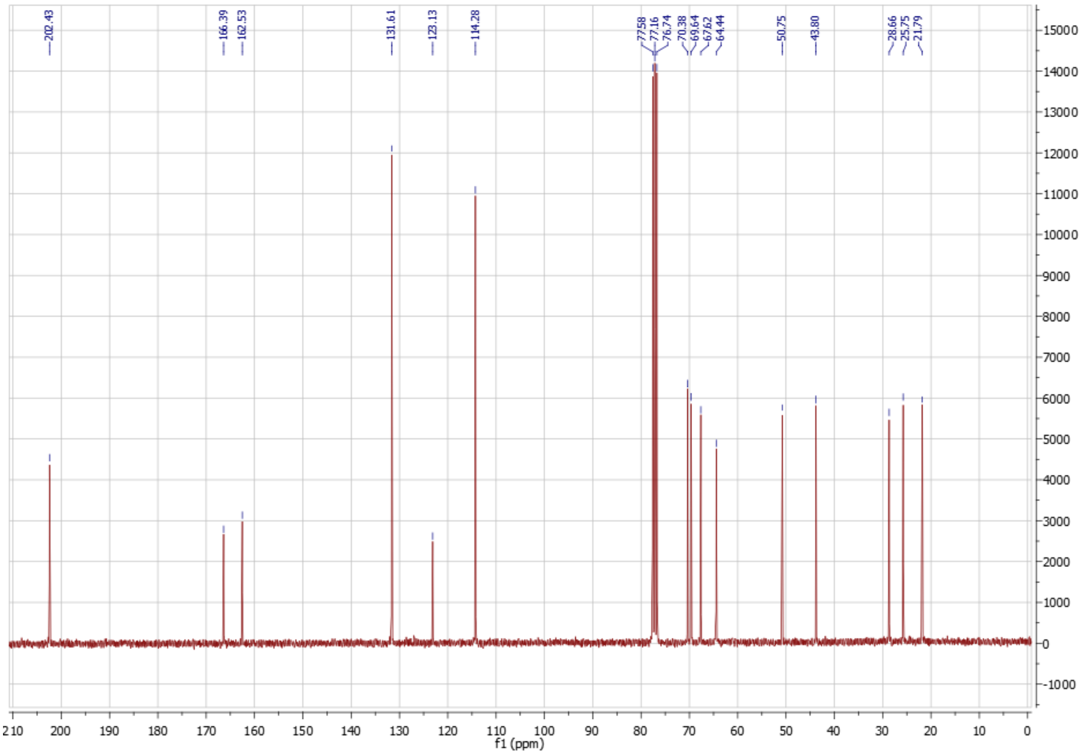

COSY ($CDCl_3$): Compound **30**

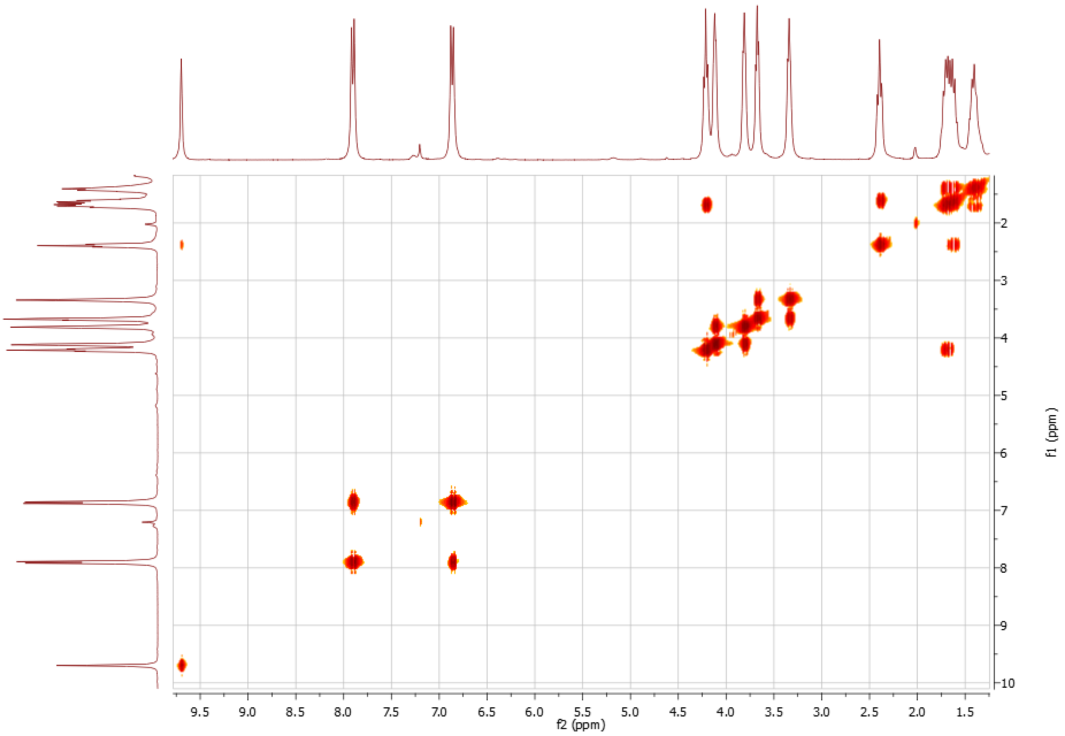

HSQC ($CDCl_3$): Compound **30**

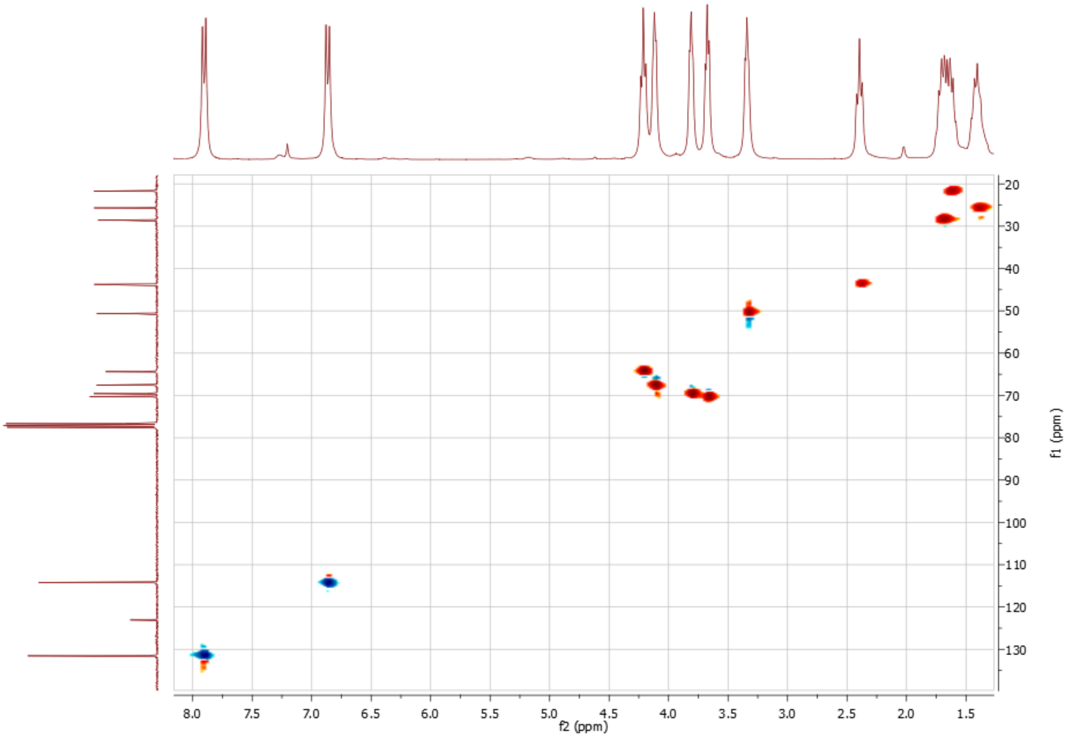

6-Chlorohexyl 4-((tetrahydro-2*H*-pyran-2-yl)oxy)benzoate (31)

^{1}H-NMR (300 MHz, CDCl$_3$): Compound **31**

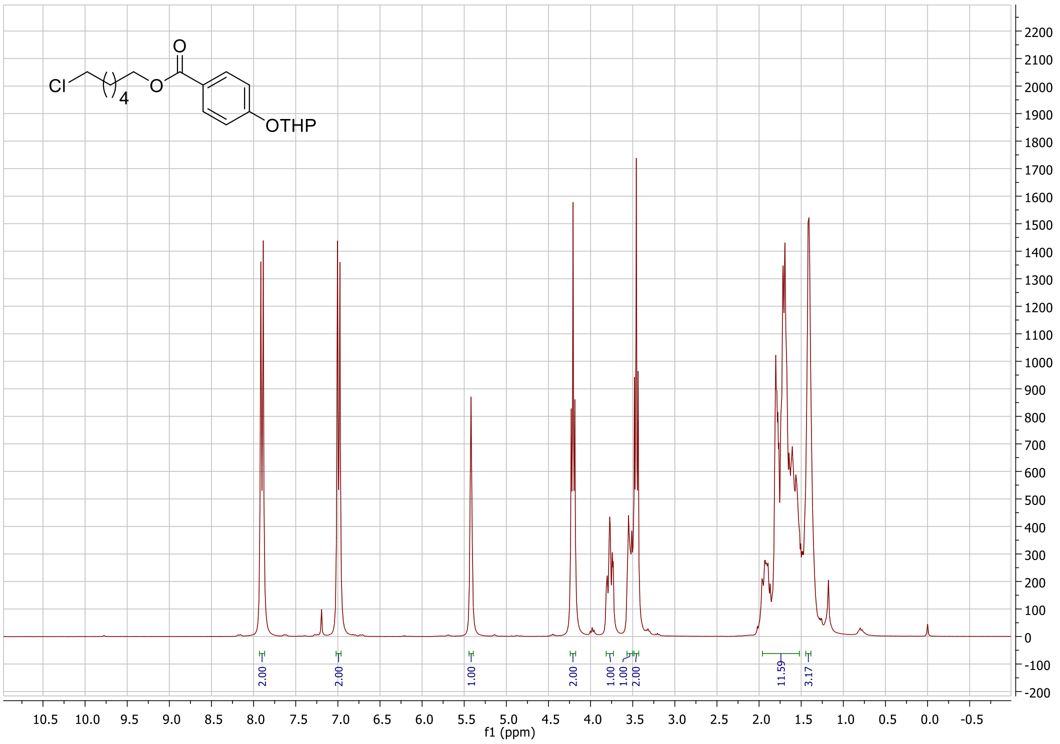

^{13}C-NMR (75.5 MHz, CDCl$_3$): Compound **31**

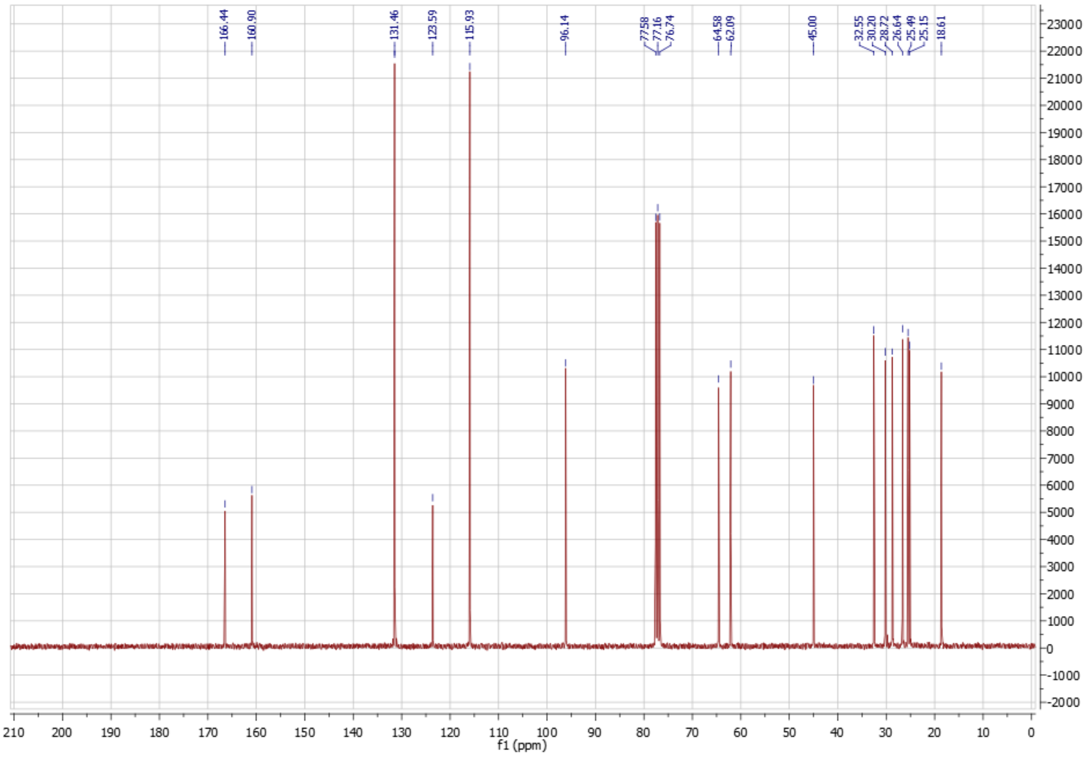

COSY (CDCl$_3$): Compound **31**

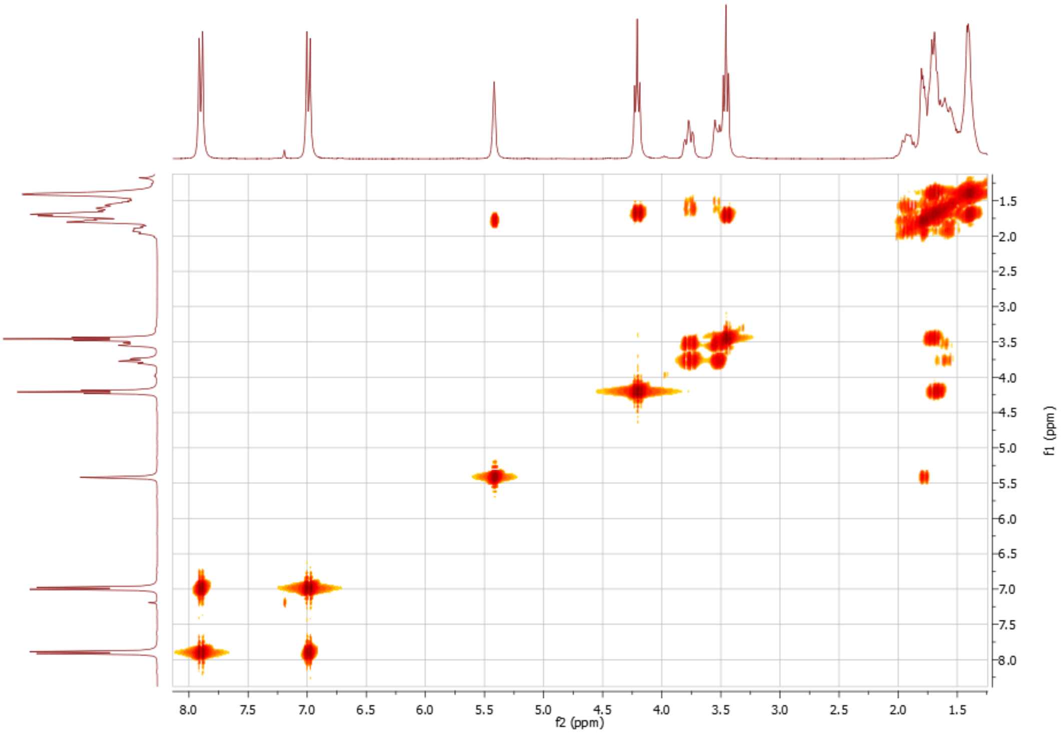

HSQC (CDCl$_3$): Compound **31**

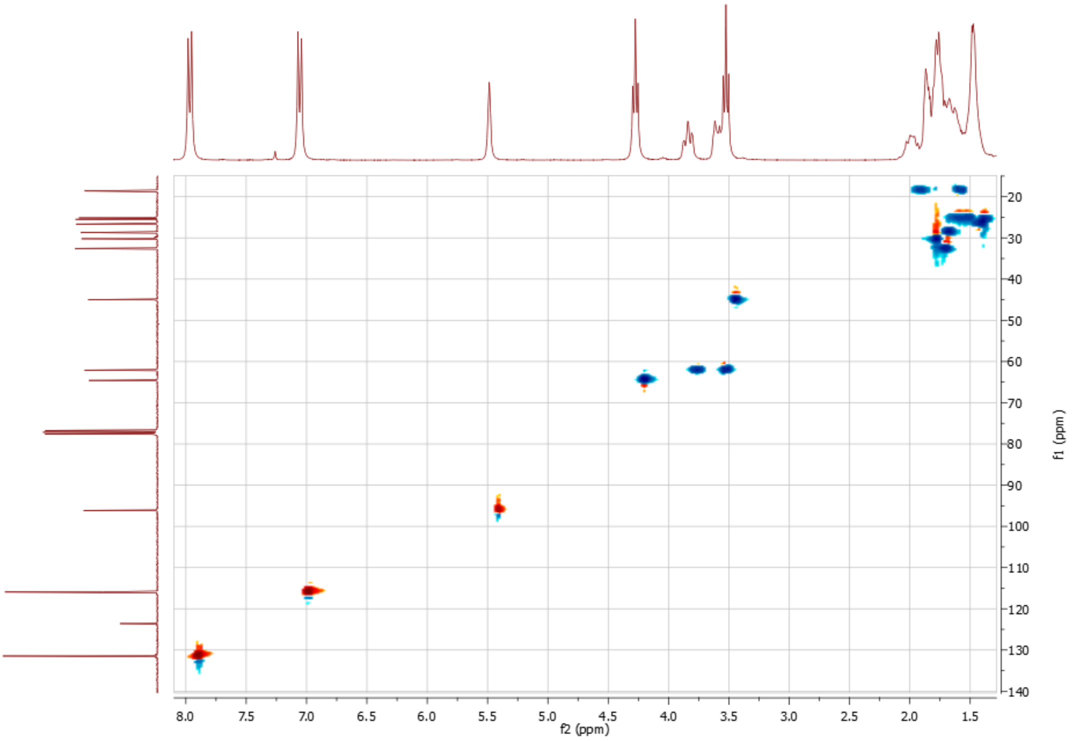

6-Chlorohexyl 4-hydroxybenzoate (32)

^{1}**H-NMR** (300 MHz, $CDCl_3$): Compound **32**

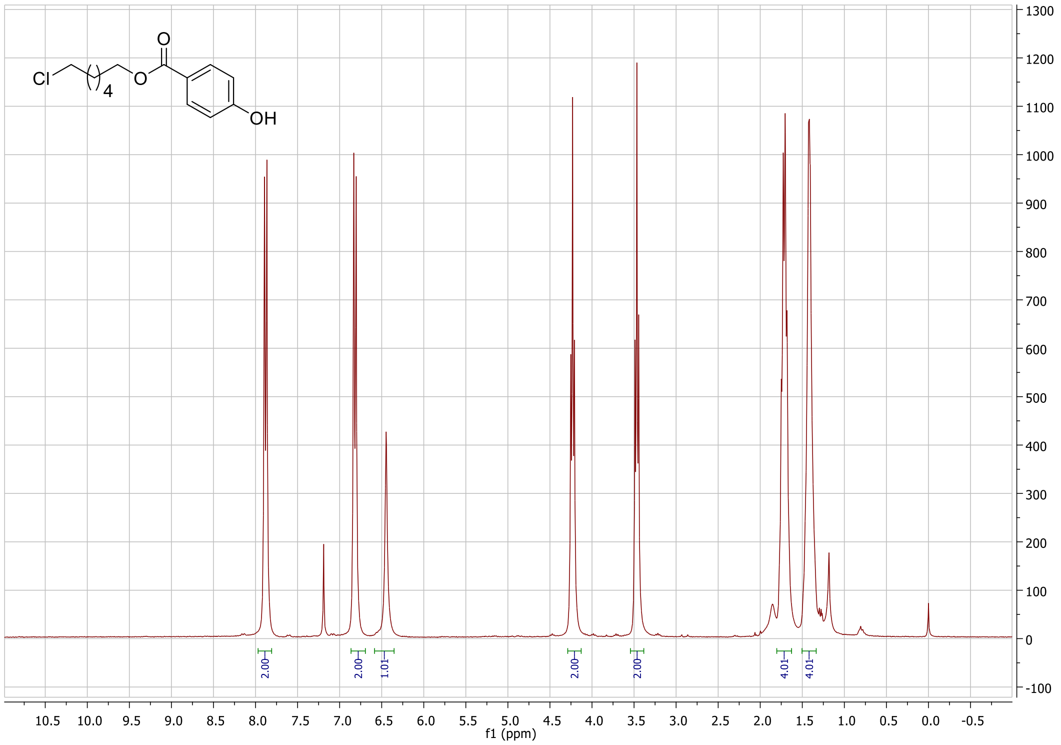

^{13}**C-NMR** (75.5 MHz, $CDCl_3$): Compound **32**

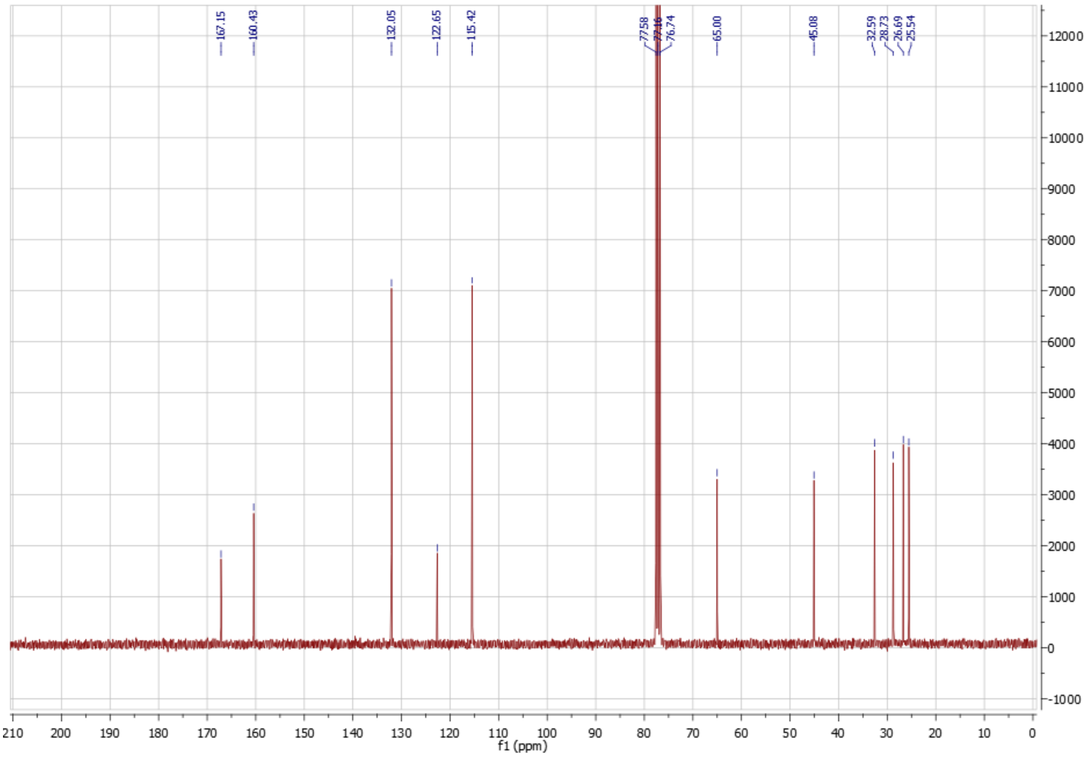

COSY ($CDCl_3$): Compound **32**

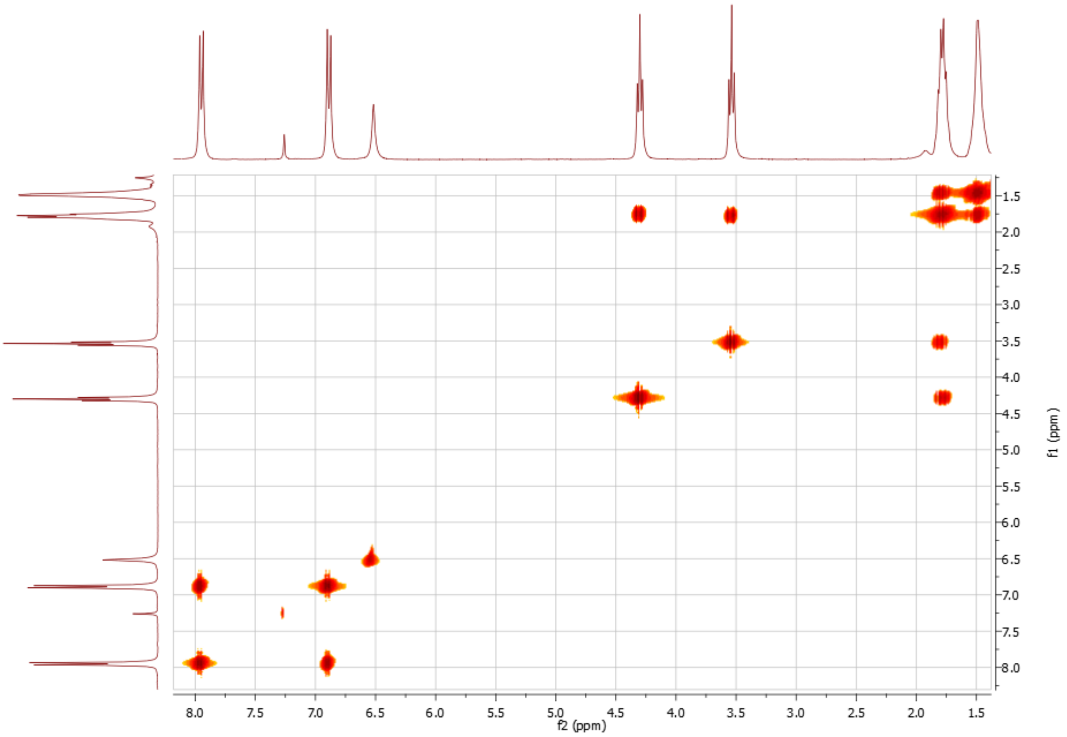

HSQC ($CDCl_3$): Compound **32**

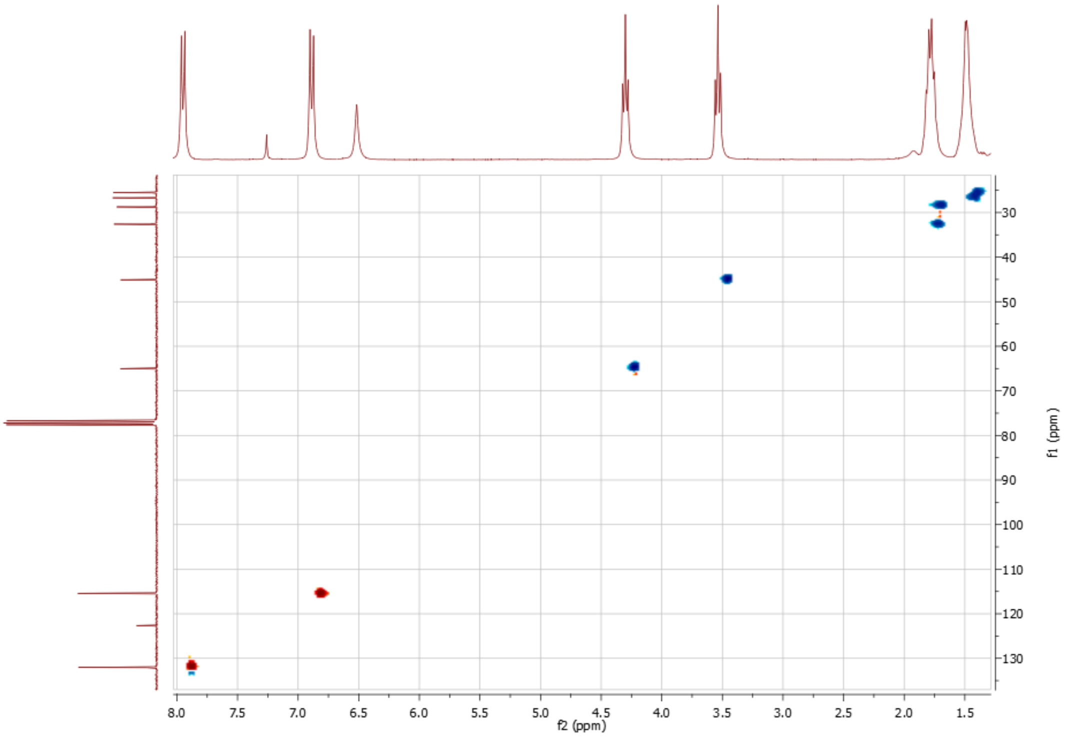

6-Chlorohexyl 4-(propargyloxy)benzoate (33)

[1]**H-NMR** (300 MHz, CDCl3): Compound **33**

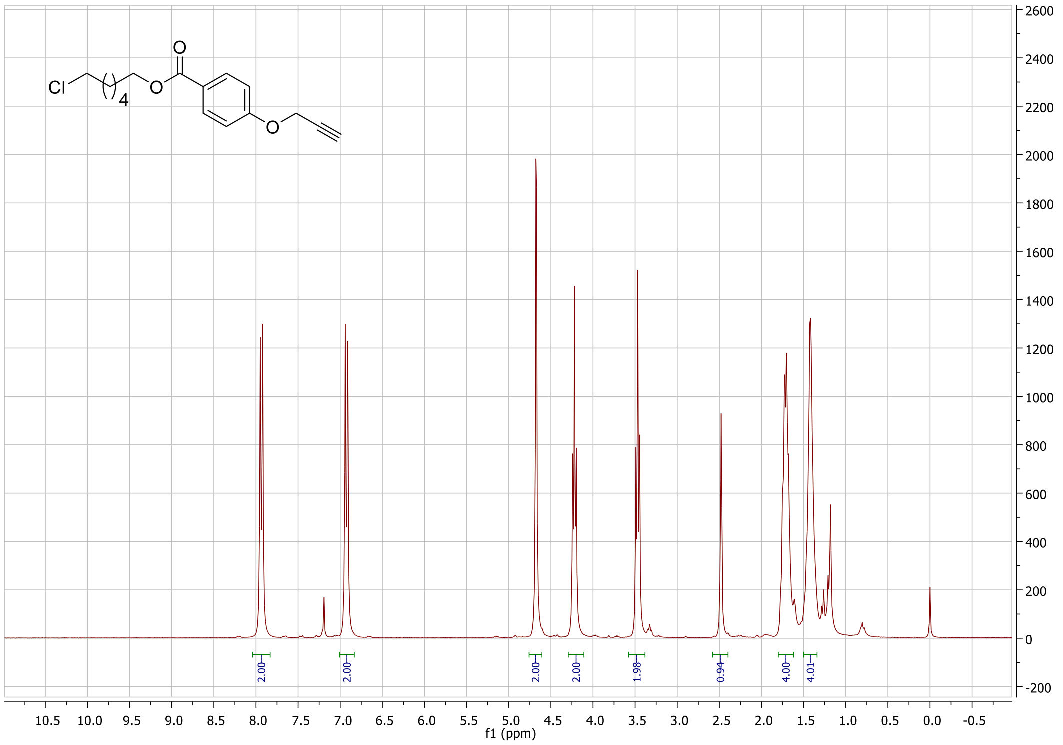

[13]**C NMR** (75.5 MHz, CDCl3): Compound **33**

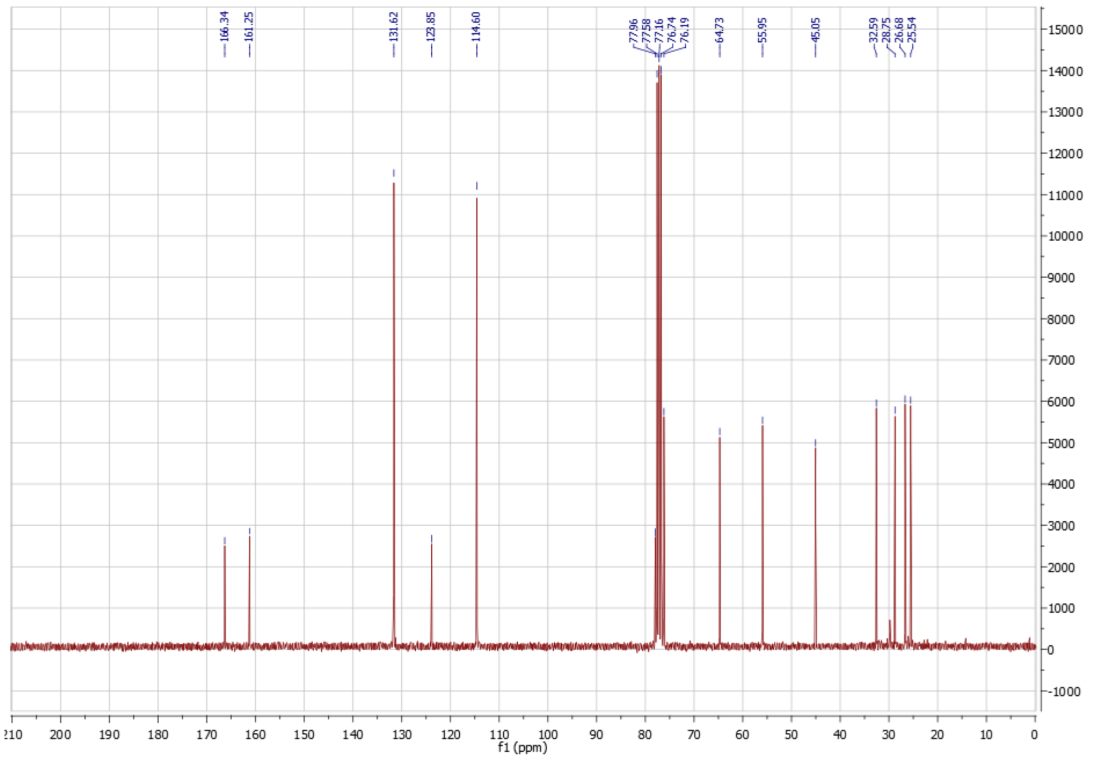

COSY (CDCl3): Compound **33**

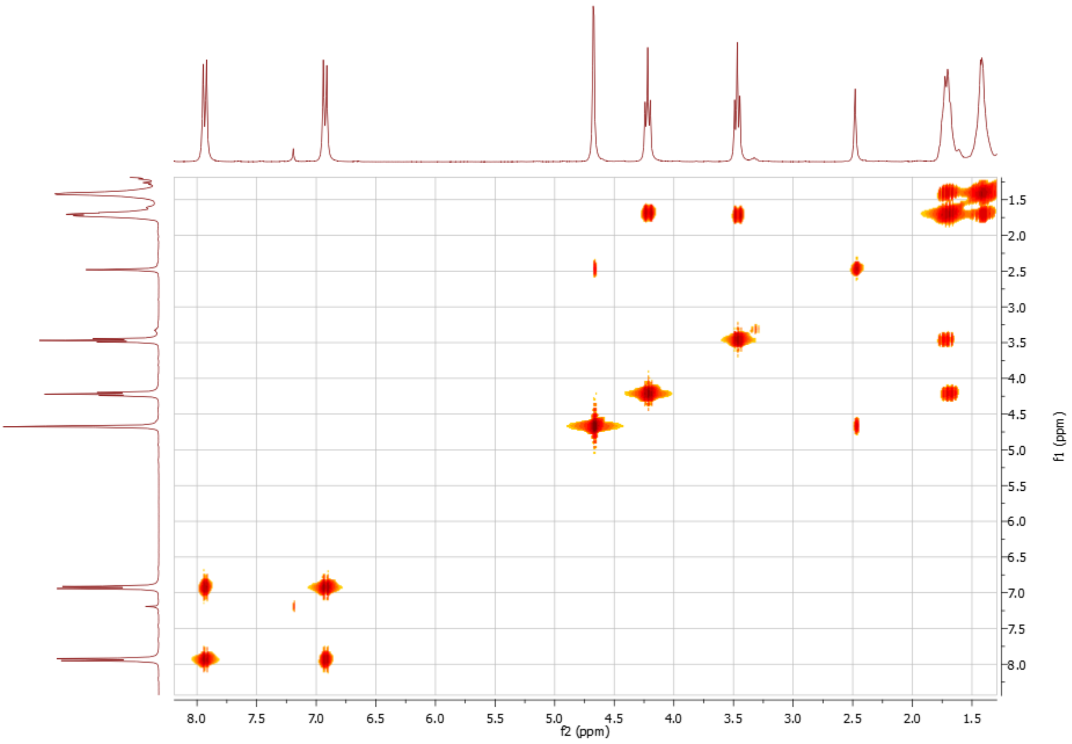

HSQC (CDCl3): Compound **33**

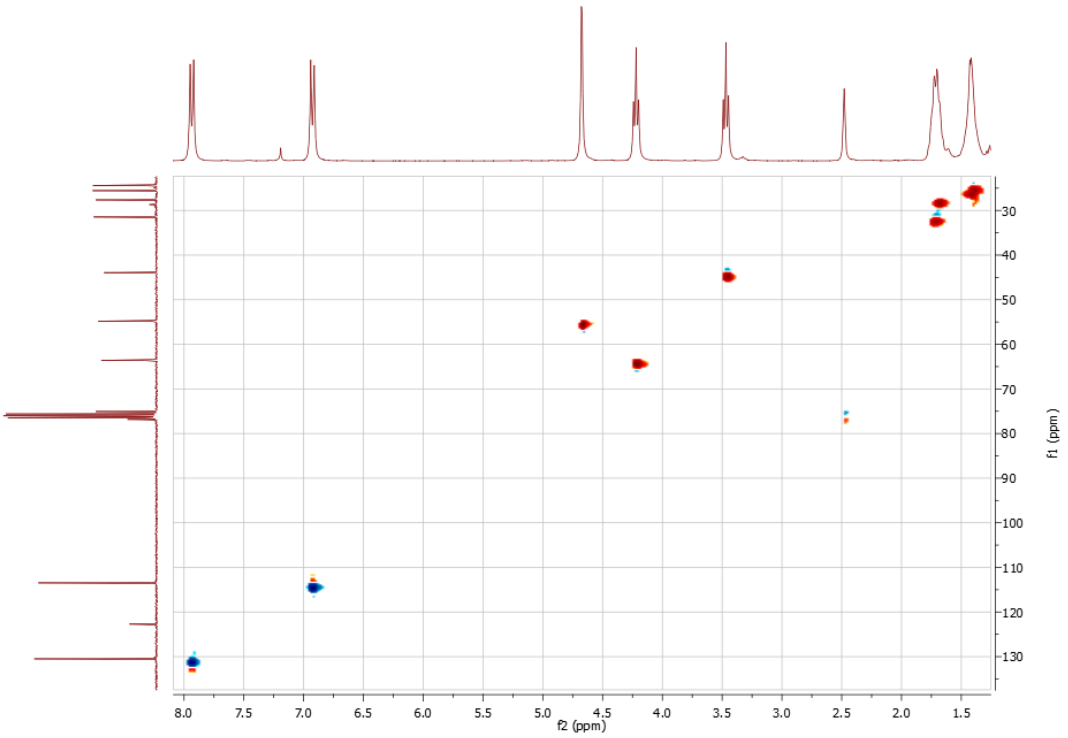

6-Hydroxyhexyl 4-(propargyloxy)benzoate (34)

[1]**H-NMR** (300 MHz, $CDCl_3$): Compound **34**

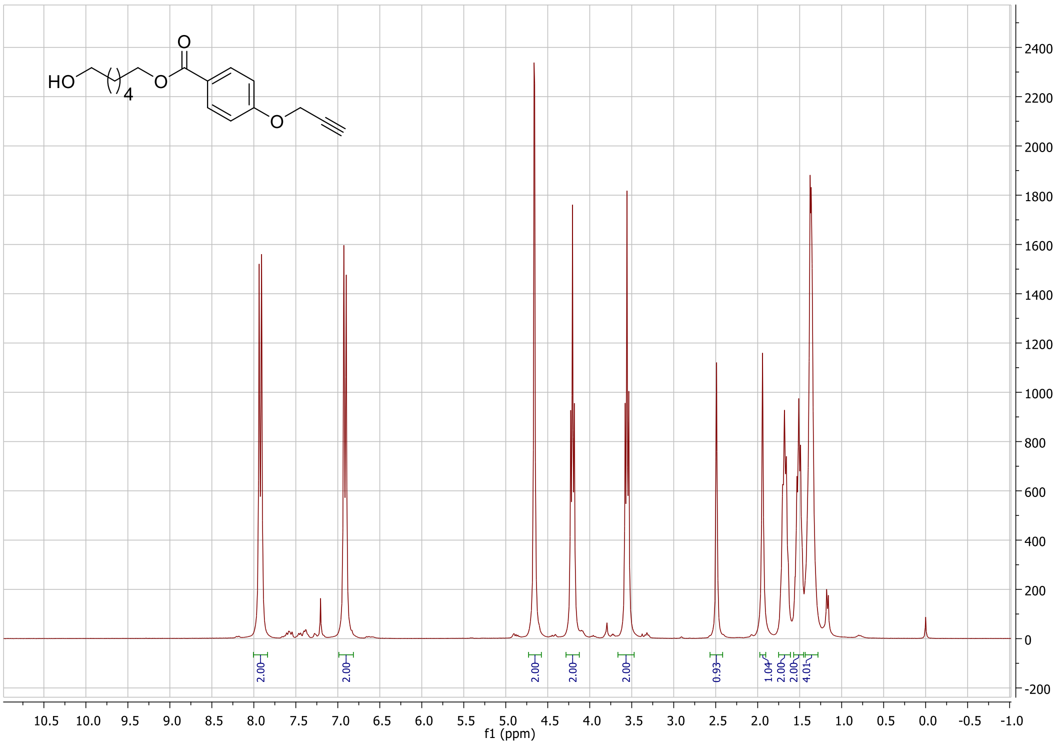

[13]**C-NMR** (75.5 MHz, $CDCl_3$): Compound **34**

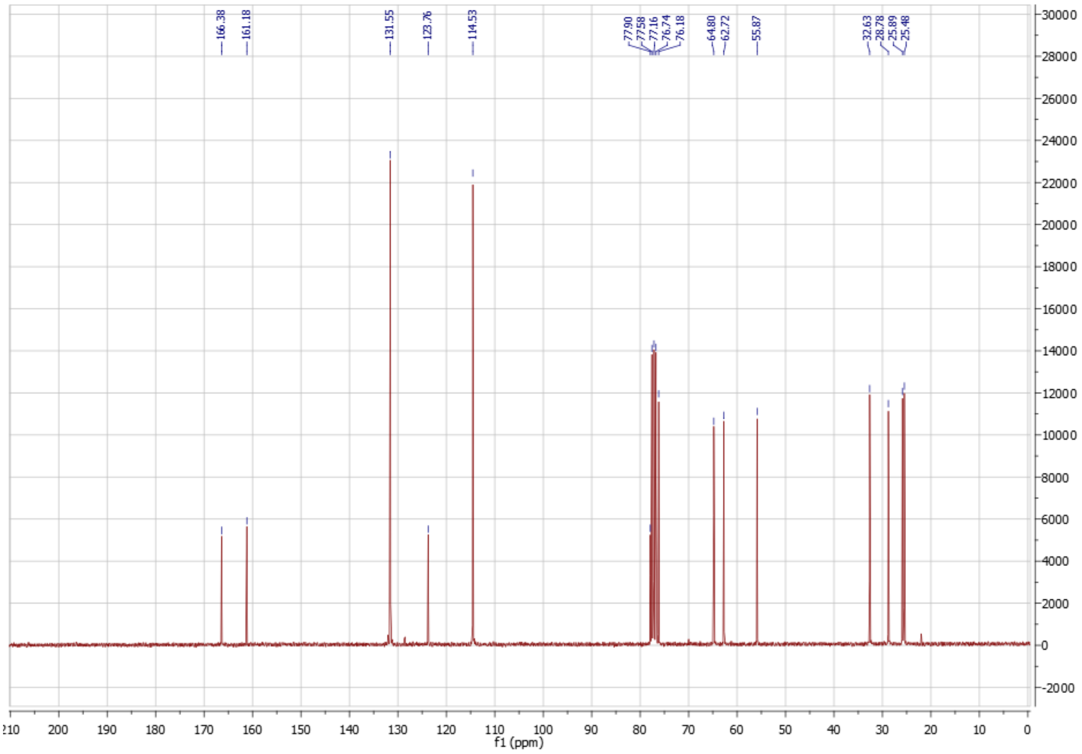

COSY (CDCl3): Compound **34**

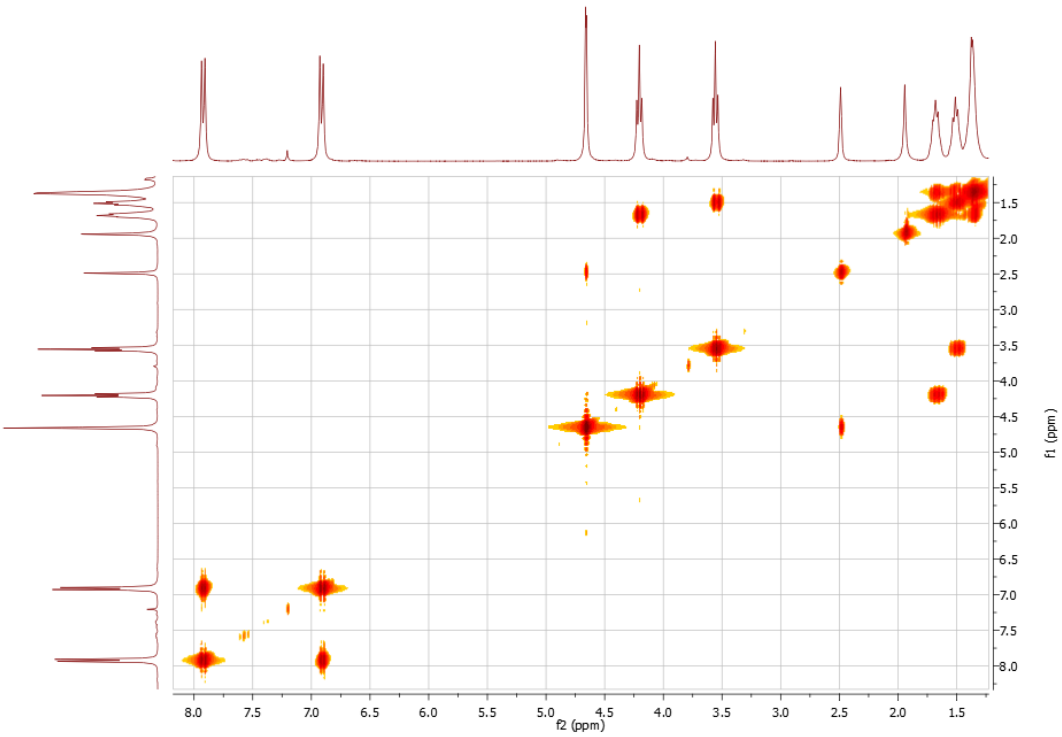

HSQC (CDCl3): Compound **34**

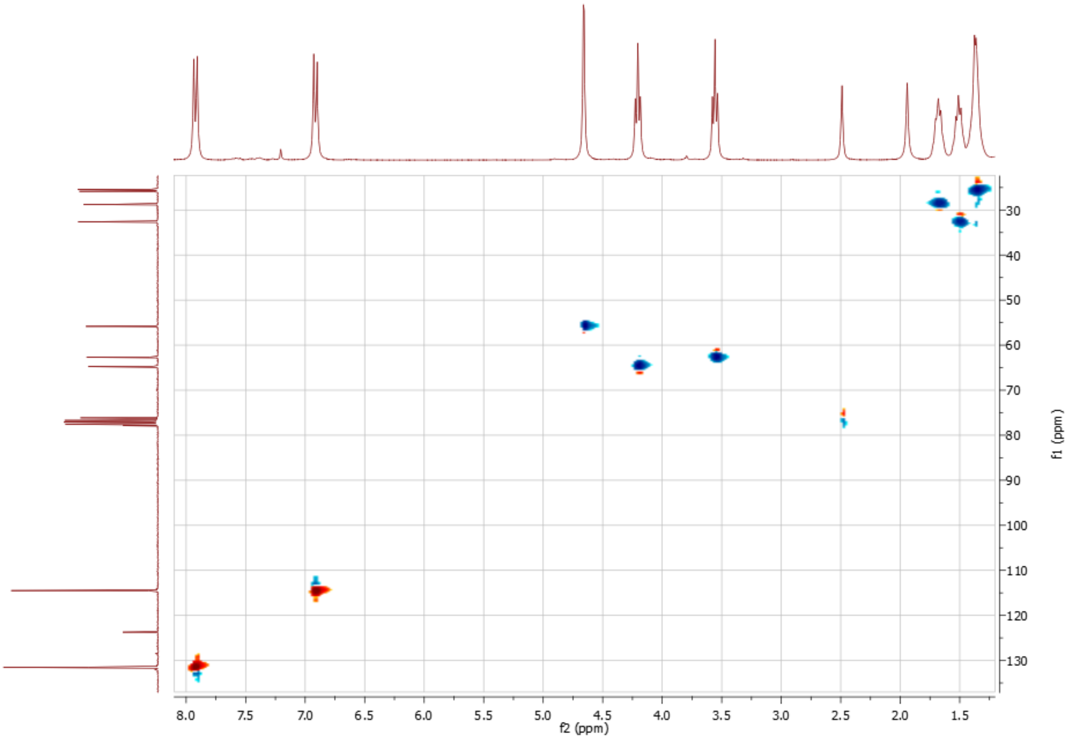

6-Oxohexyl 4-(propargyloxy)benzoate (35)

[1]**H-NMR** (300 MHz, CDCl3): Compound **35**

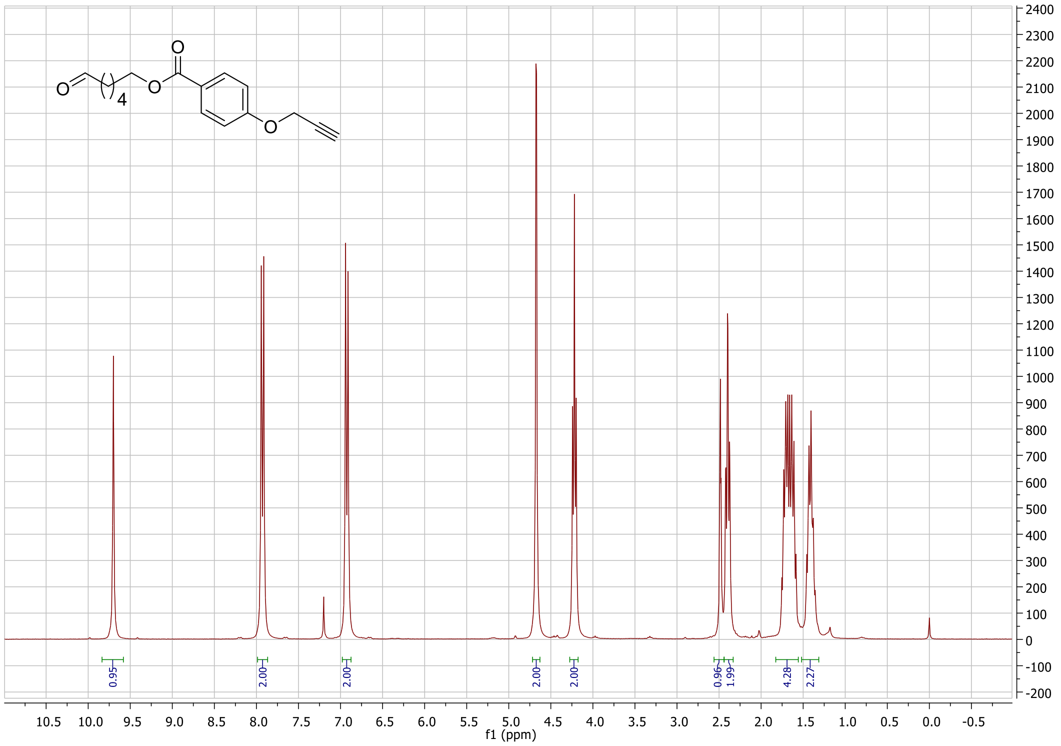

[13]**C-NMR** (75.5 MHz, CDCl3): Compound **35**

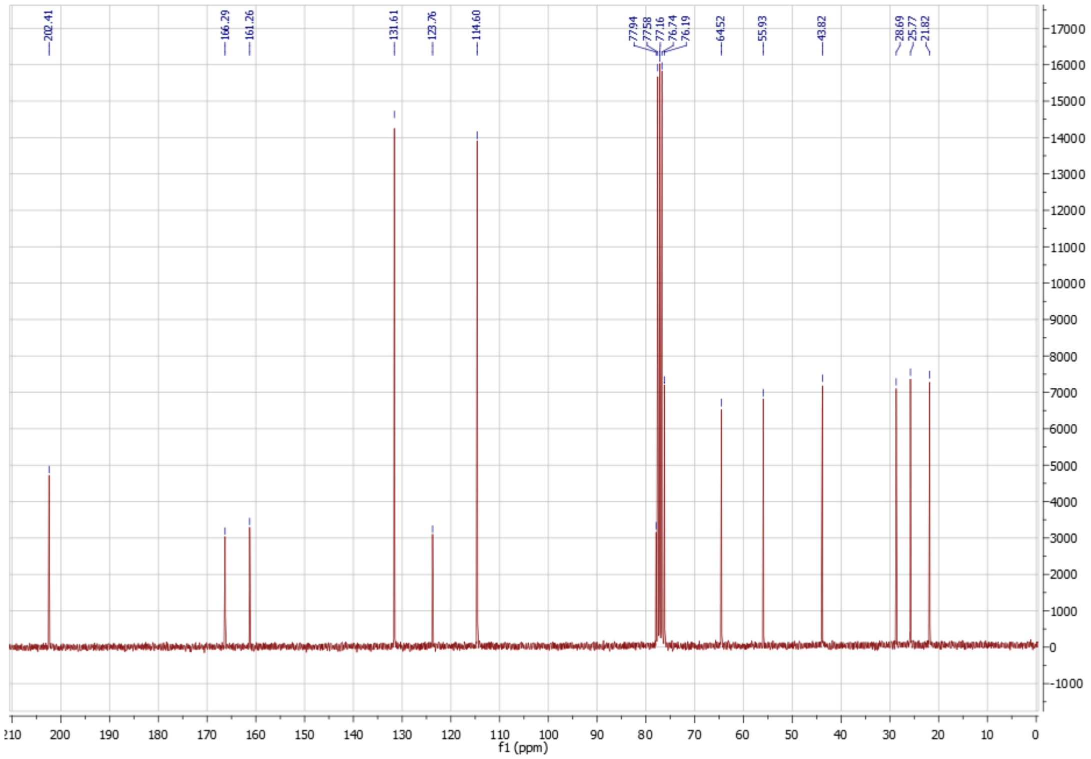

COSY (CDCl₃): Compound **35**

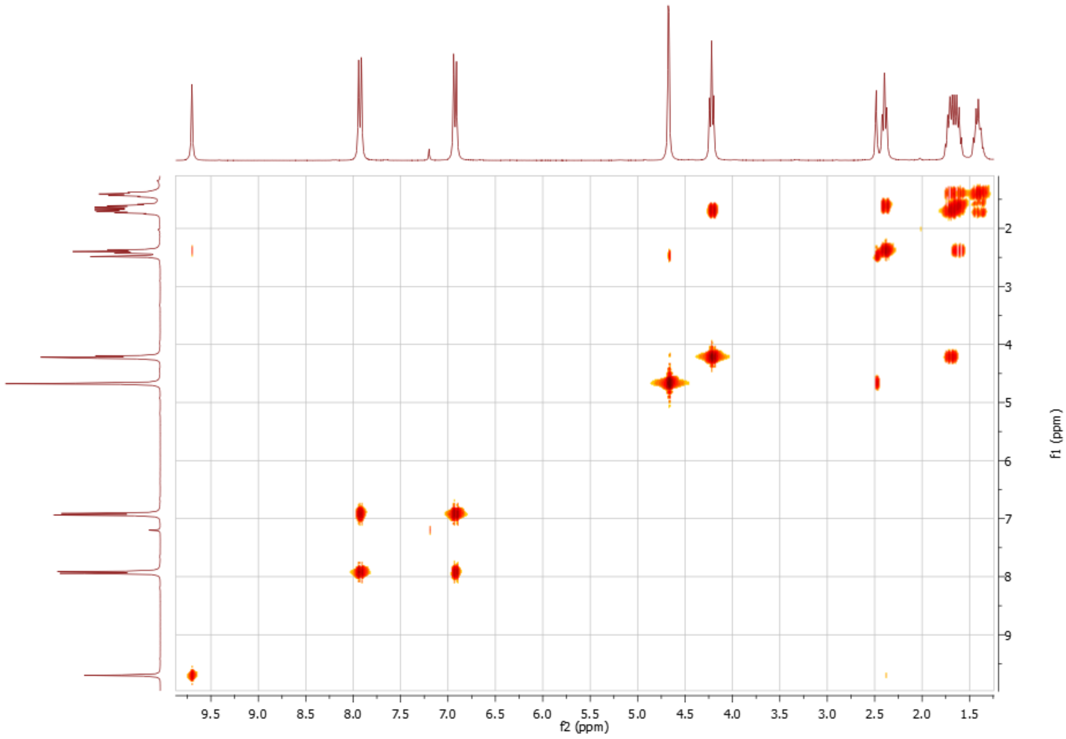

HSQC (CDCl₃): Compound **35**

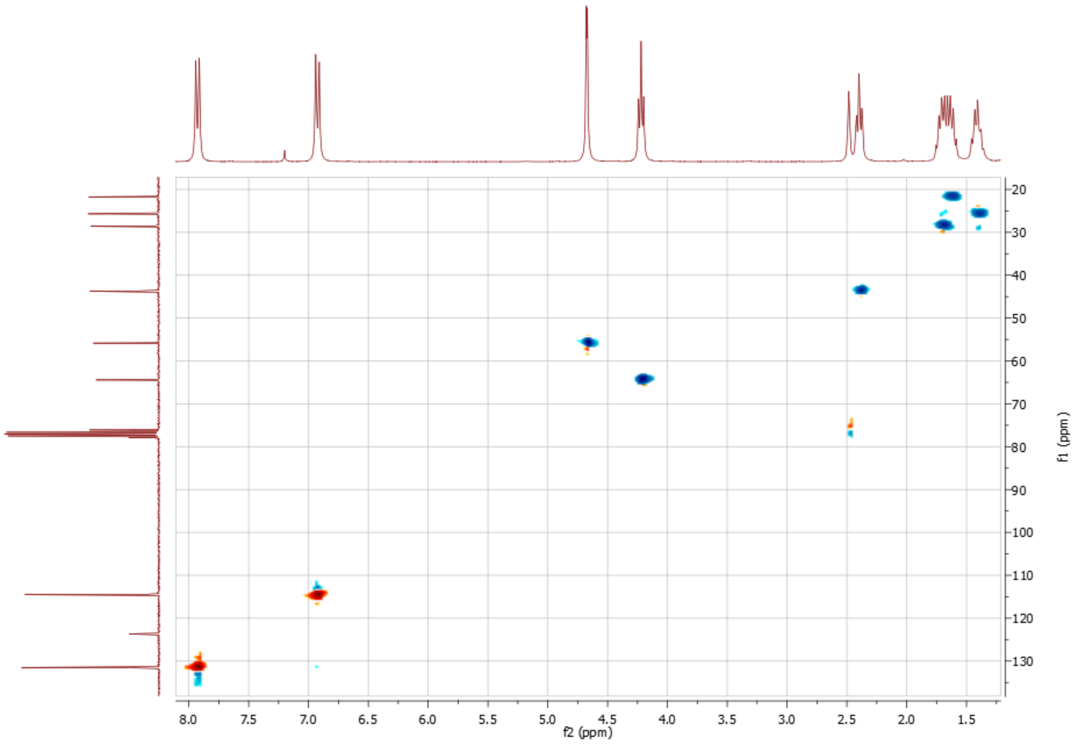

Methyl 4-(2-(2-((nonafluoro-*tert*-butyl)oxy)ethoxy)ethoxy)benzoate (37)

^{1}H-NMR (300 MHz, $CDCl_3$): Compound **37**

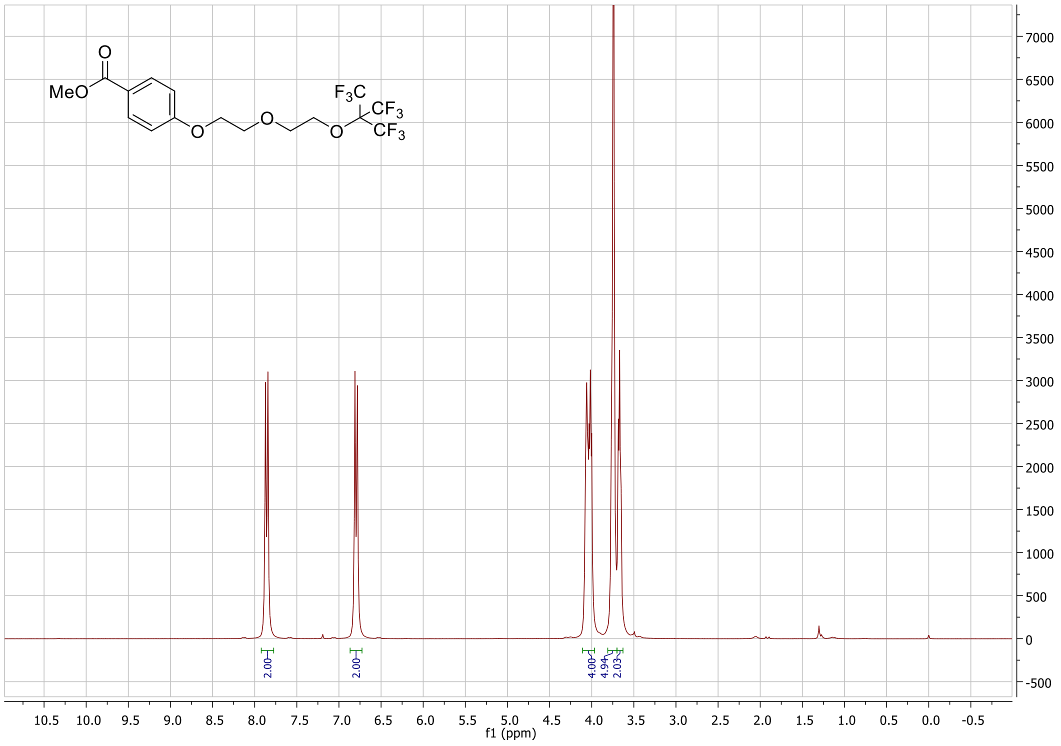

^{13}C-NMR (75.5 MHz, $CDCl_3$): Compound **37**

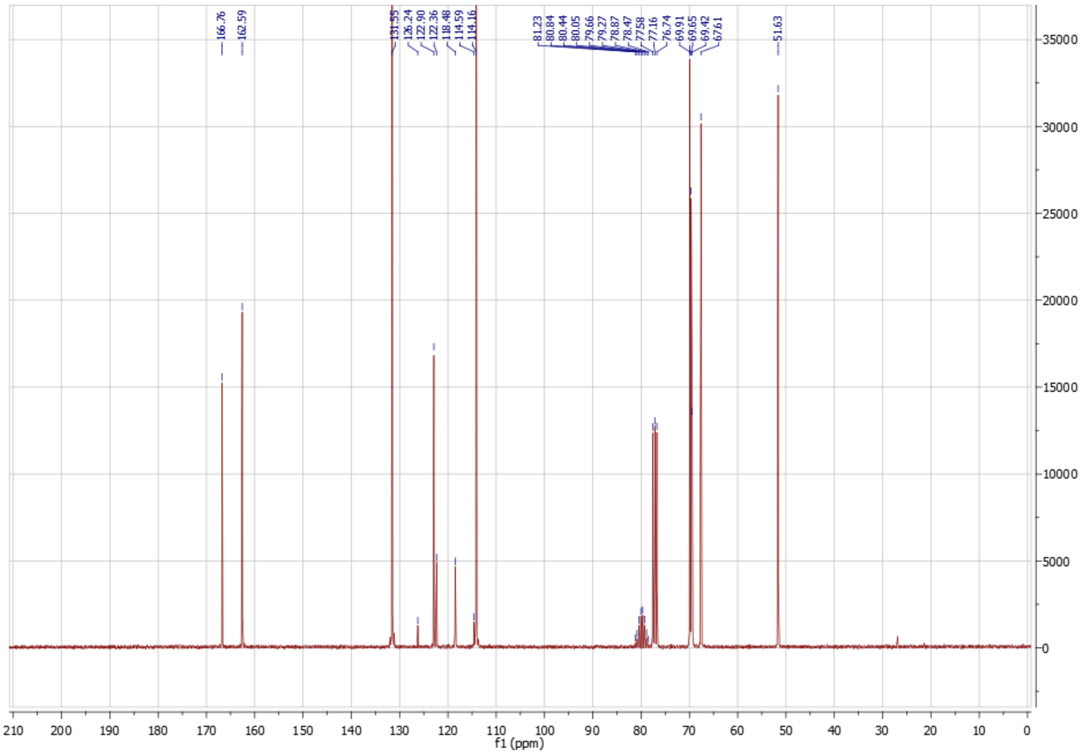

COSY ($CDCl_3$): Compound **37**

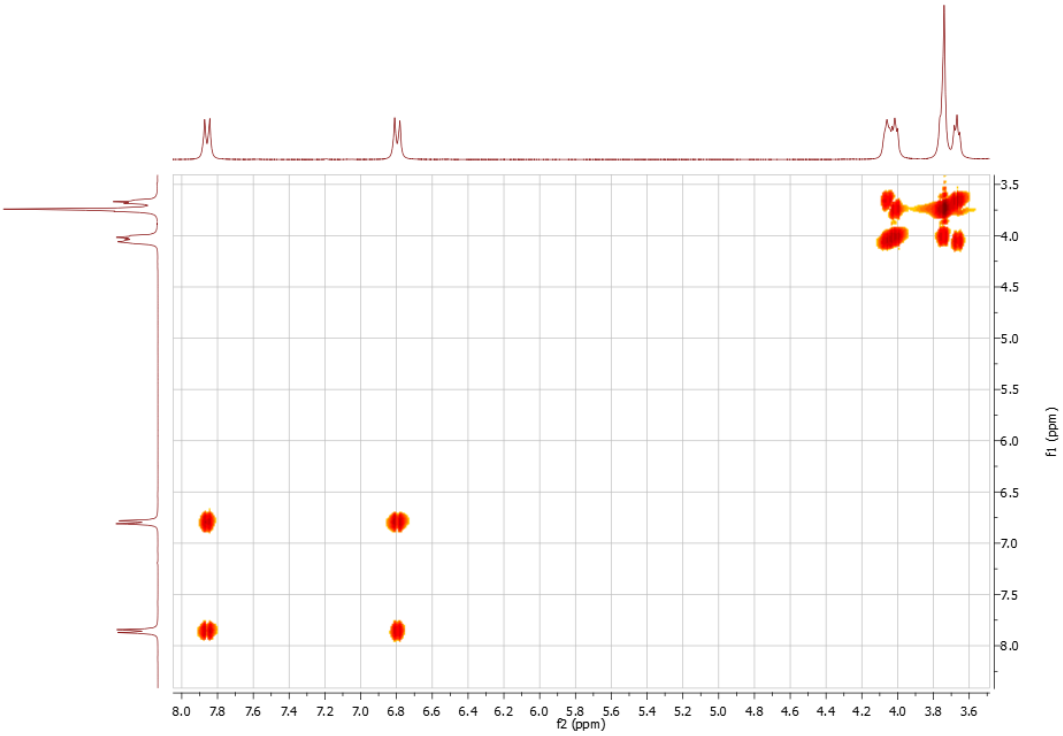

HSQC ($CDCl_3$): Compound **37**

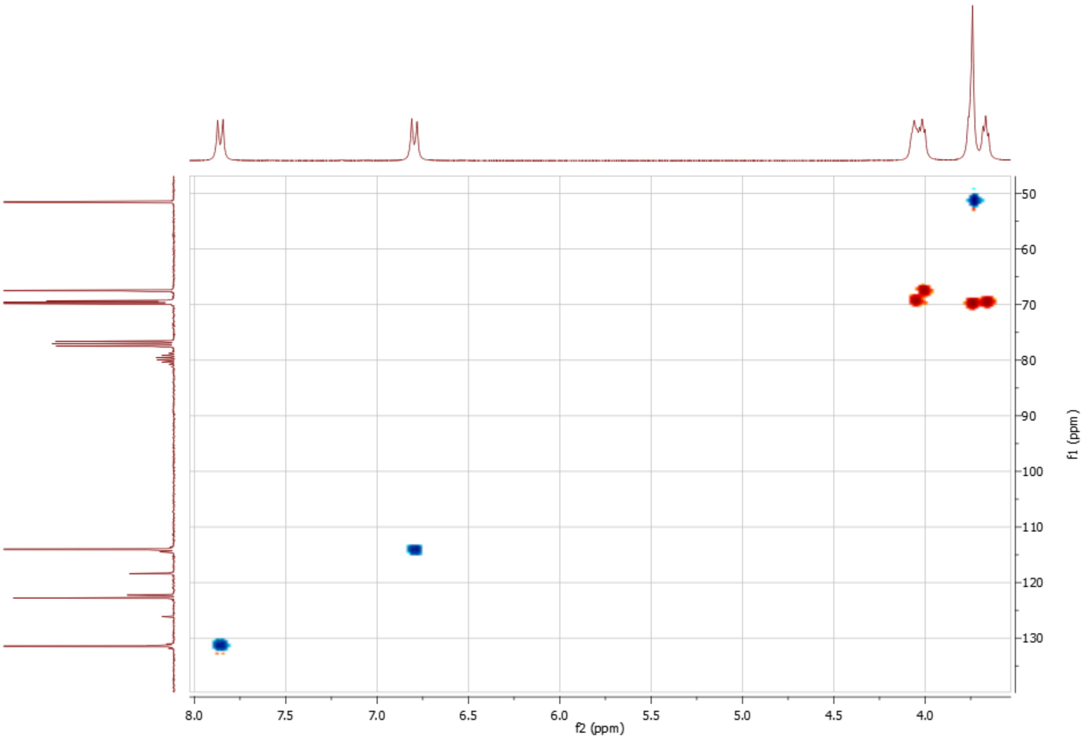

6-Hydroxyhexyl 4-(2-(2-((nonafluoro-*tert*-butyl)oxy)ethoxy)ethoxy)benzoate (40)

^{1}H-NMR (300 MHz, $CDCl_3$): Compound **40**

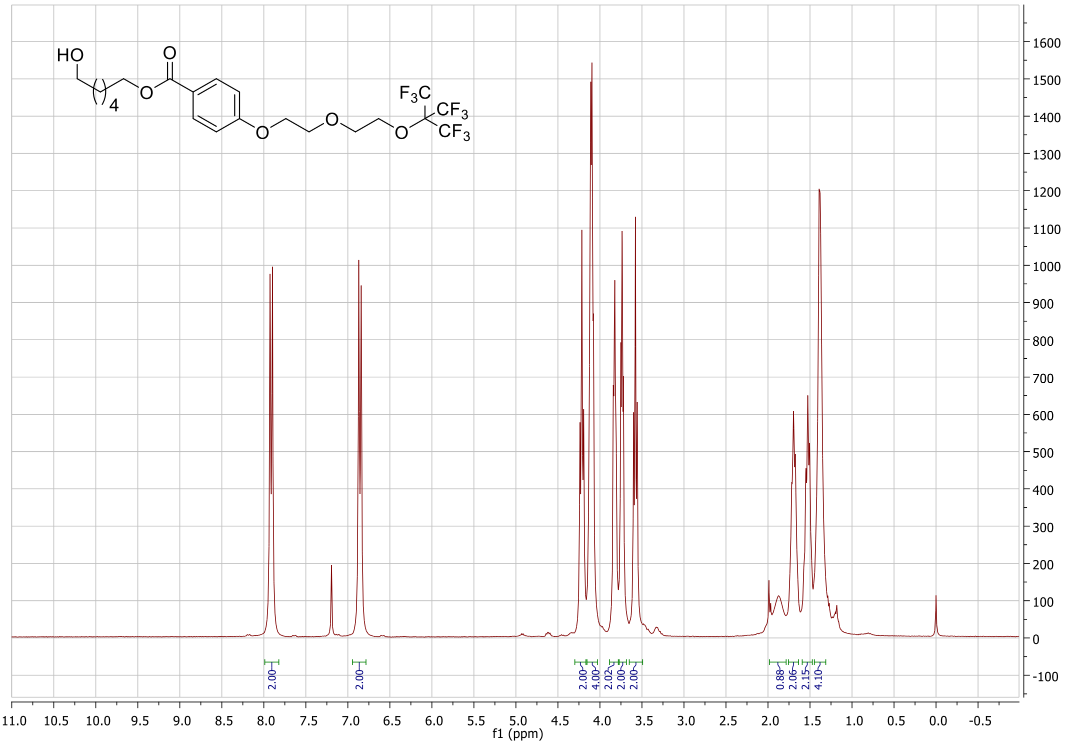

^{13}C-NMR (75.5 MHz, $CDCl_3$): Compound **40**

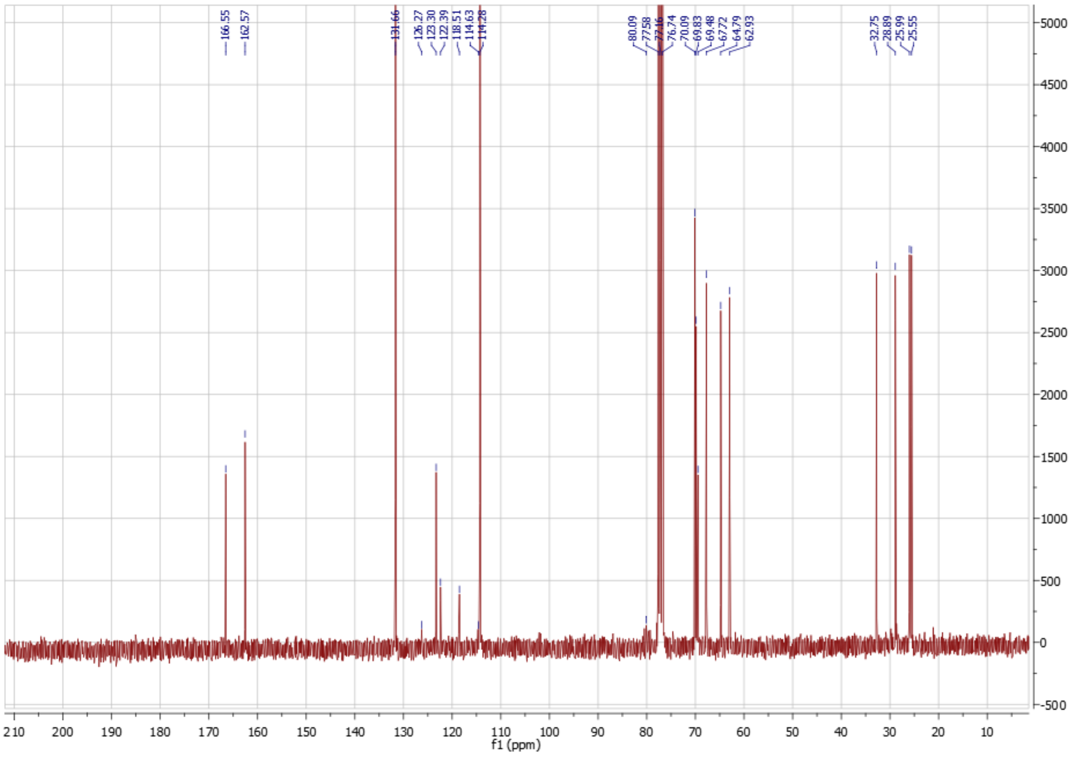

COSY (CDCl3): Compound **40**

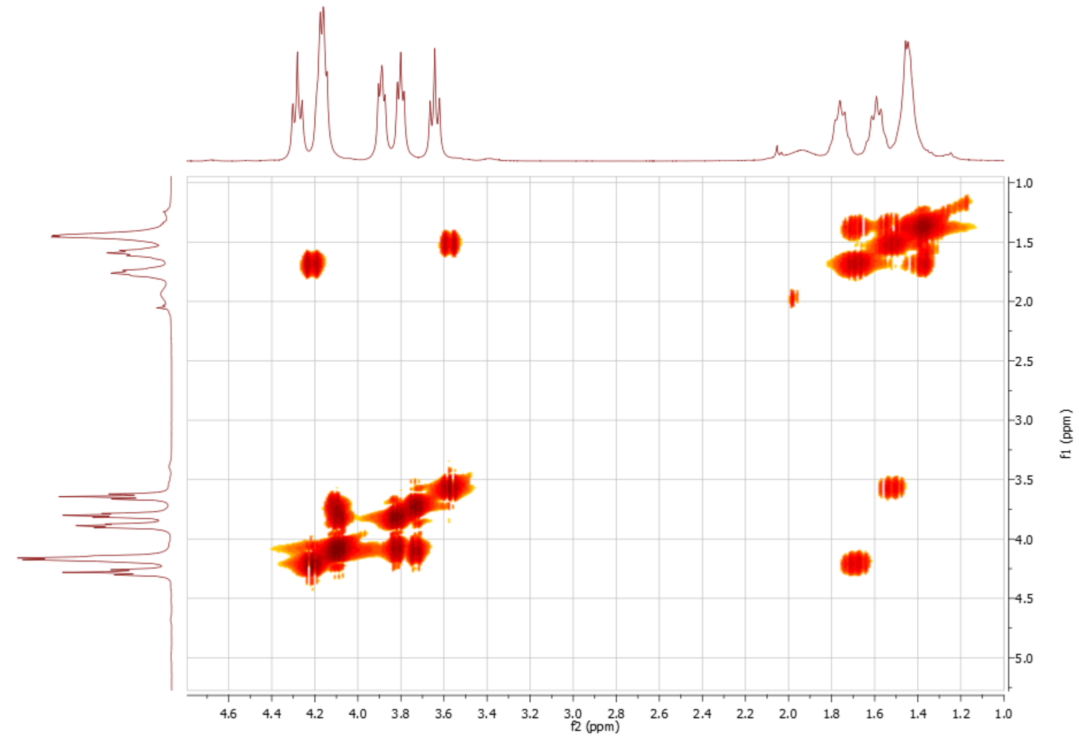

HSQC (CDCl3): Compound **40**

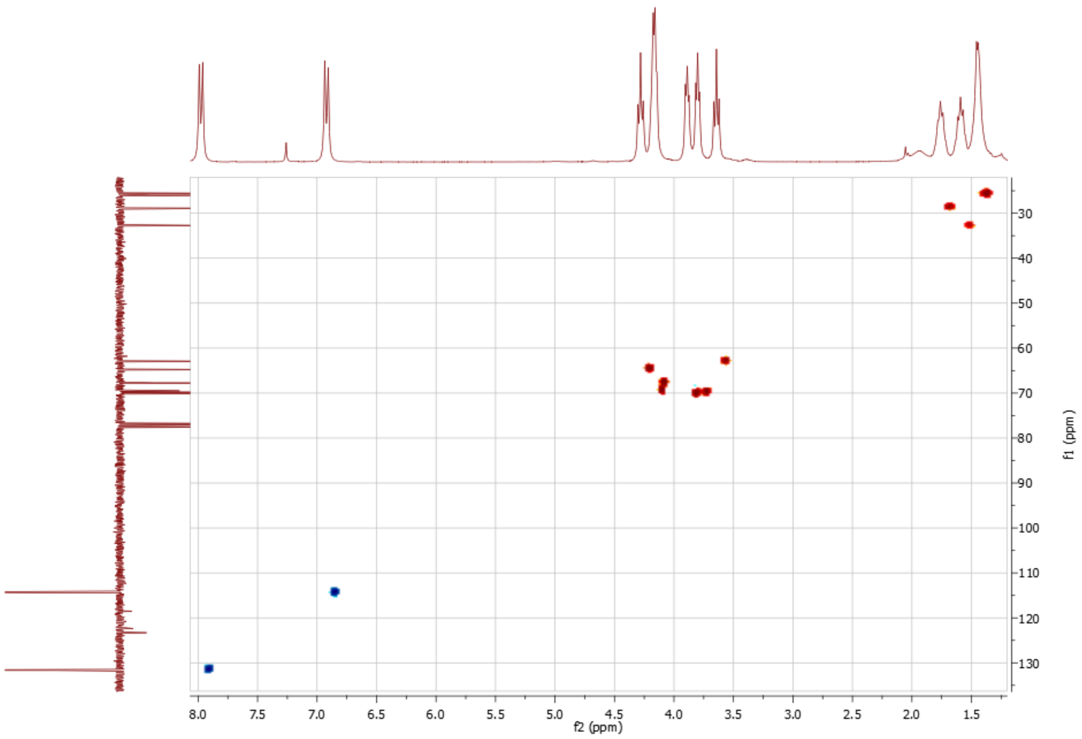

6-Oxohexyl 4-(2-(2-((nonafluoro-*tert*-butyl)oxy)ethoxy)ethoxy)benzoate (41)

[1]**H-NMR** (300 MHz, $CDCl_3$): Compound **41**

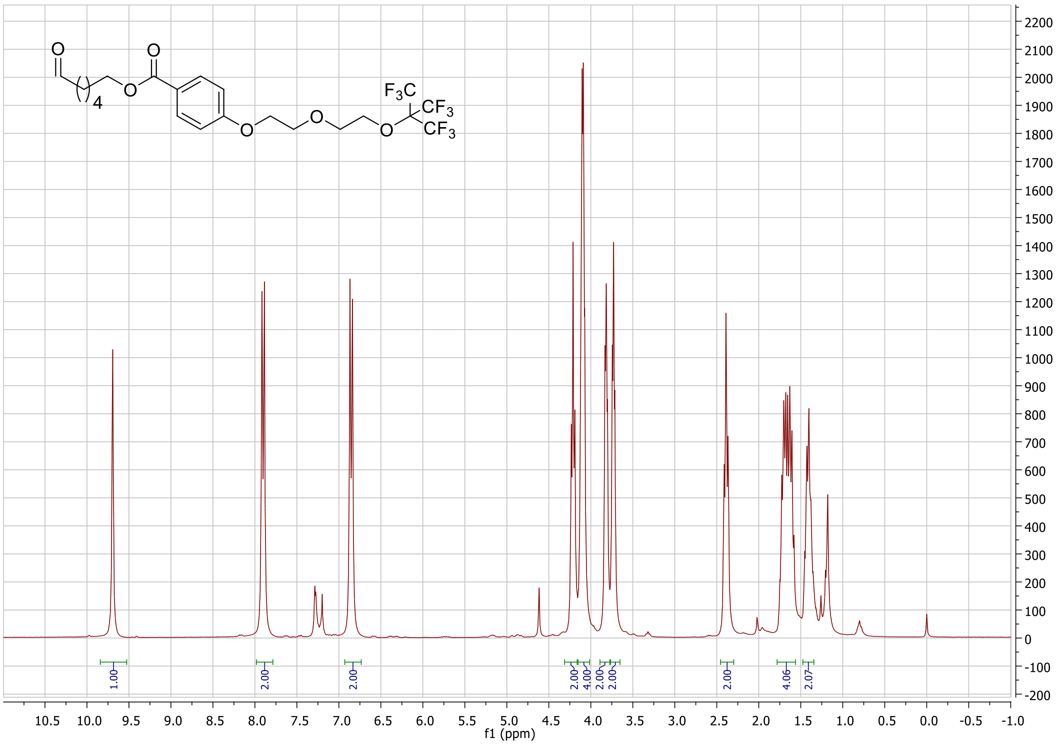

[13]**C-NMR** (75.5 MHz, $CDCl_3$): Compound **41**

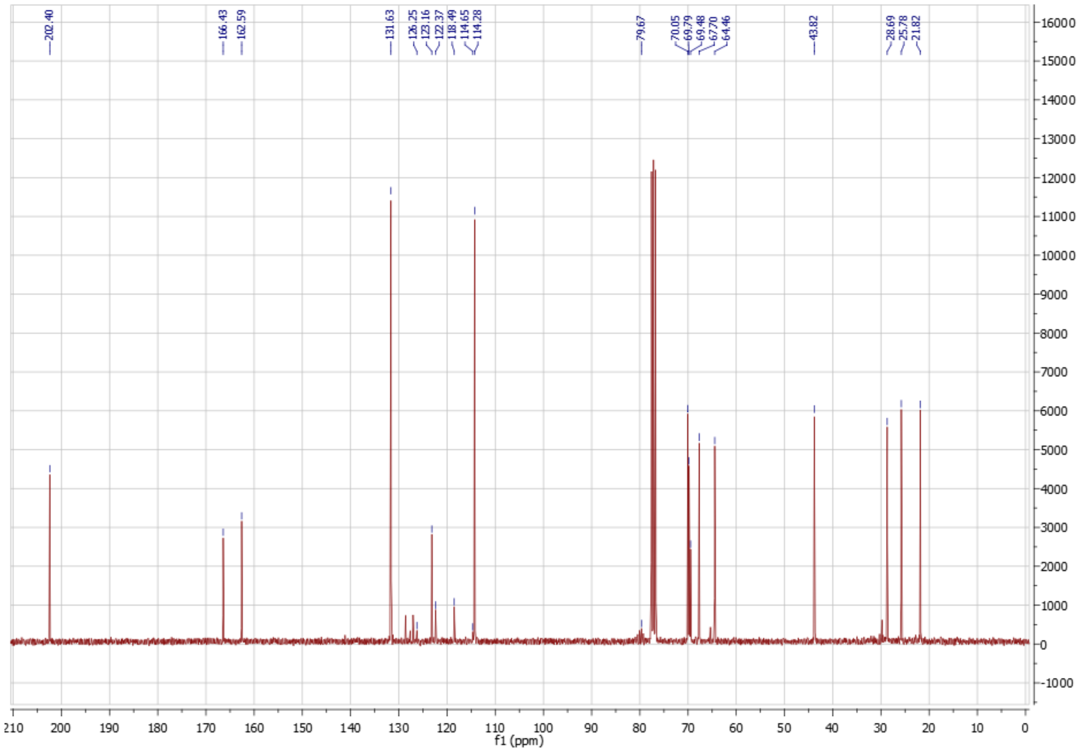

COSY ($CDCl_3$): Compound **41**

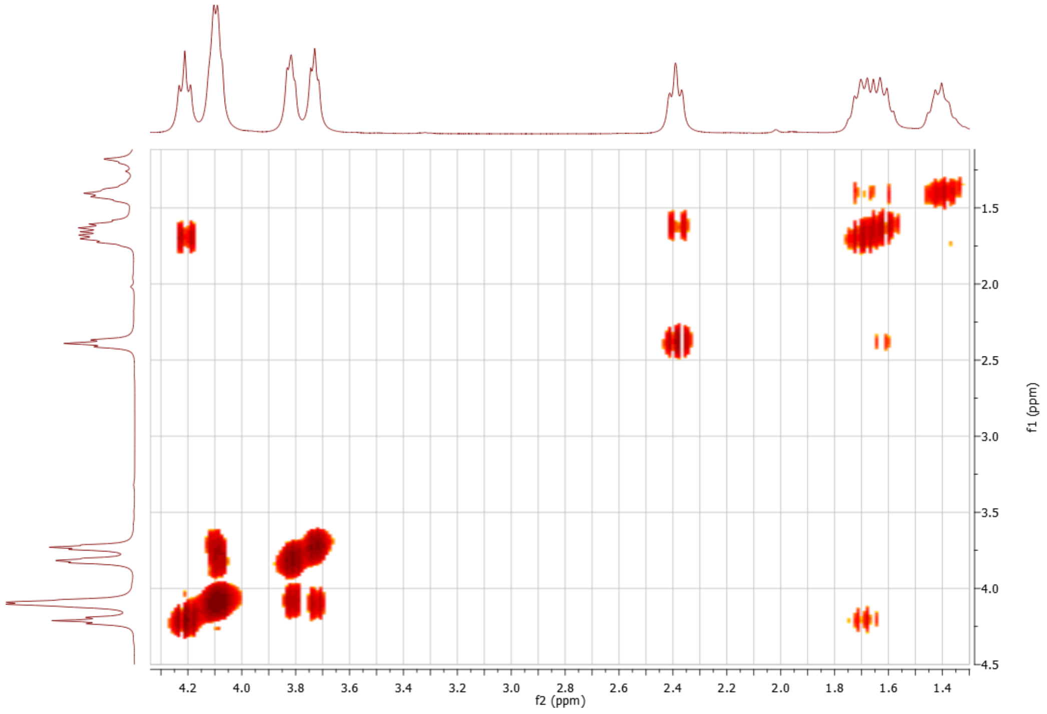

HSQC ($CDCl_3$): Compound **41**

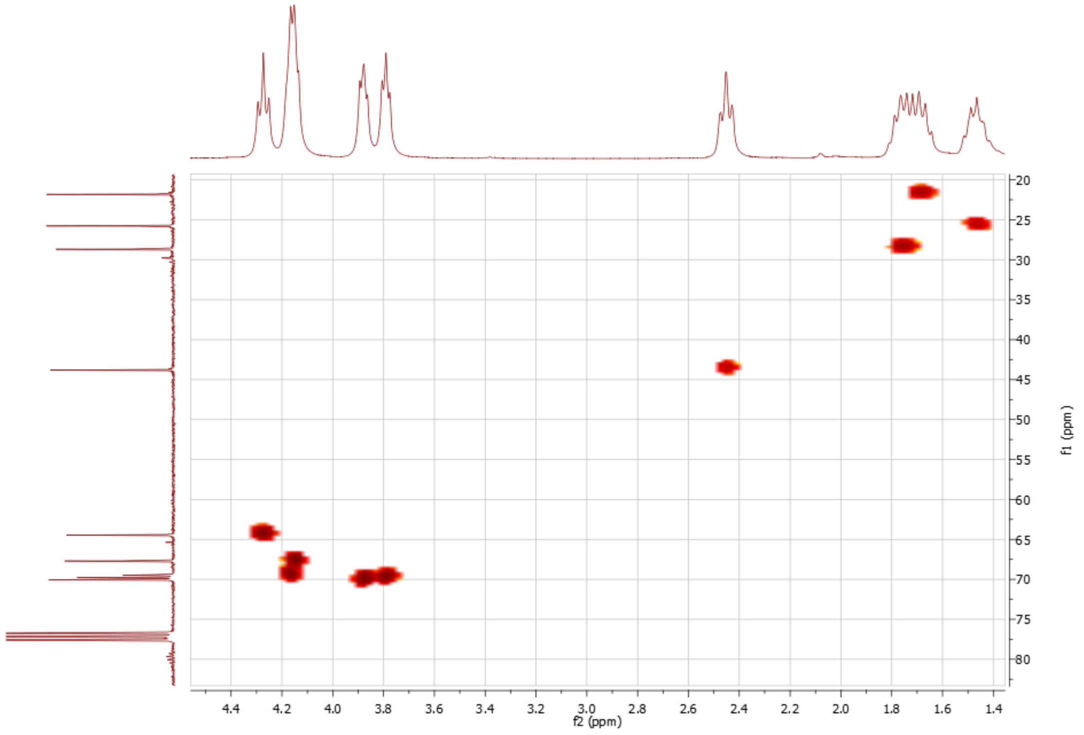

6-Bromohexyl 4-(2-(((benzyloxy)carbonyl)amino)ethyl)benzoate (43)

^{1}**H-NMR** (300 MHz, $CDCl_3$): Compound **43**

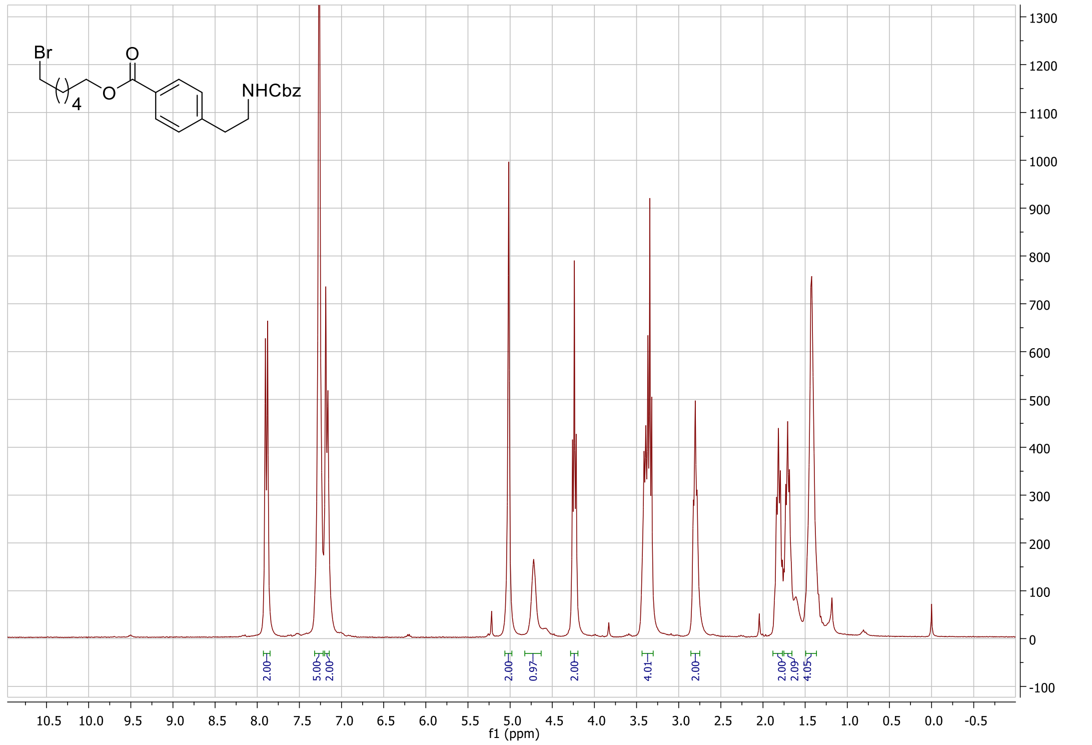

^{13}**C-NMR** (75.5 MHz, $CDCl_3$): Compound **43**

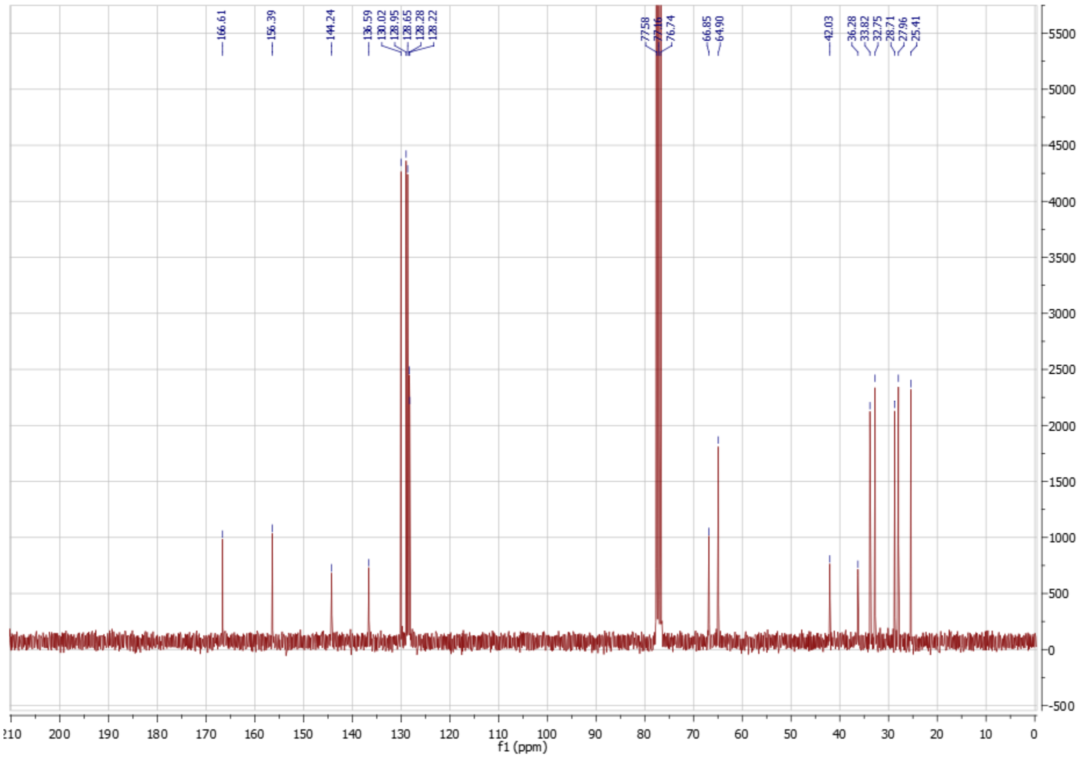

COSY ($CDCl_3$): Compound **43**

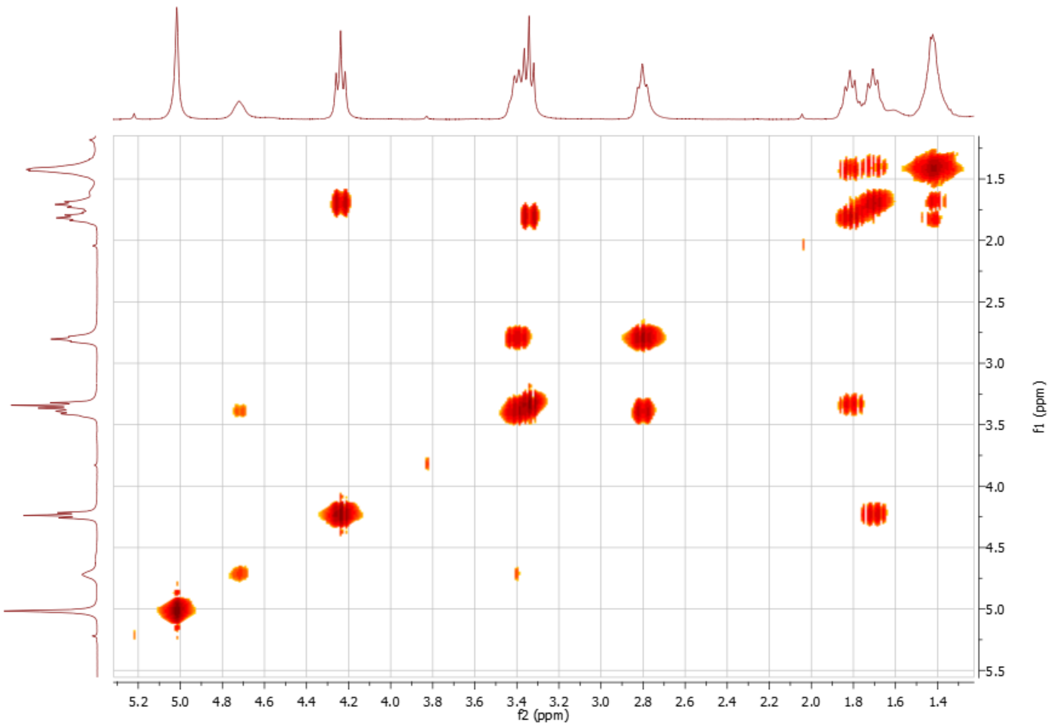

HSQC ($CDCl_3$): Compound **43**

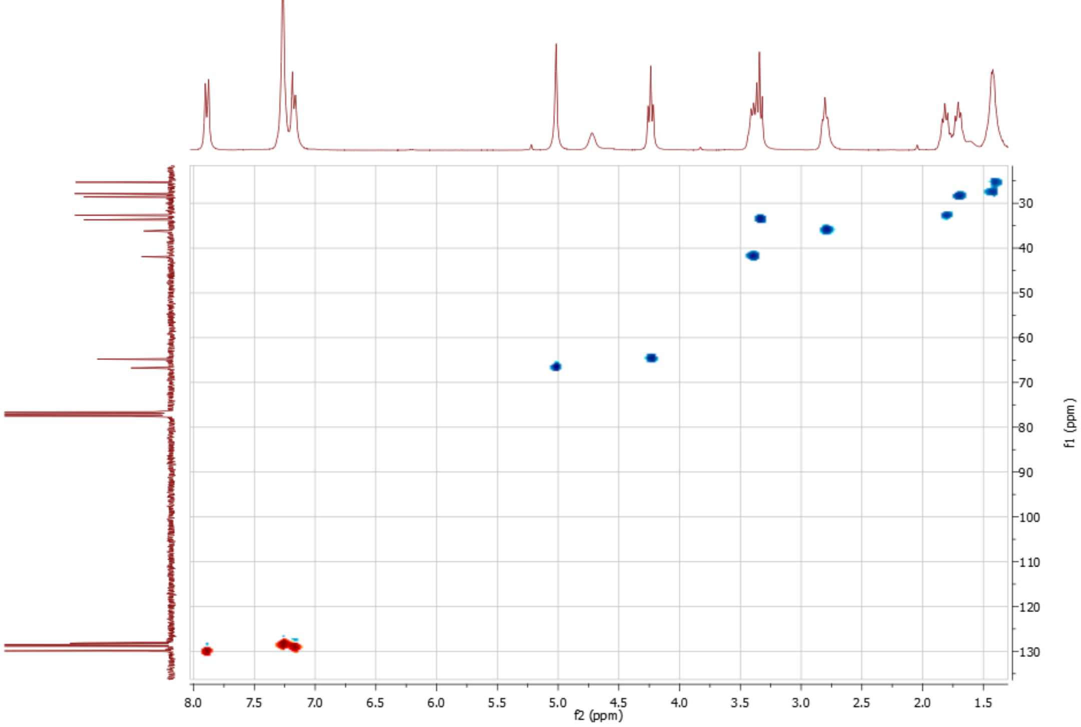

6-Oxohexyl 4-(2-(((benzyloxy)carbonyl)amino)ethyl)benzoate (44)

^{1}H-NMR (300 MHz, $CDCl_3$): Compound **44**

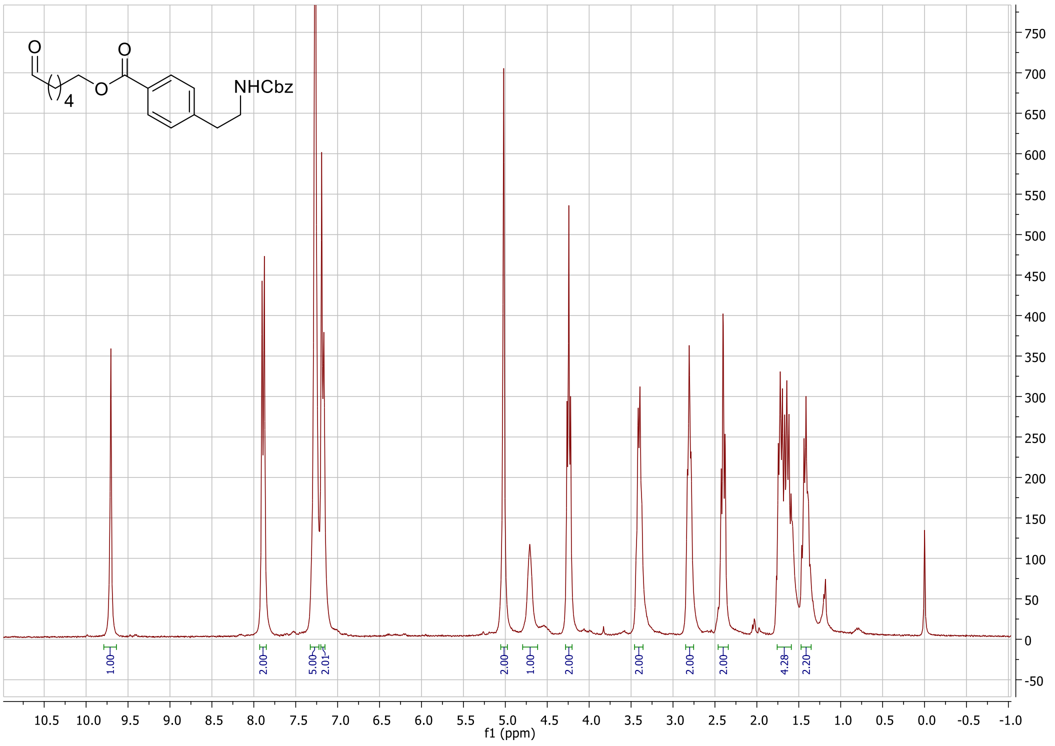

^{13}C-NMR (75.5 MHz, $CDCl_3$): Compound **44**

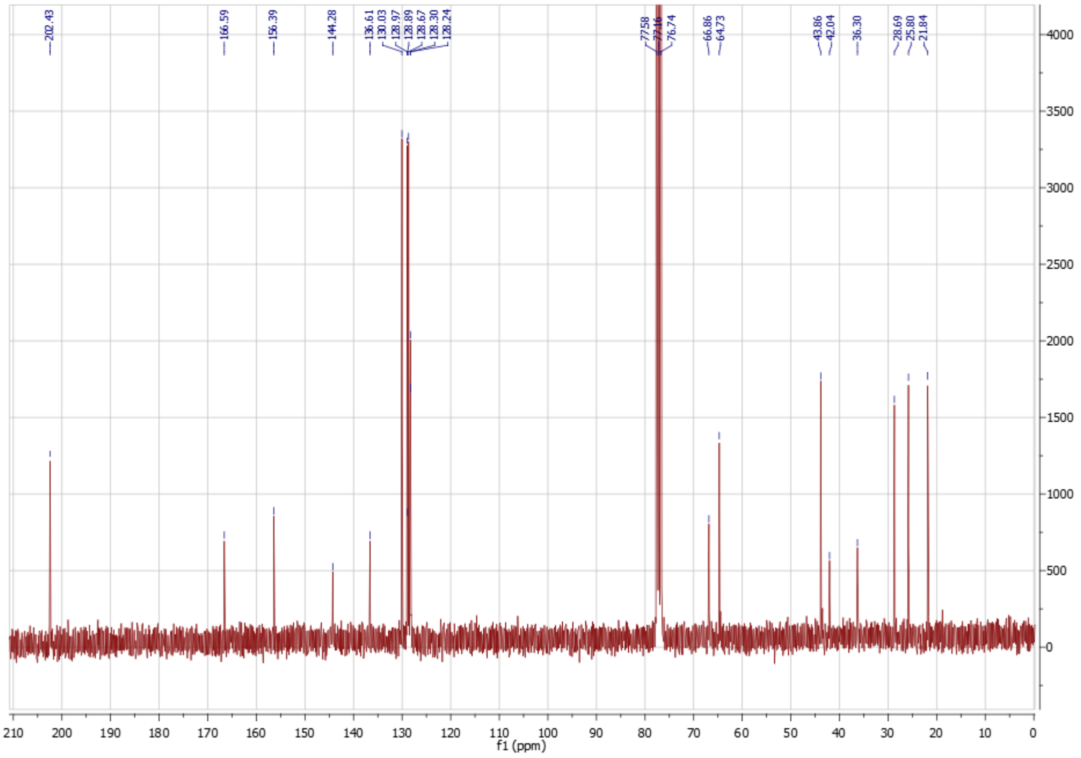

COSY ($CDCl_3$): Compound **44**

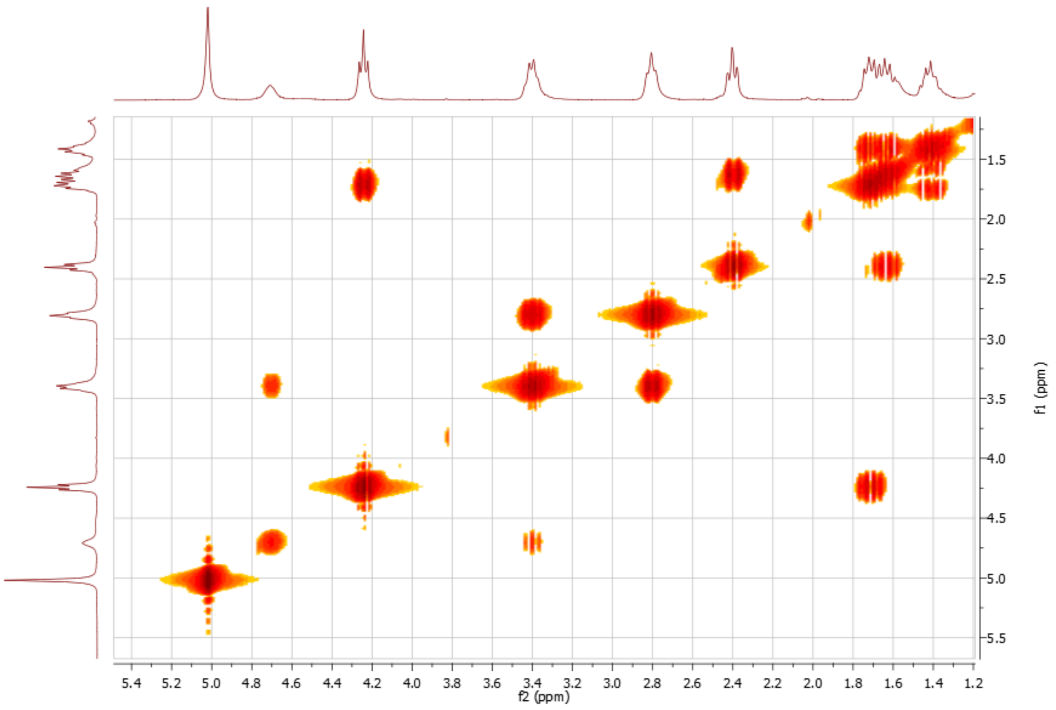

HSQC ($CDCl_3$): Compound **44**

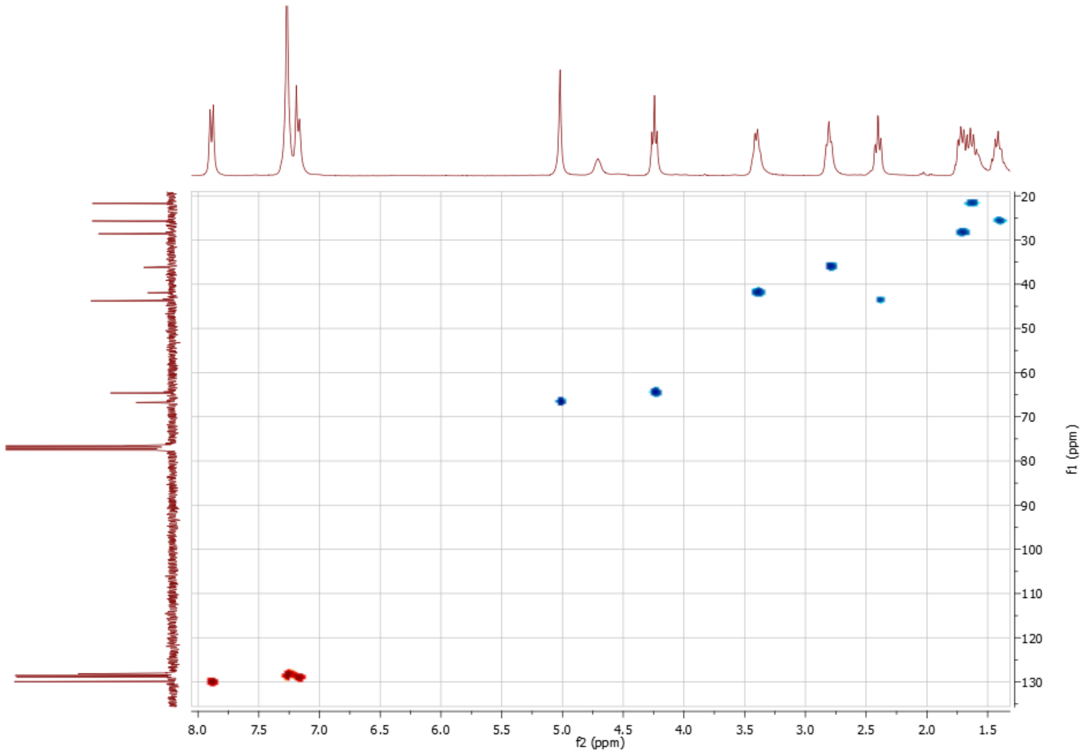

N-(6-((4-((5-Cyanopentyl)oxy)benzoyl)oxy)hexyl)-1,5-dideoxy-1,5-imino-D-glucitol (45)

^{1}H-NMR (300 MHz, CD_3OD): Compound **45**

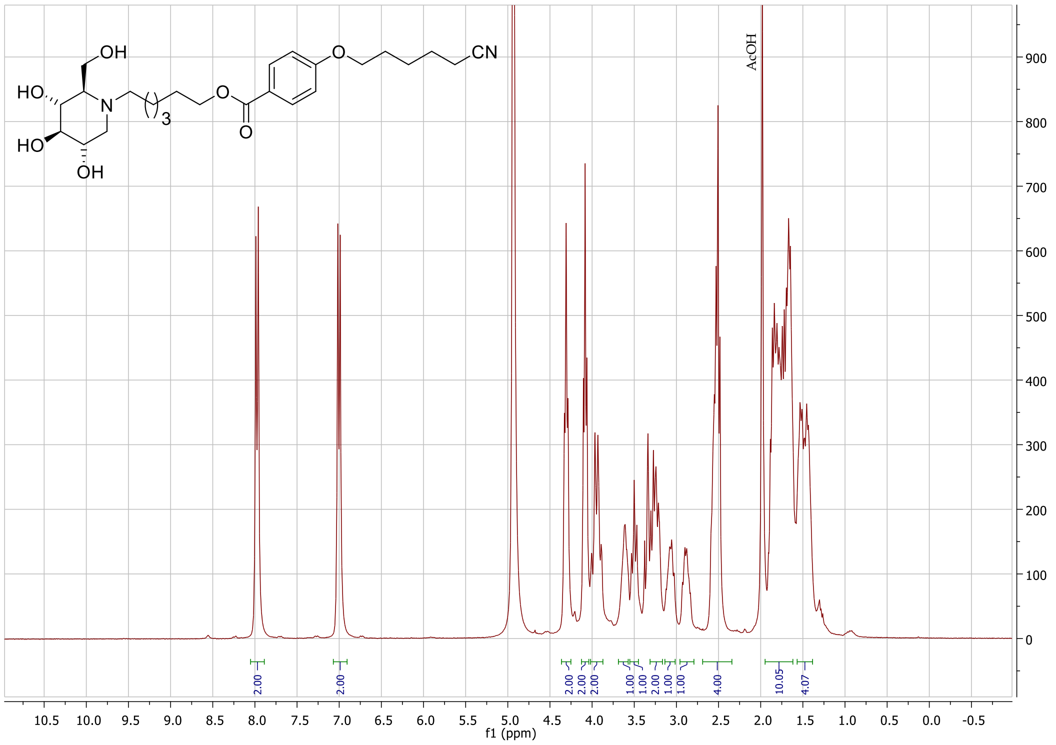

^{13}C-NMR (75.5 MHz, CD_3OD): Compound **45**

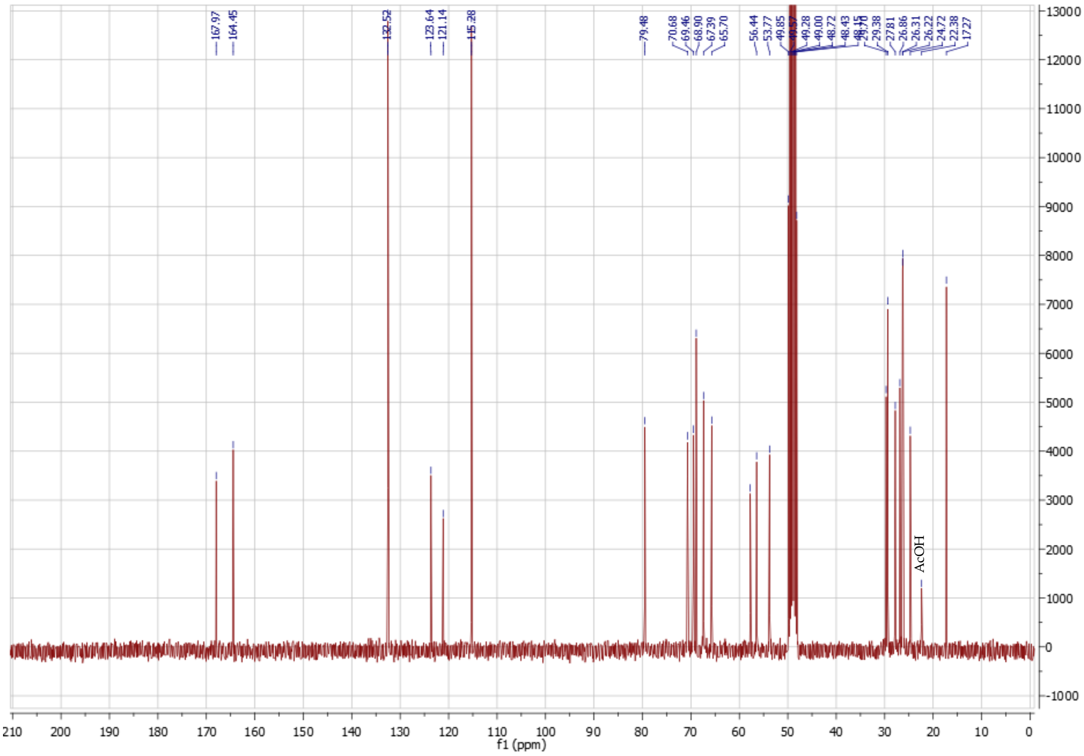

COSY (CD3OD): Compound **45**

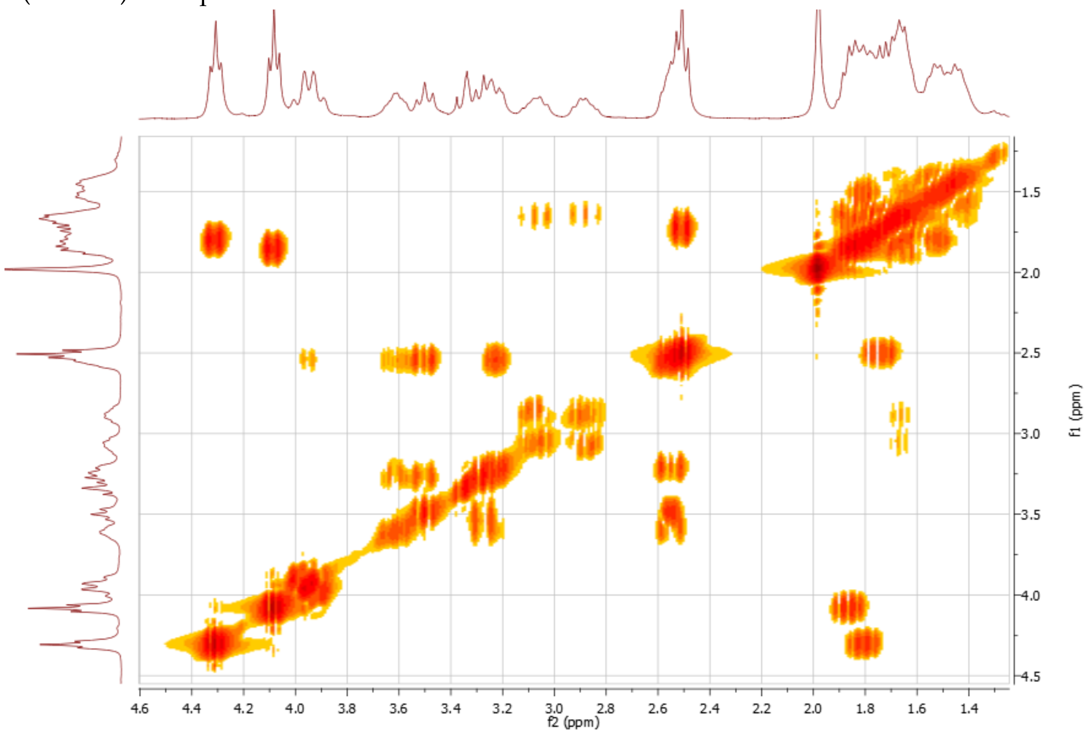

HSQC (CD3OD): Compound **45**

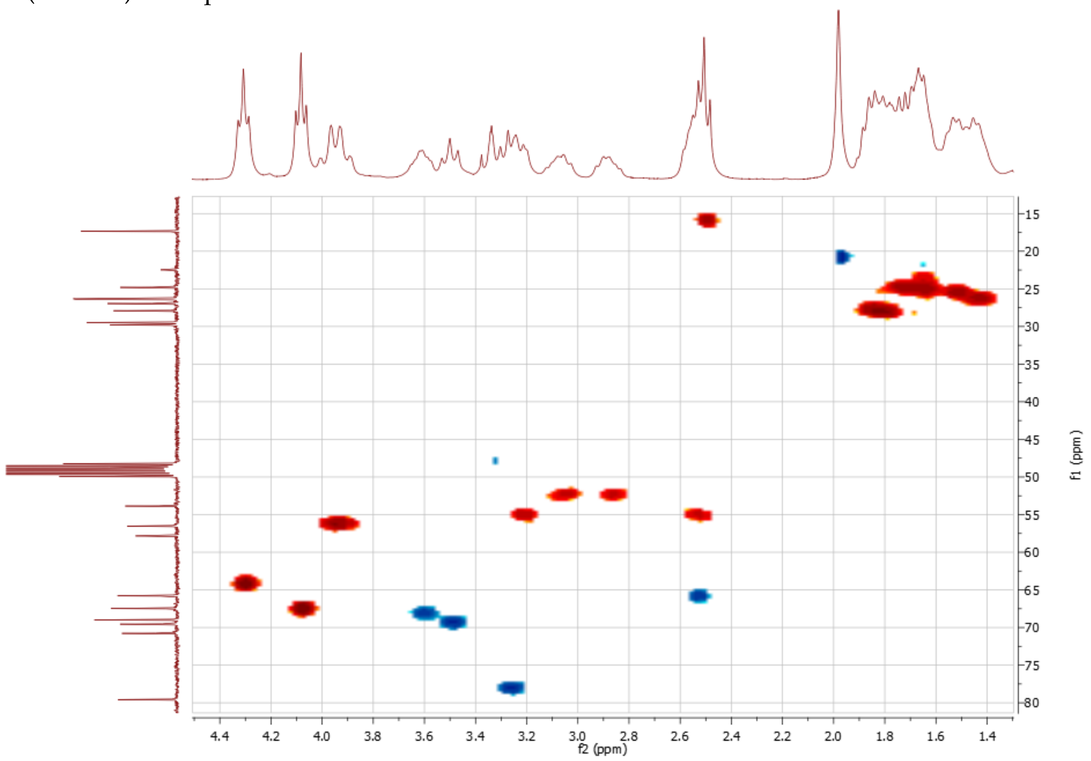

_N_-(6-((4-((5-Cyanopentyl)oxy)benzoyl)oxy)hexyl)-1,5-dideoxy-1,5-imino-D-xylitol (46)

^{1}H-NMR (300 MHz, CD_3OD): Compound **46**

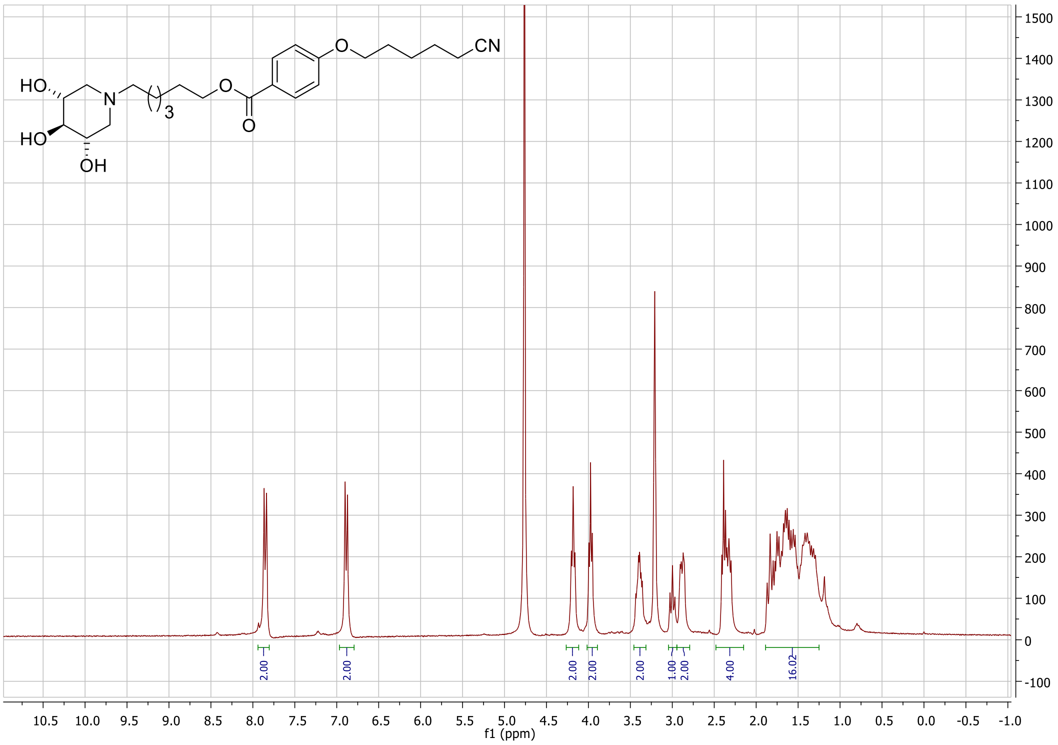

^{13}C-NMR (75.5 MHz, CD_3OD): Compound **46**

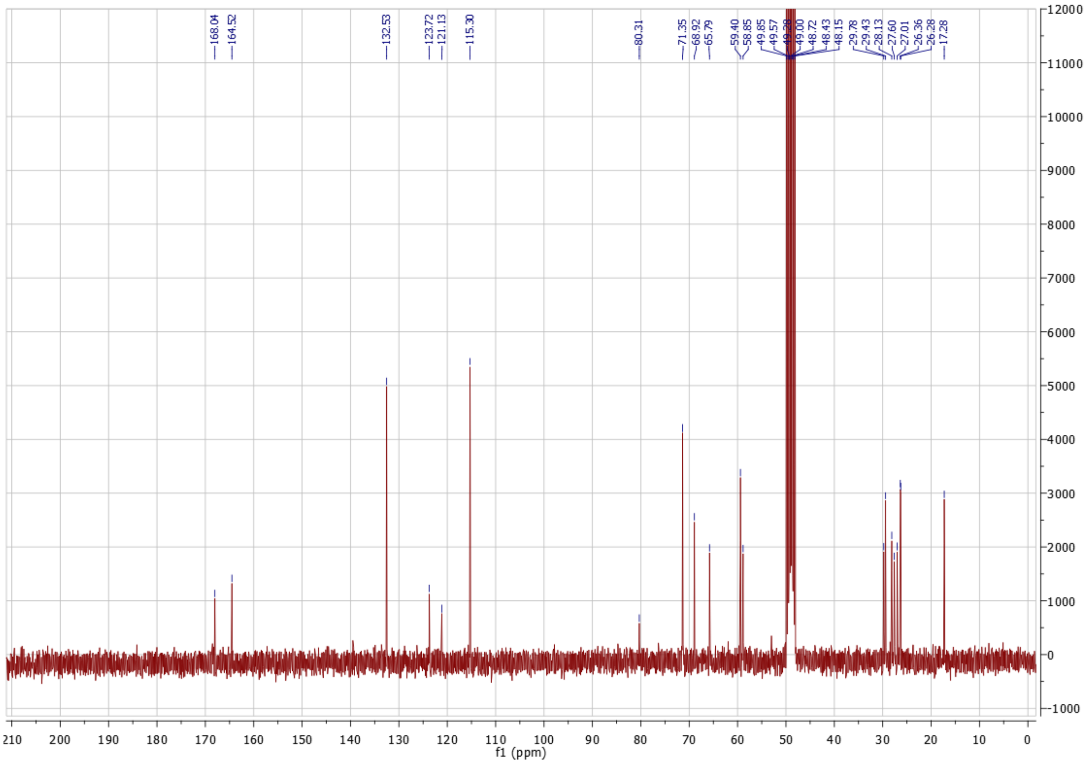

COSY (CD_3OD): Compound **46**

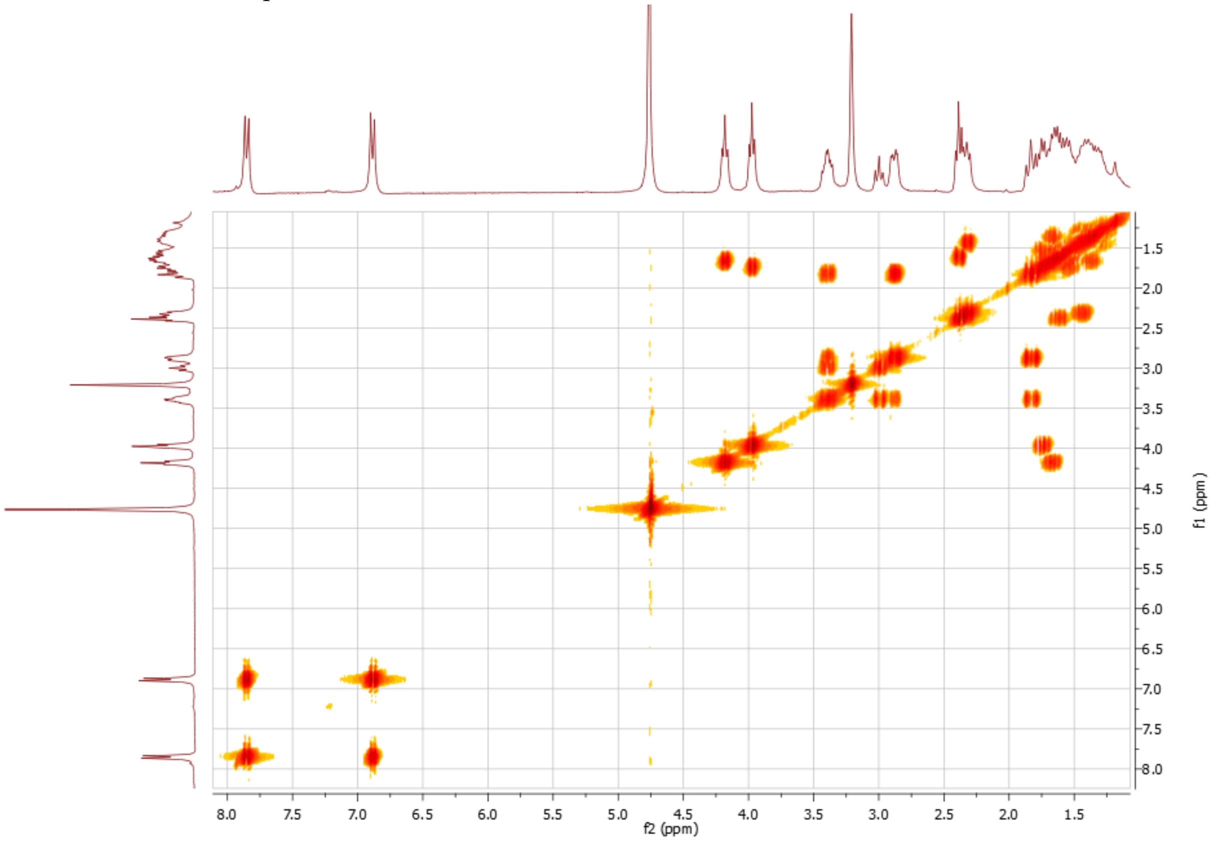

HSQC (CD_3OD): Compound **46**

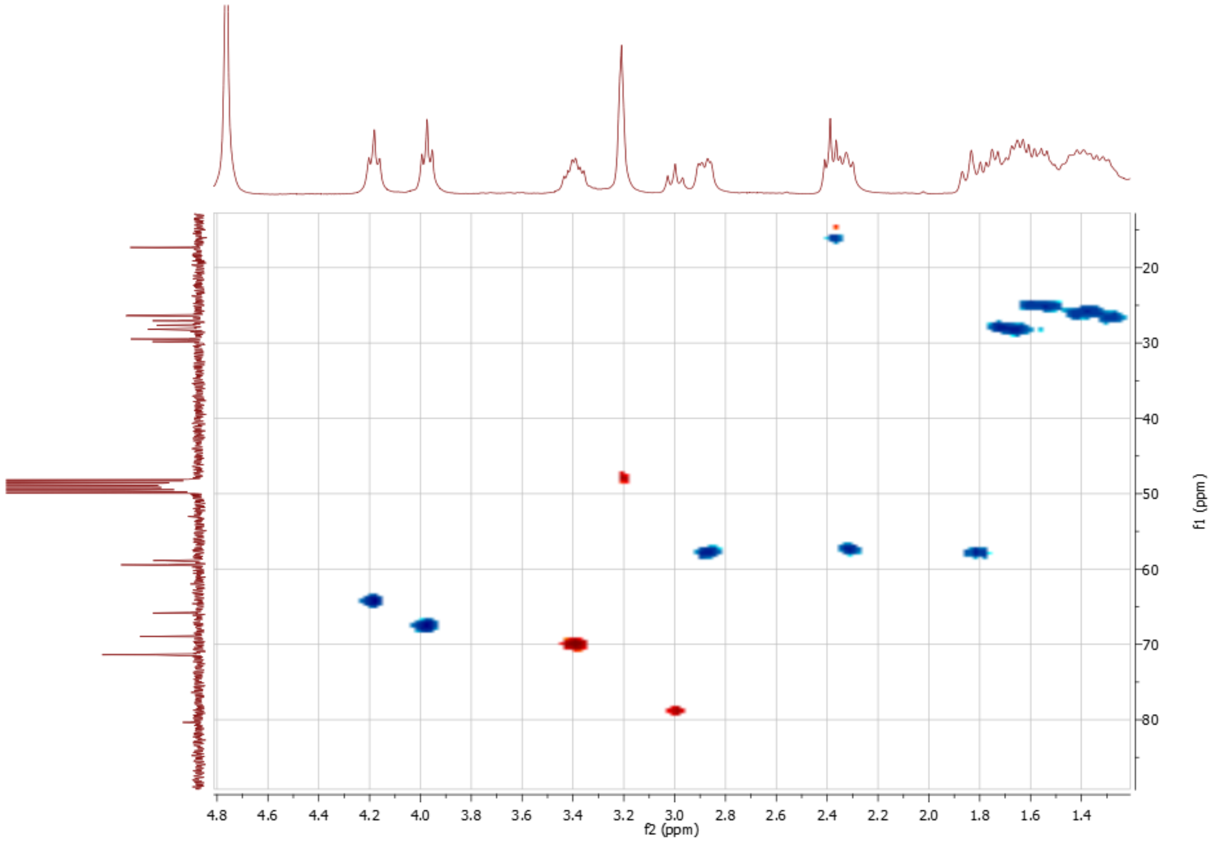

N-(6-((4-(2-(2-Azidoethoxy)ethoxy)benzoyl)oxy)hexyl)-1,5-dideoxy-1,5-imino-D-glucitol (47)

^{1}H-NMR (300 MHz, CD_3OD): Compound **47**

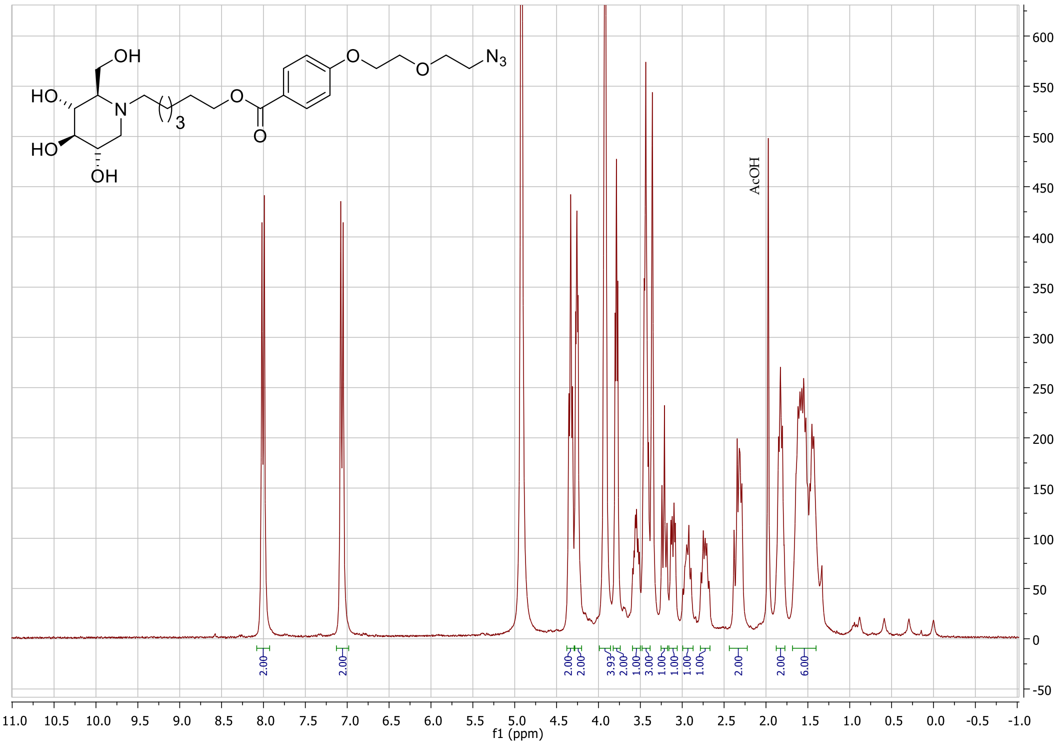

^{13}C-NMR (75.5 MHz, CD_3OD): Compound **47**

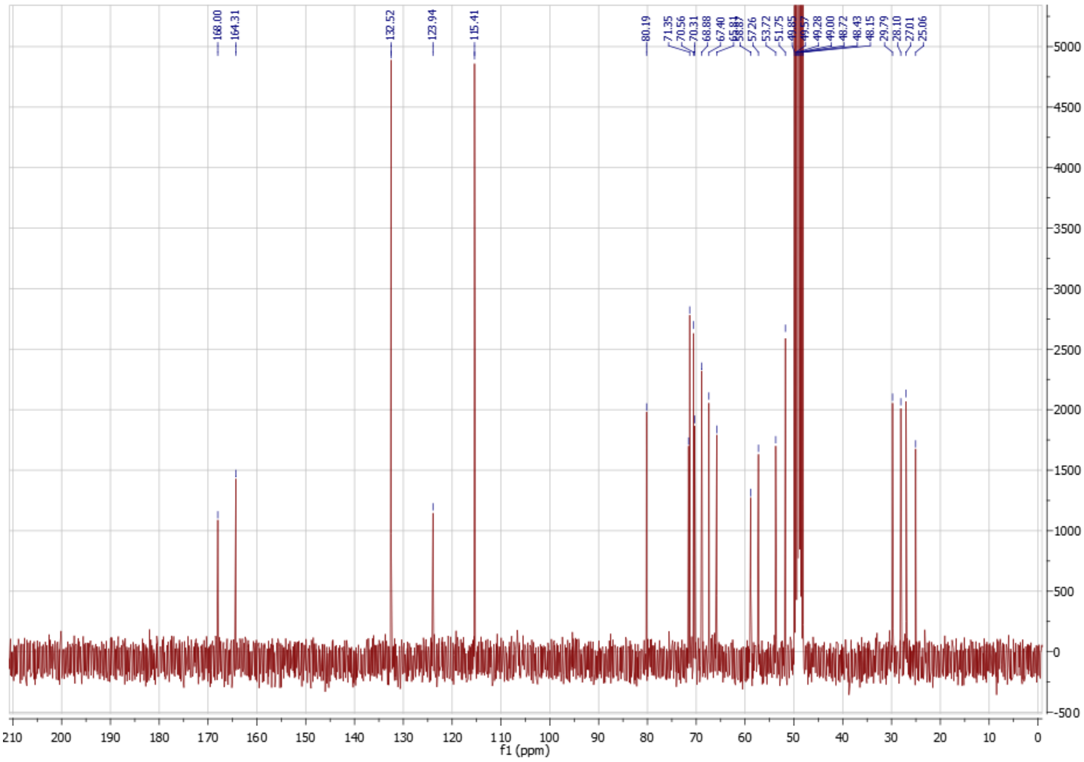

COSY (CD_3OD): Compound **47**

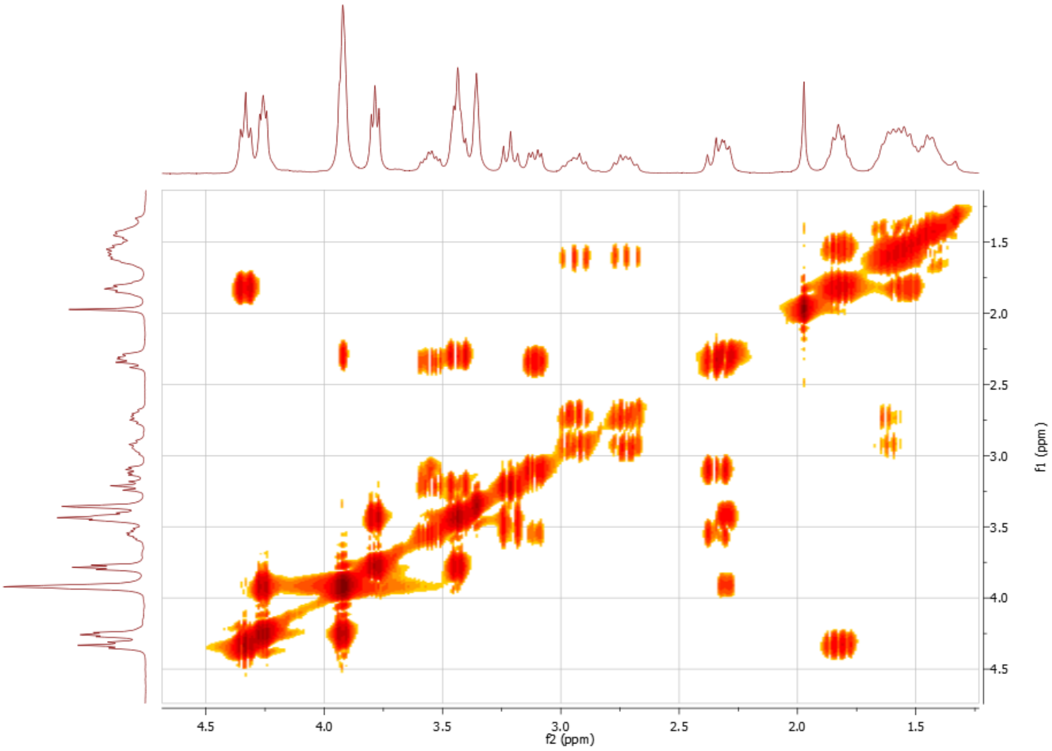

HSQC (CD_3OD): Compound **47**

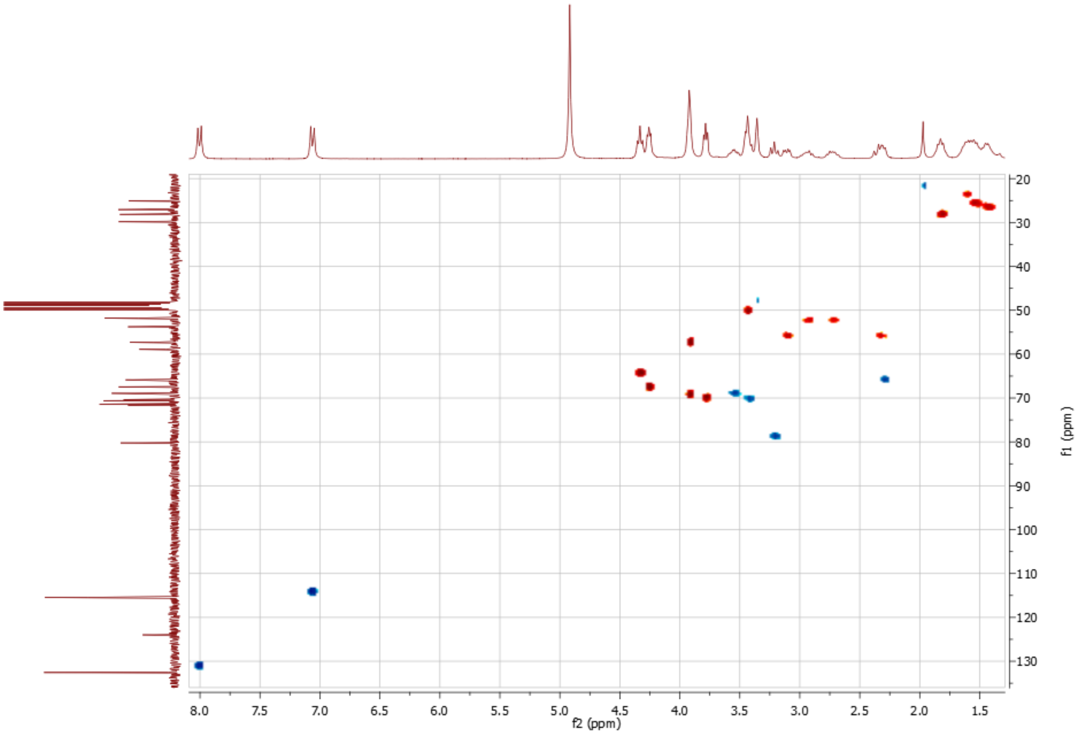

_N_-(6-((4-(2-(2-Azidoethoxy)ethoxy)benzoyl)oxy)hexyl)-1,5-dideoxy-1,5-imino-D-xylitol (48)

^{1}H-NMR (300 MHz, CD_3OD): Compound **48**

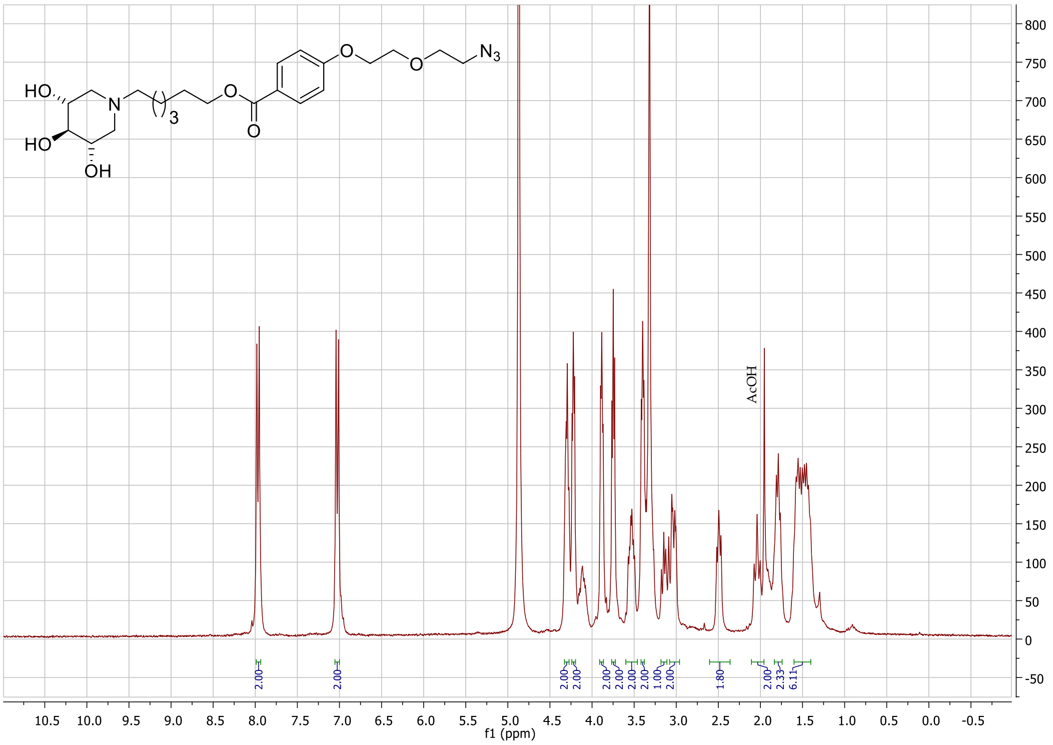

^{13}C NMR (75.5 MHz, CD_3OD): Compound **48**

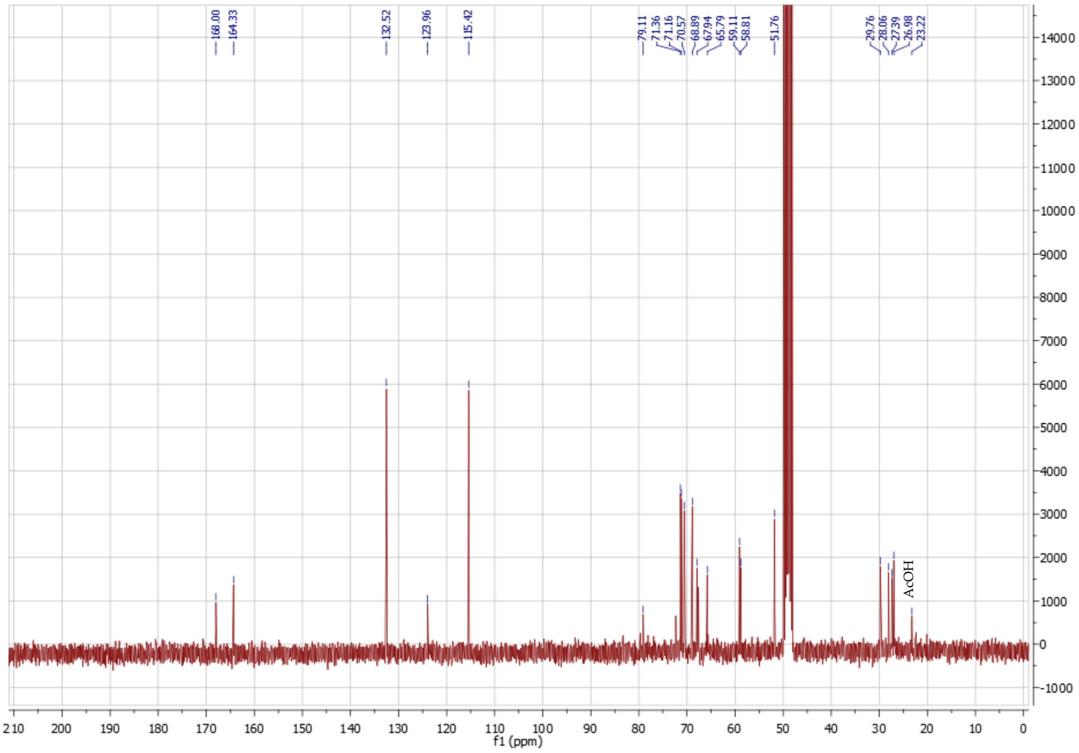

COSY (CD3OD): Compound **48**

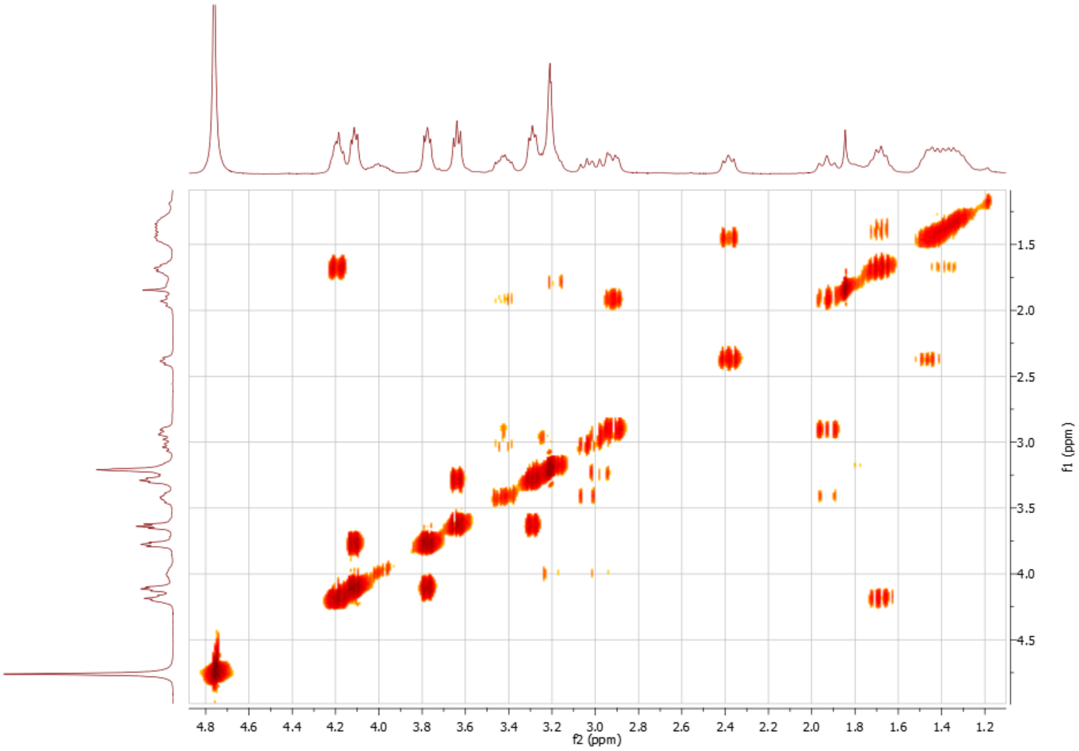

HSQC (CD3OD): Compound **48**

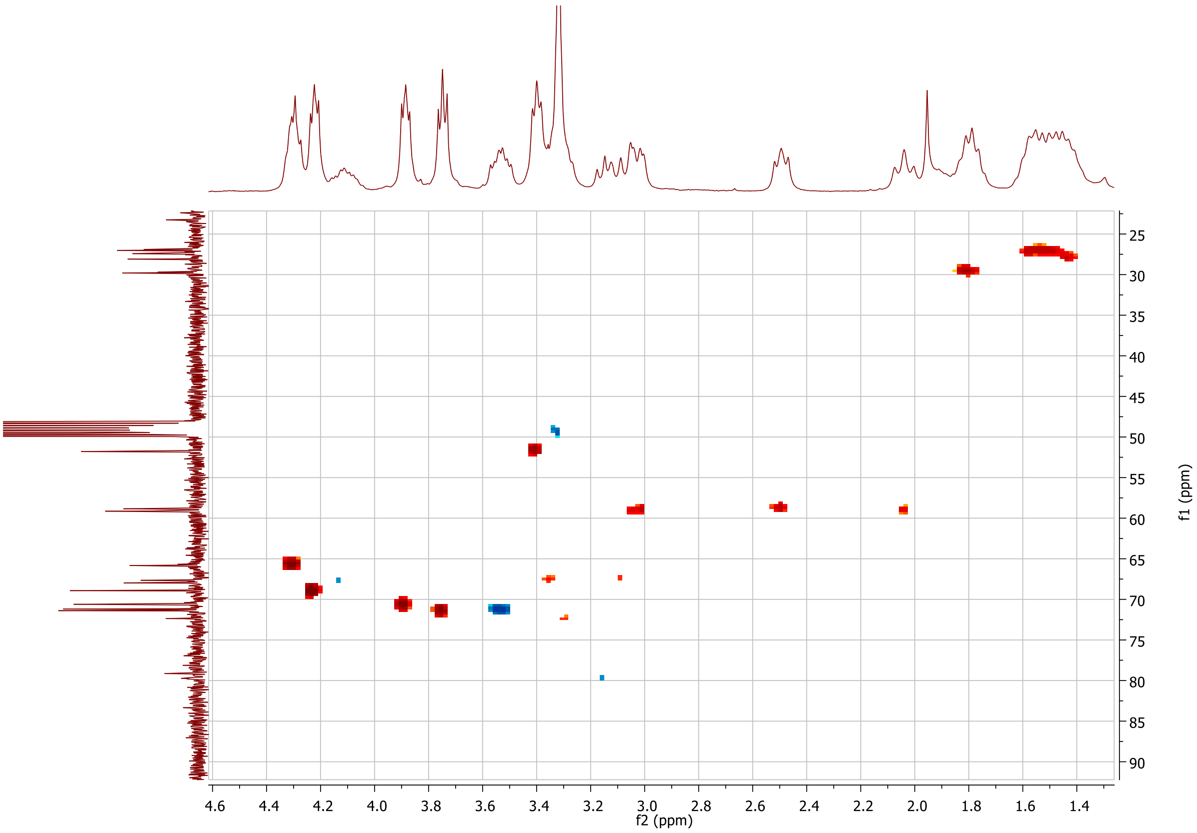

N-(6-((4-(2-(2-(Nonafluoro-*tert*-butyloxy)ethoxy)ethoxy)benzoyl)oxy)hexyl)-1,5-dideoxy-1,5-imino-D-glucitol (49)

^{1}H-NMR (300 MHz, CD3OD): Compound **49**

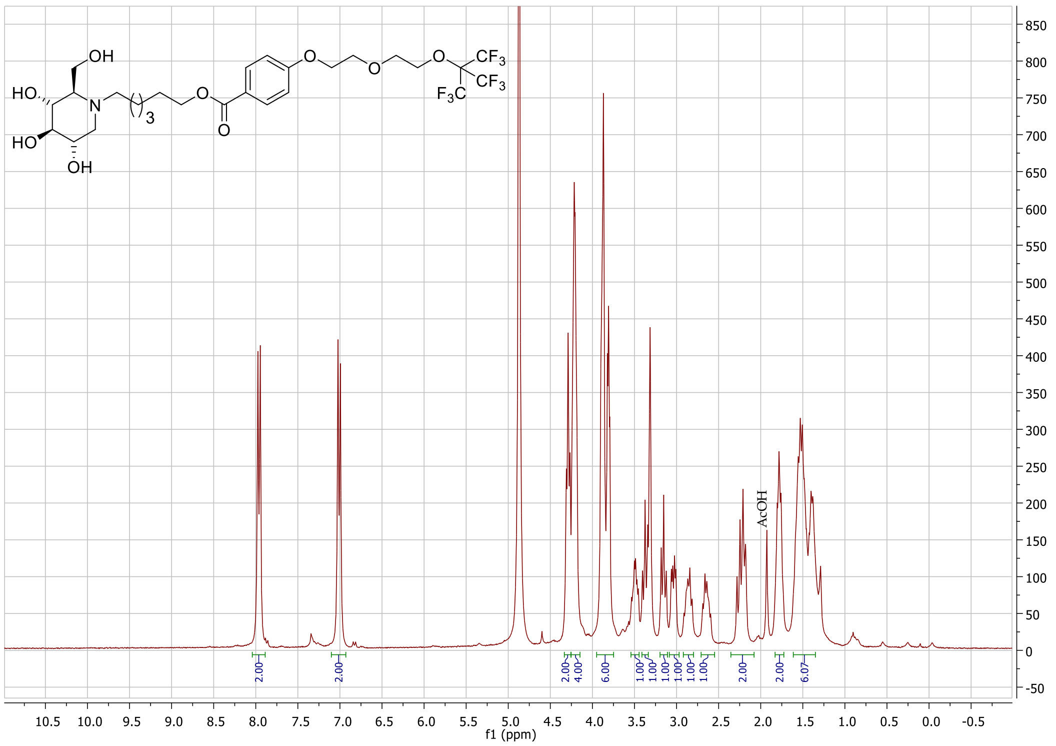

^{13}C-NMR (75.5 MHz, CD3OD): Compound **49**

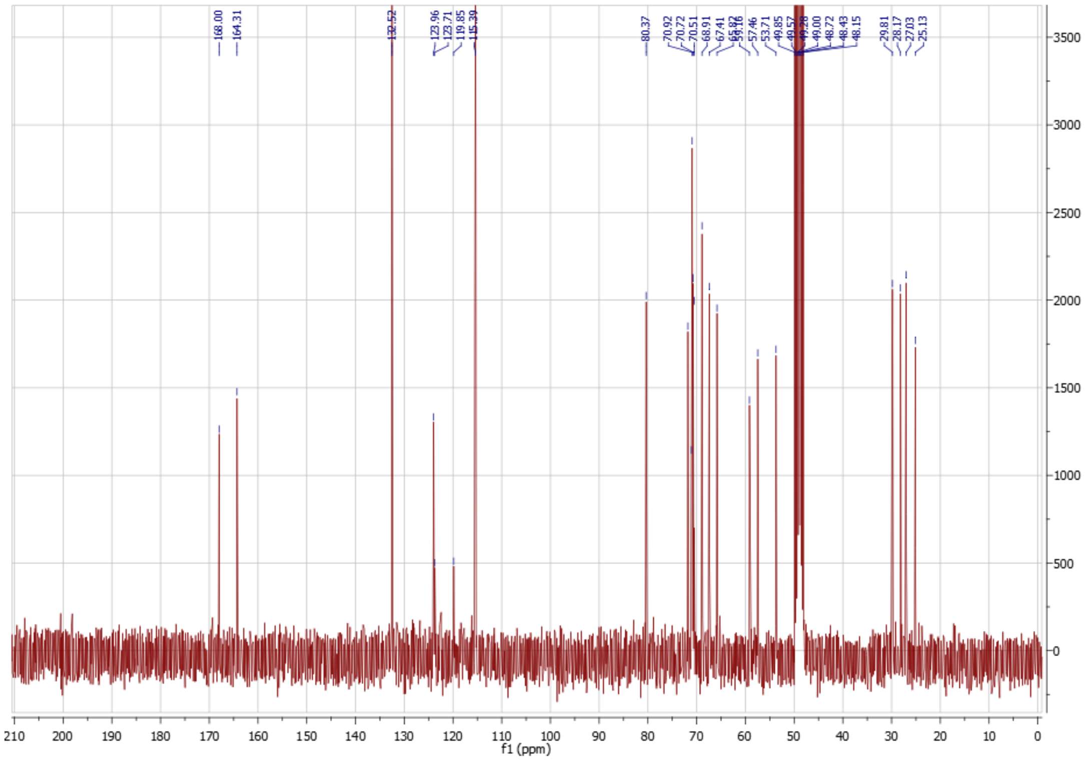

COSY (CD$_3$OD): Compound **49**

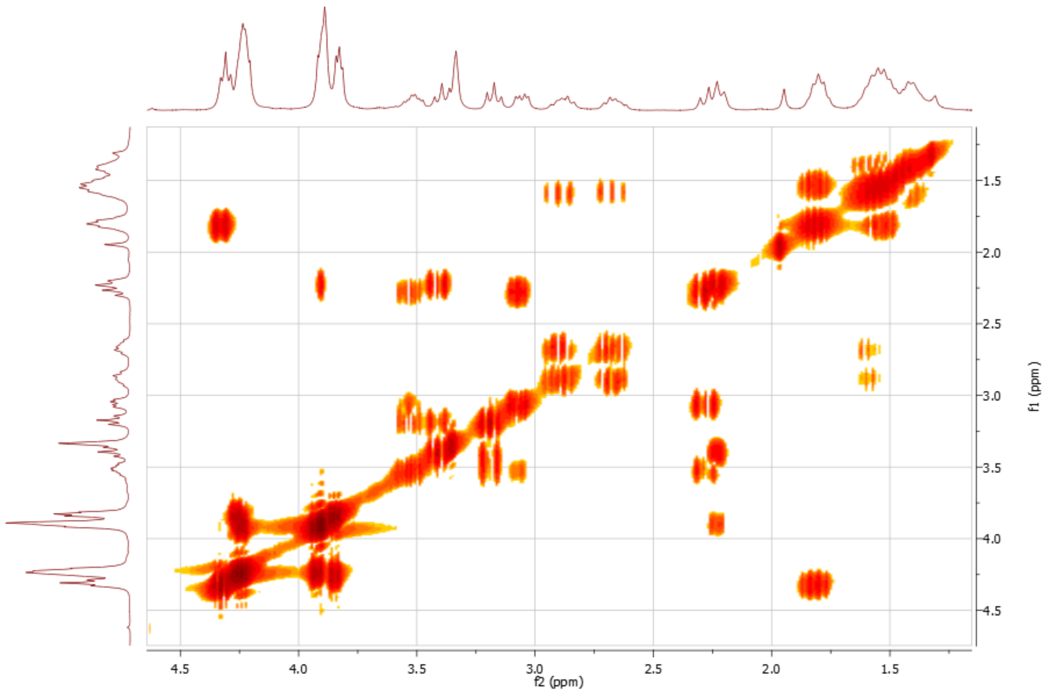

HSQC (CD$_3$OD): Compound **49**

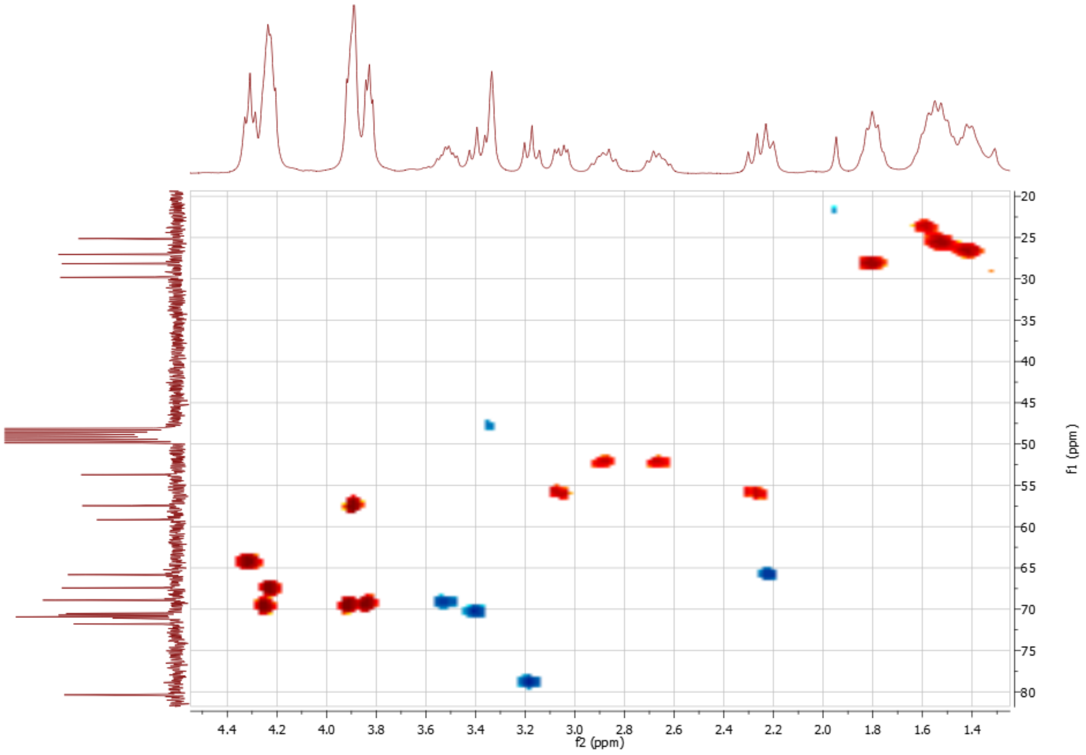

^{19}F-NMR (470.3 MHz, $CDCl_3$): Compound **49**

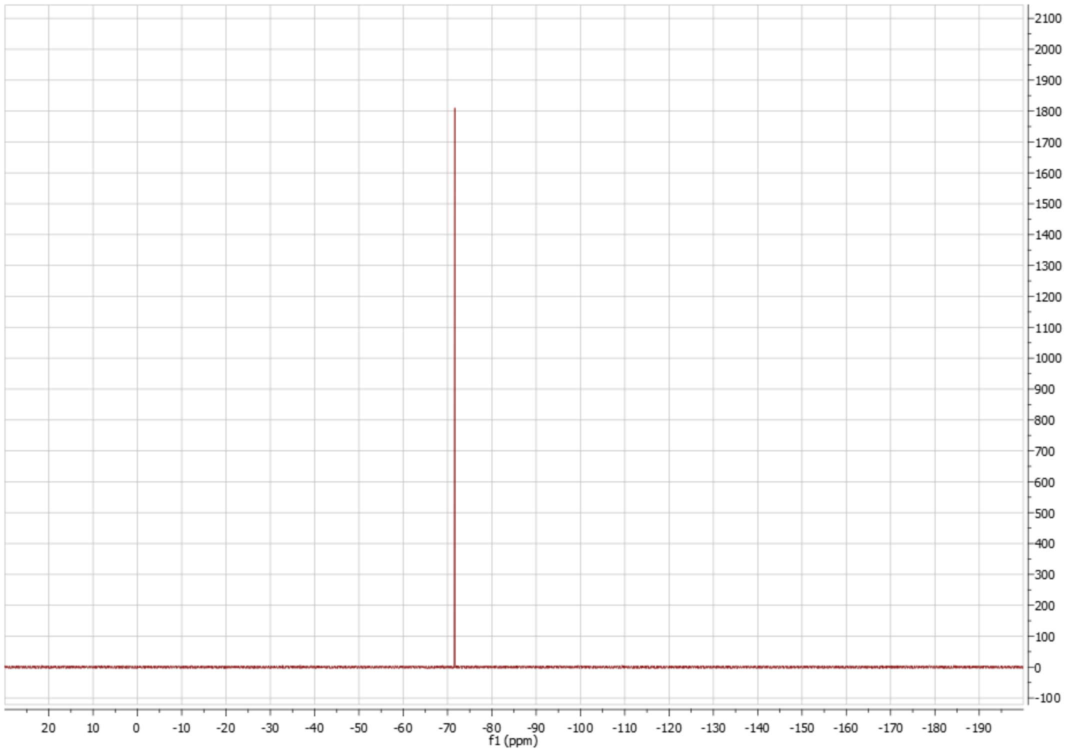

N-(6-((4-(2-(2-(Nonafluoro-*tert*-butyloxy)ethoxy)ethoxy)benzoyl)oxy)hexyl)-1,5-dideoxy-1,5-imino-D-xylitol (50)

^{1}H-NMR (300 MHz, CD_3OD): Compound **50**

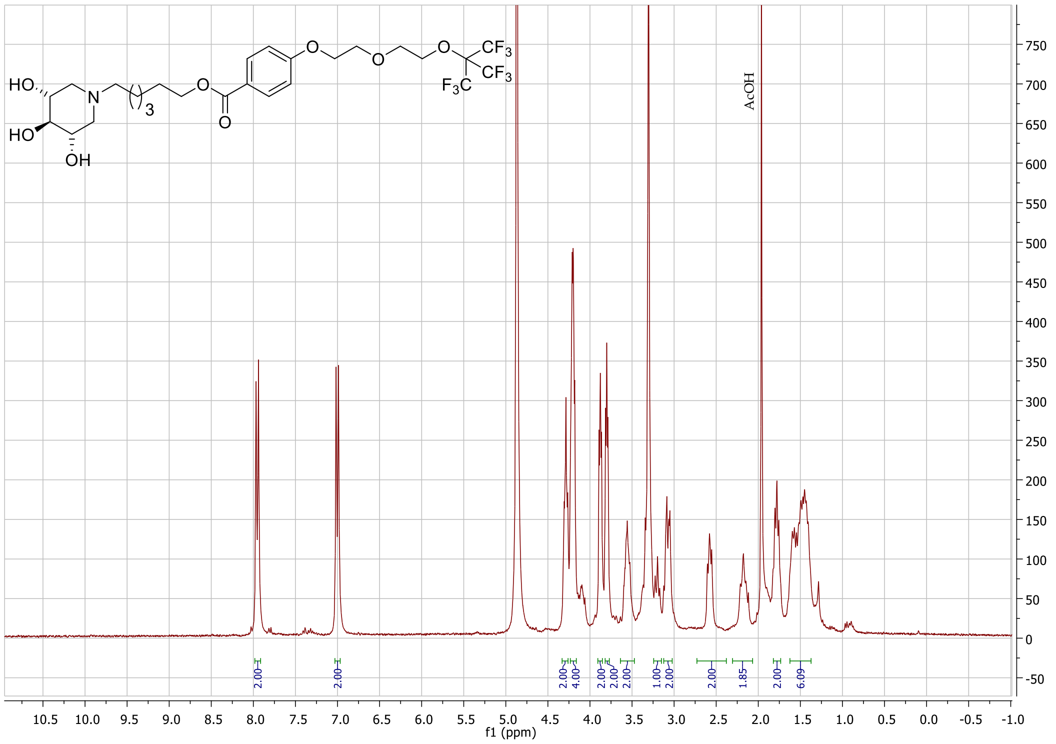

^{13}C-NMR (75.5 MHz, CD_3OD): Compound **50**

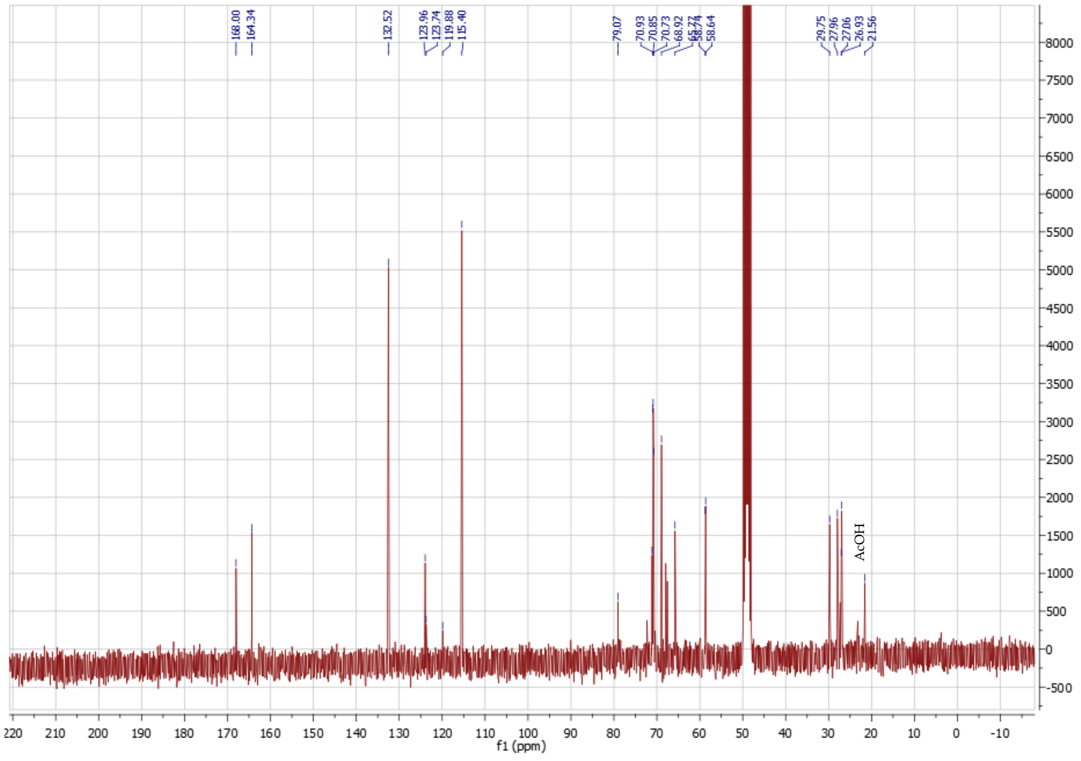

COSY (CD_3OD): Compound **50**

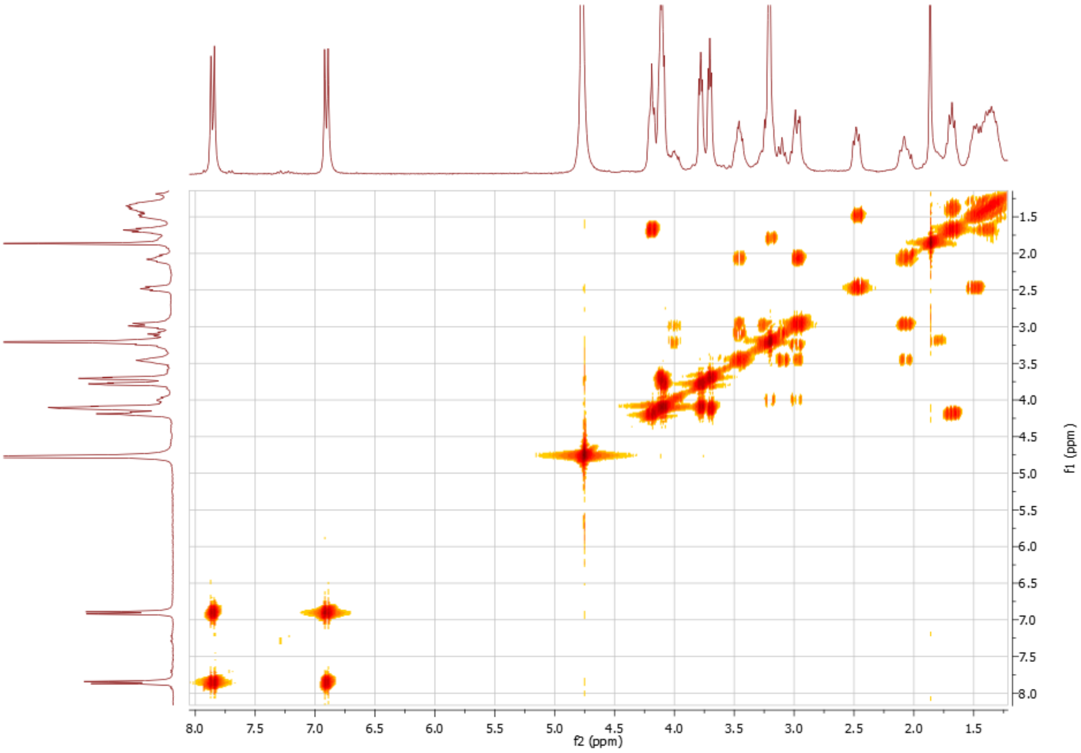

HSQC (CD_3OD): Compound **50**

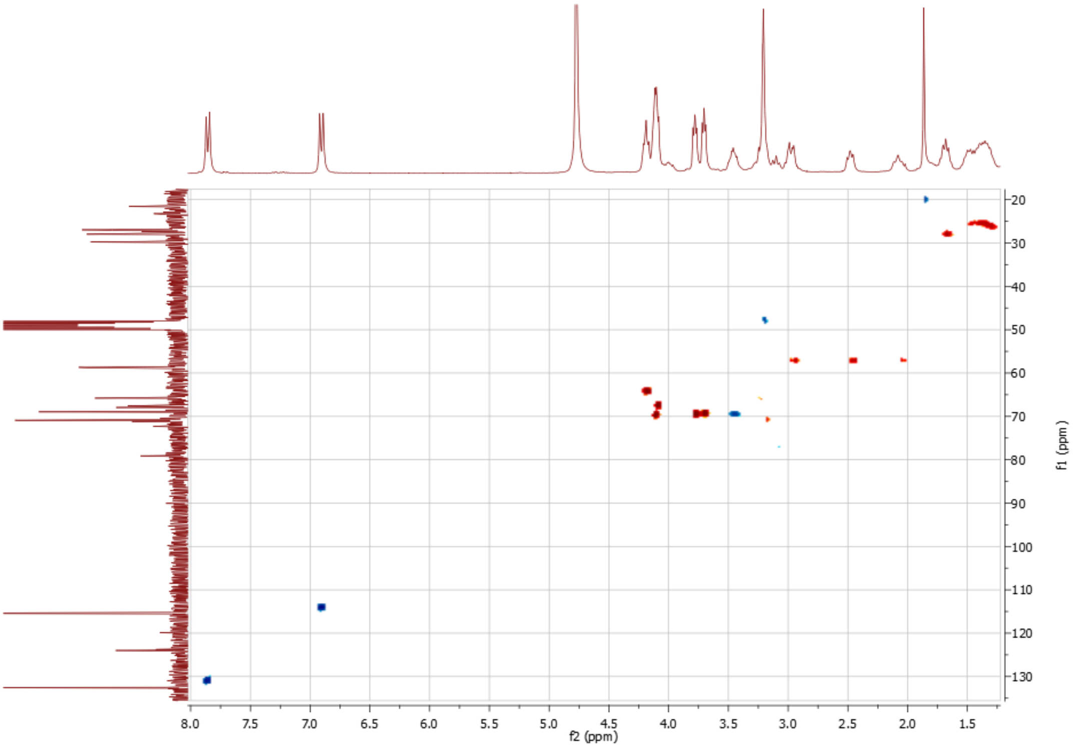

***N*-(6-((4-(Propargyloxy)benzoyl)oxy)hexyl)-1,5-dideoxy-1,5-imino-D-glucitol (51)**

[1]**H-NMR** (300 MHz, CD_3OD): Compound **51**

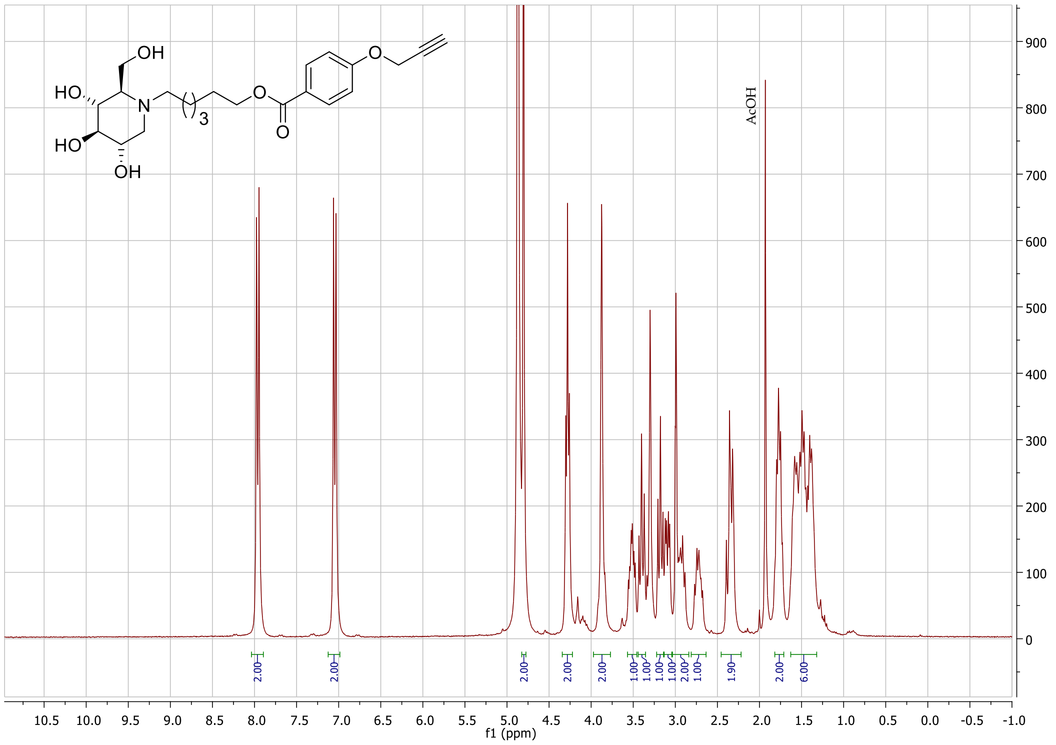

[13]**C NMR** (75.5 MHz, CD_3OD): Compound **51**

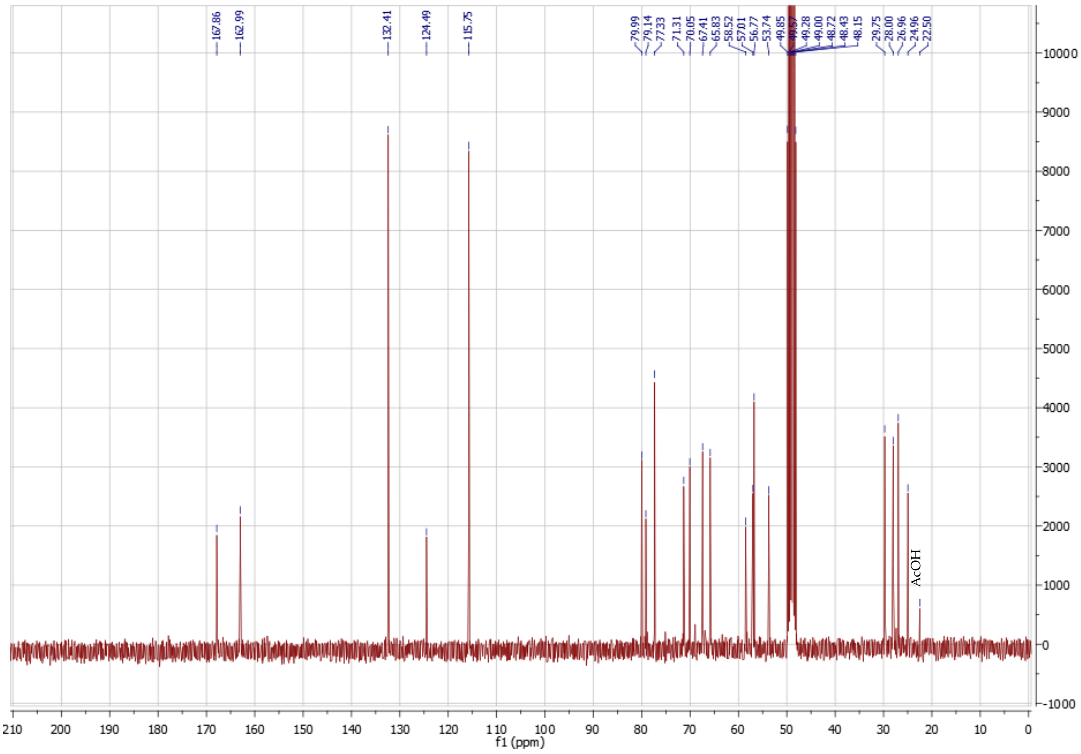

COSY (CD_3OD): Compound **51**

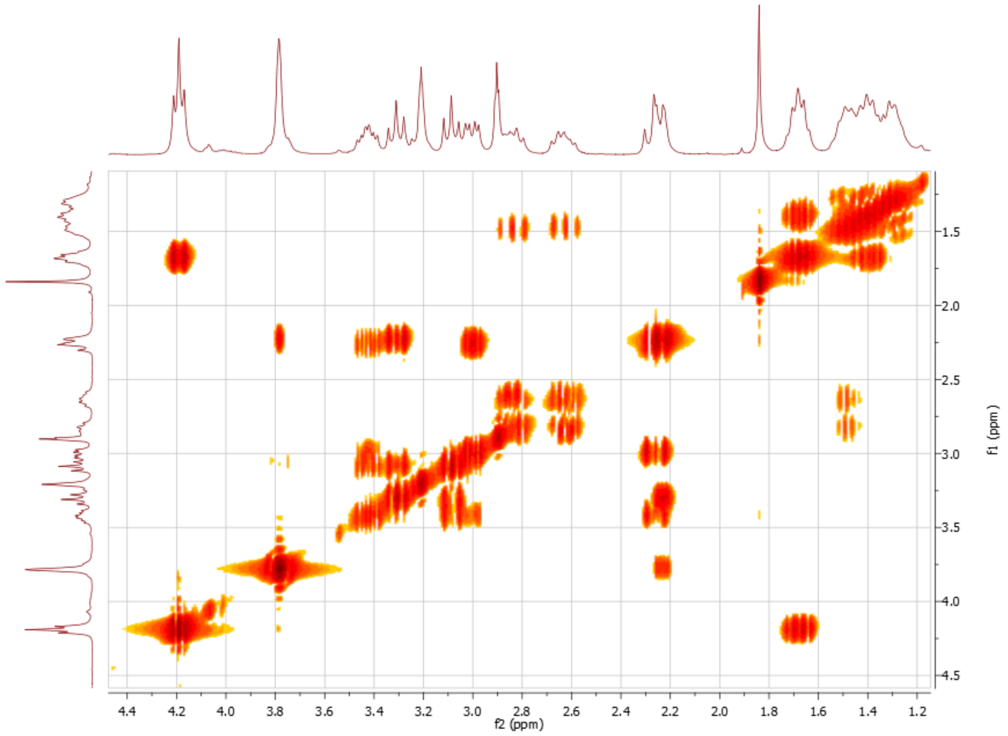

HSQC (CD_3OD): Compound **51**

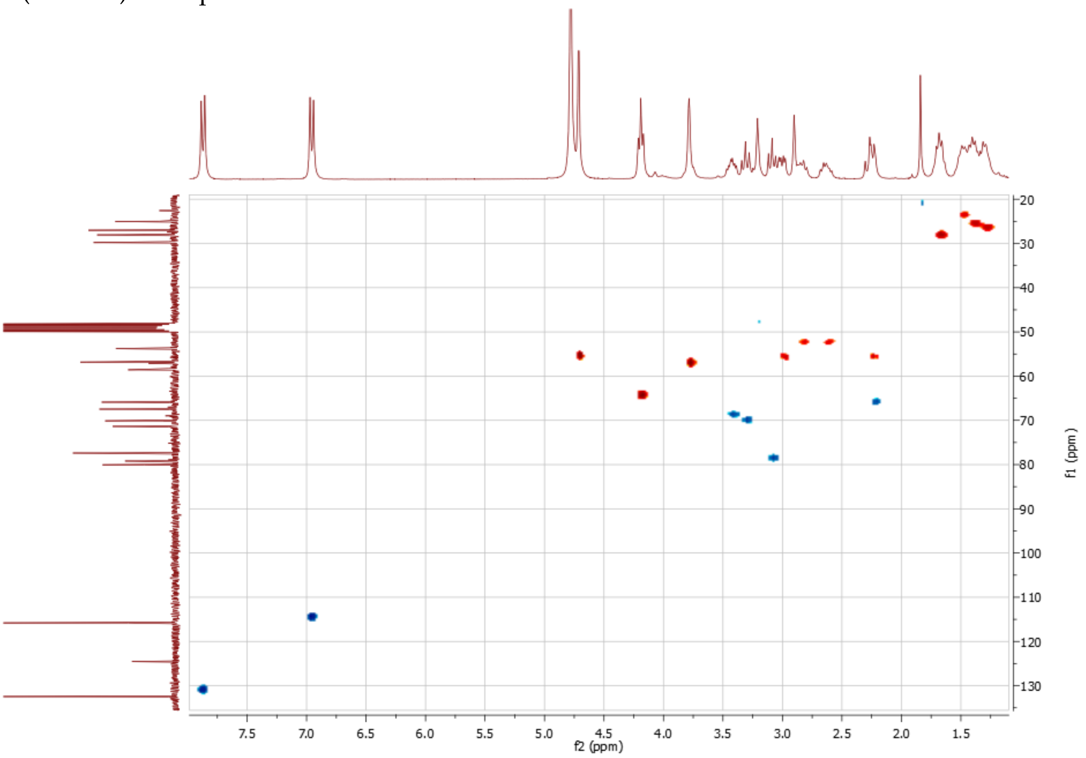

N-(6-((4-(Propargyloxy)benzoyl)oxy)hexyl)-1,5-dideoxy-1,5-imino-D-xylitol (52)

^{1}H-NMR (300 MHz, CD_3OD): Compound **52**

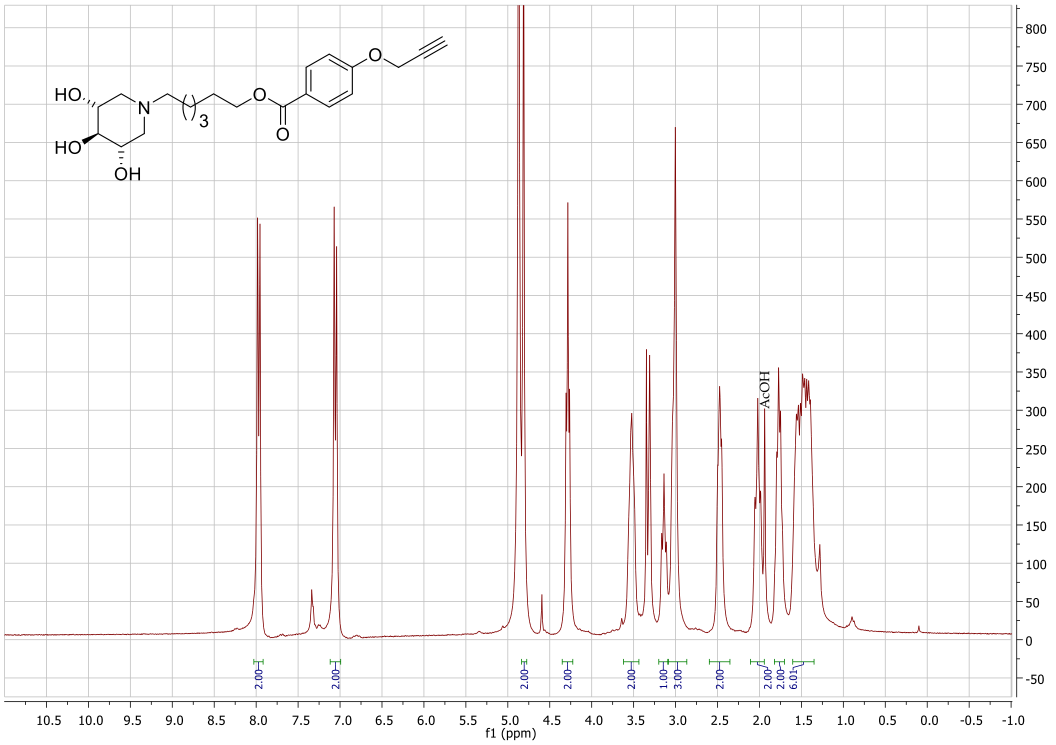

^{13}C-NMR (75.5 MHz, CD_3OD): Compound **52**

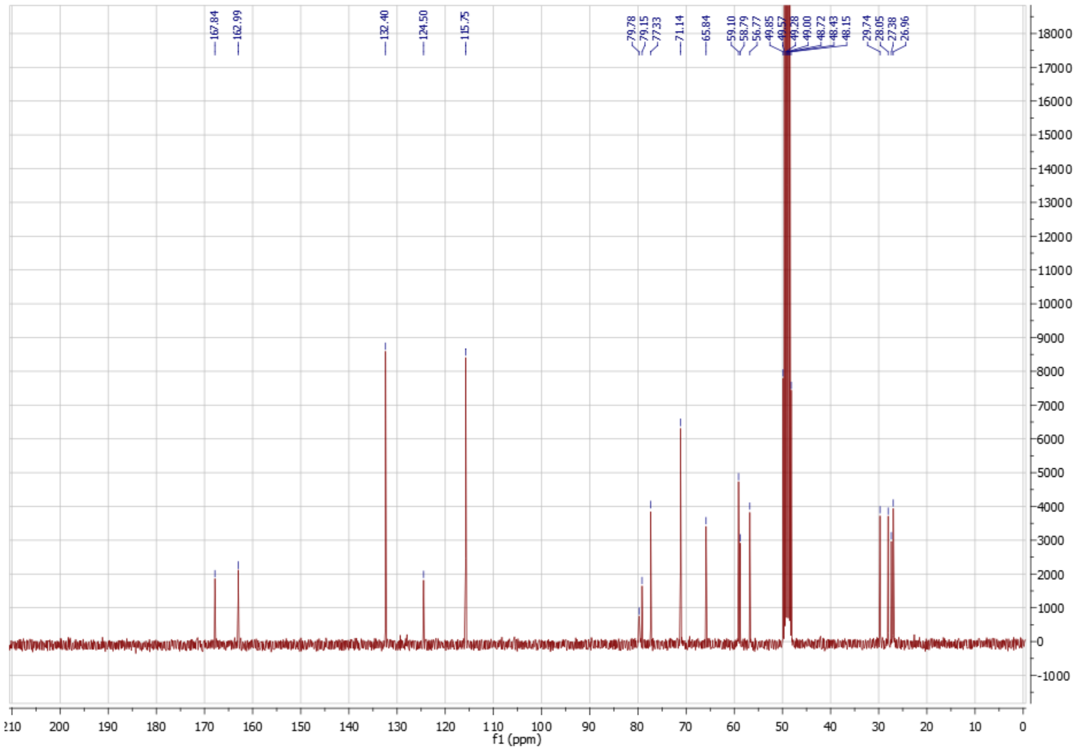

COSY (CD_3OD): Compound **52**

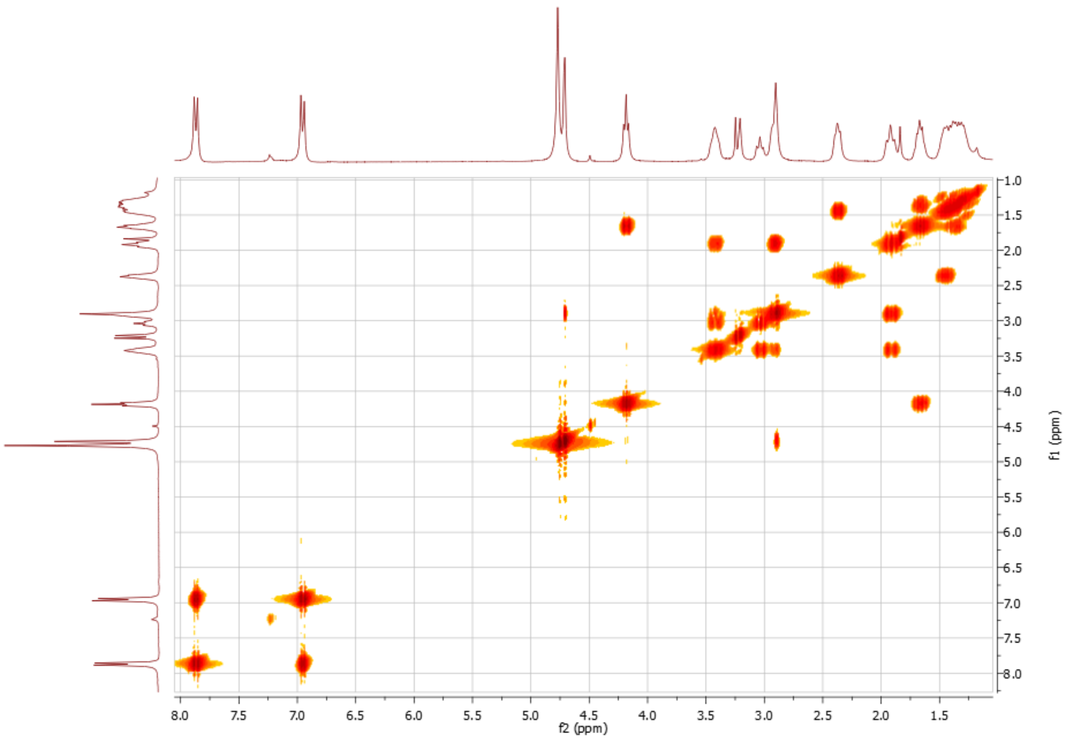

HSQC (CD_3OD): Compound **52**

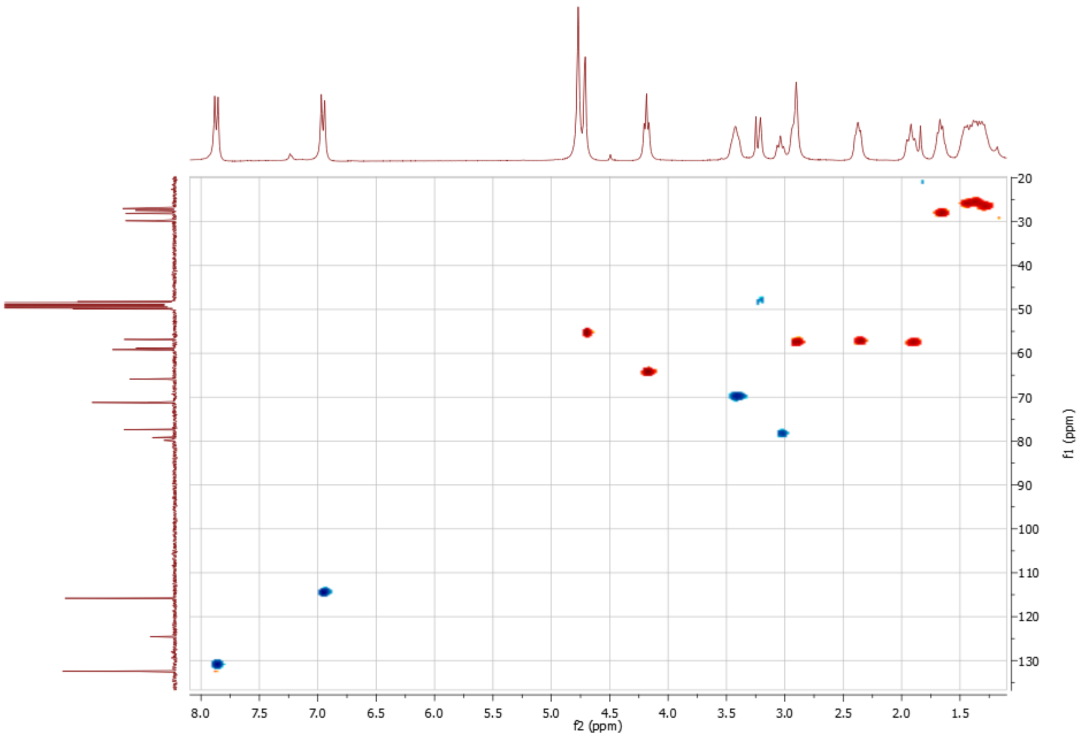

_N_-(6-((4-(2-(((Benzyloxy)carbonyl)amino)ethyl)benzoyl)oxy)hexyl)-1,5-dideoxy-1,5-imino-D-glucitol (53)

[1]**H-NMR** (300 MHz, CD_3OD): Compound **53**

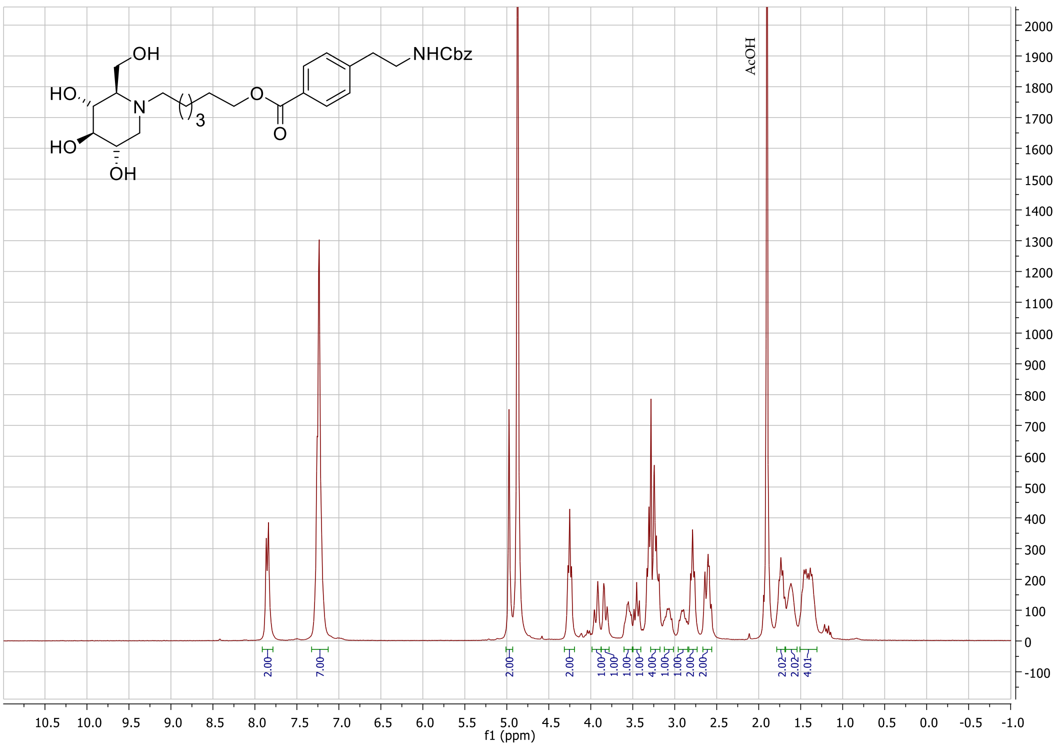

[13]**C-NMR** (75.5 MHz, CD_3OD): Compound **53**

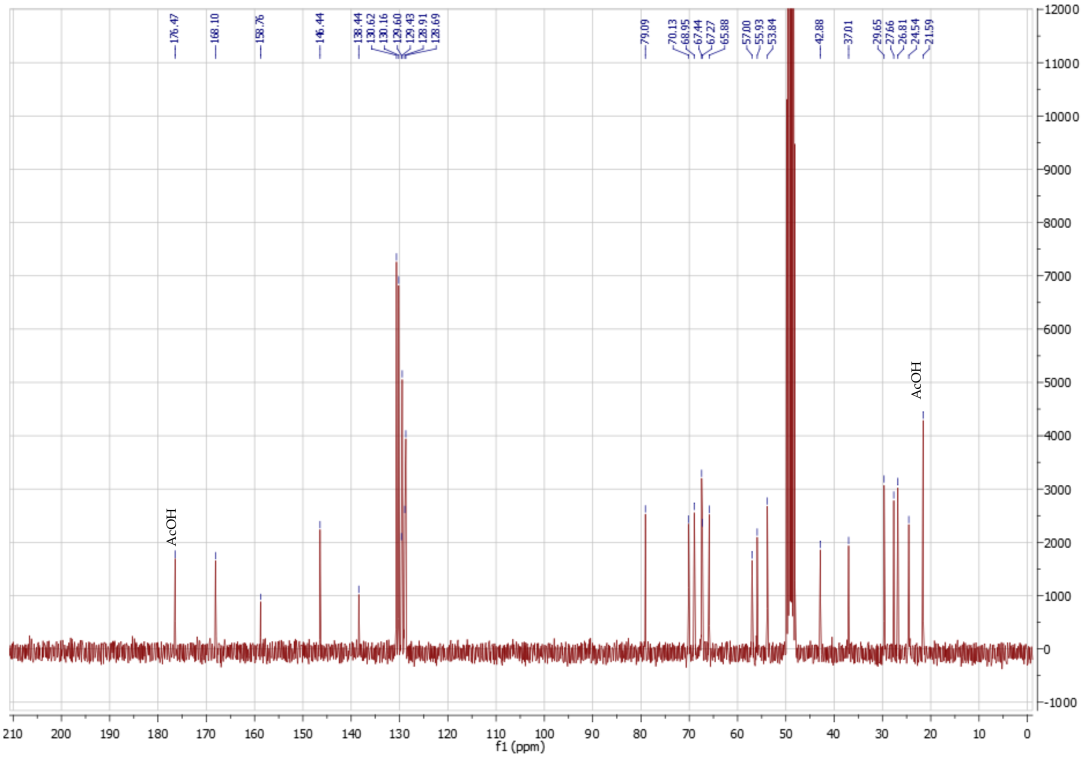

COSY (CD3OD): Compound **53**

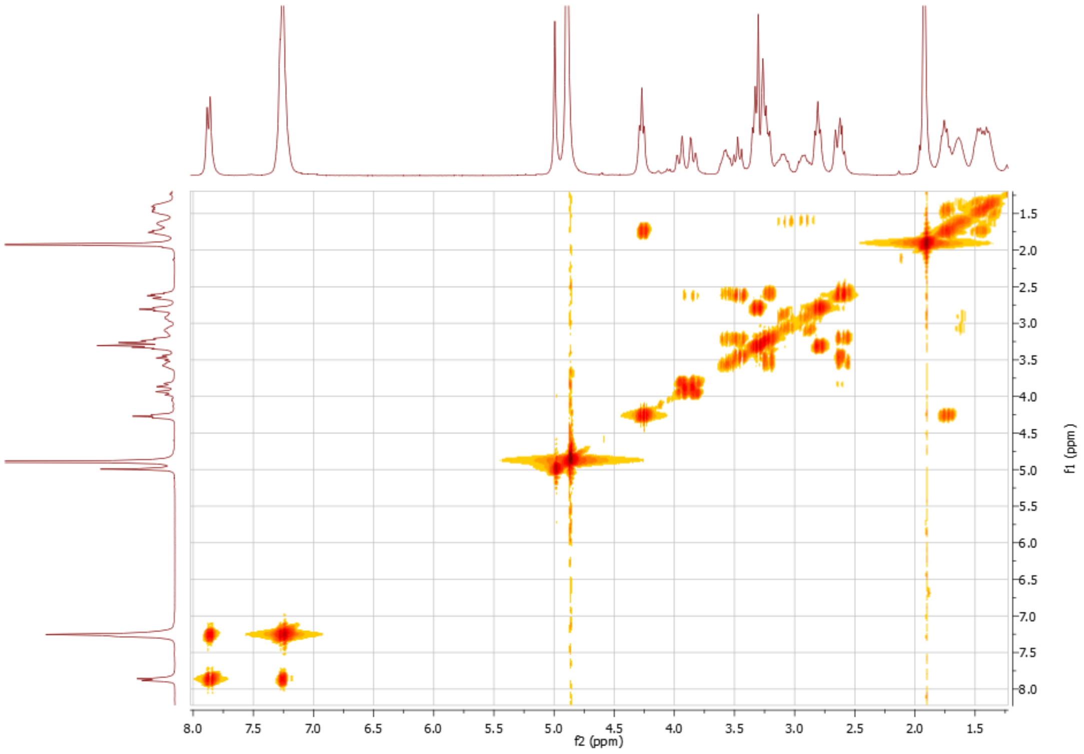

HSQC (CD3OD): Compound **53**

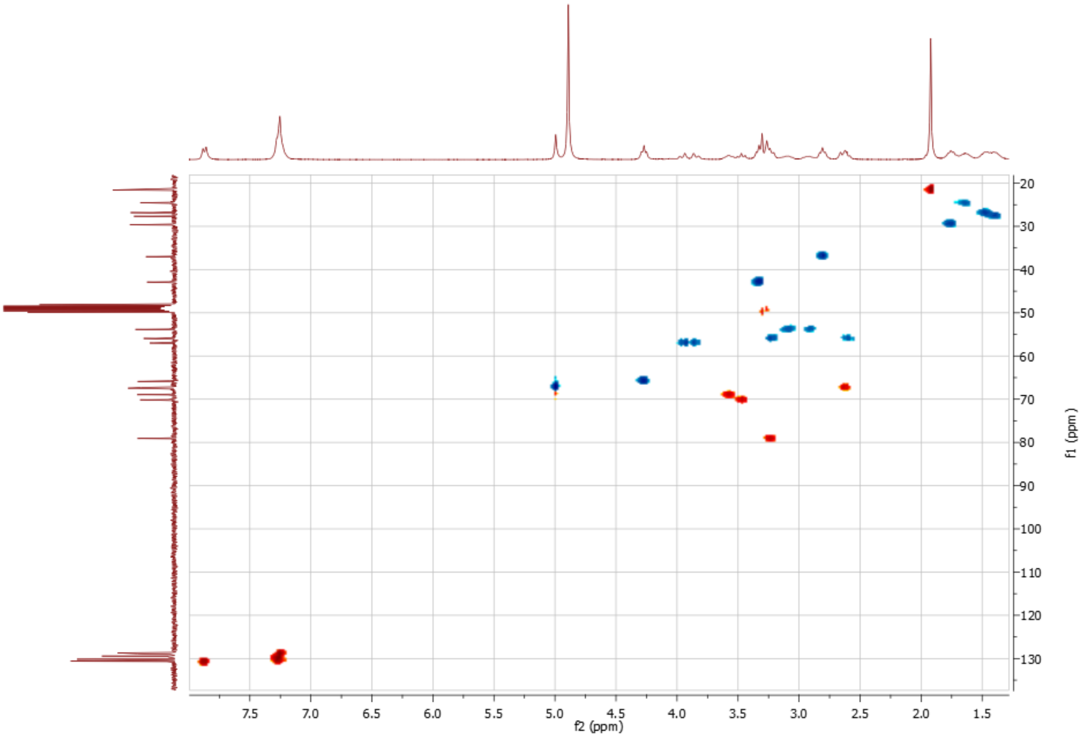

***N*-(6-((4-(2-(((Benzyloxy)carbonyl)amino)ethyl)benzoyl)oxy)hexyl)-1,5-dideoxy-1,5-imino-D-xylitol (54)**

[1]**H-NMR** (300 MHz, CD_3OD): Compound **54**

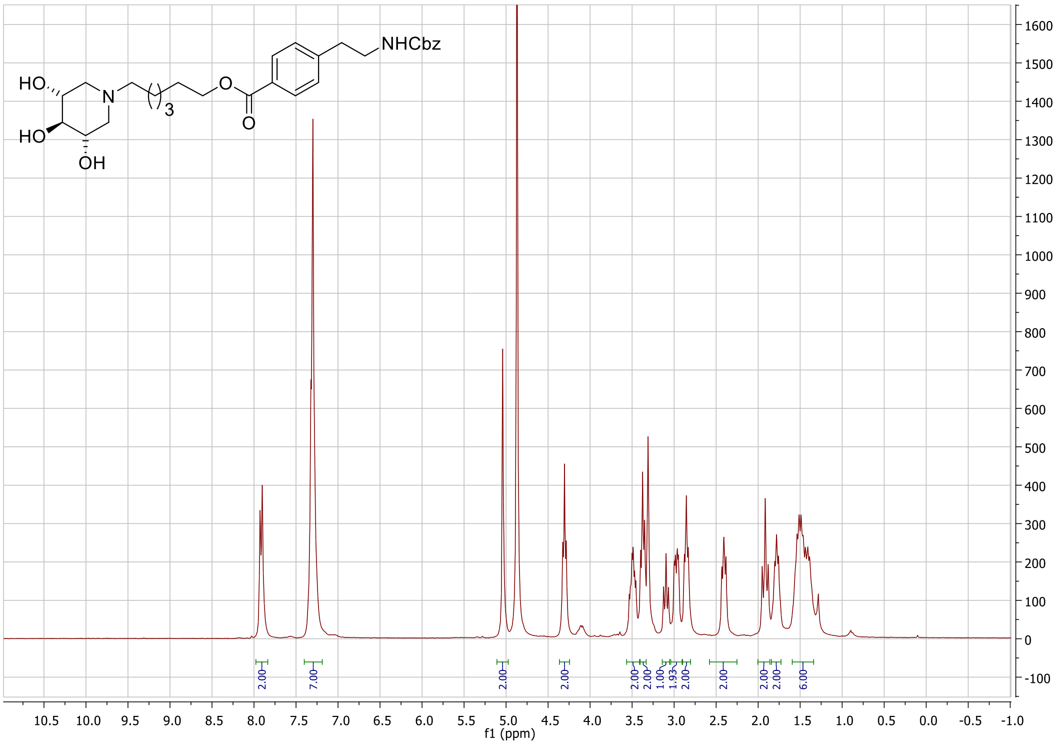

[13]**C NMR** (75.5 MHz, CD_3OD): Compound **54**

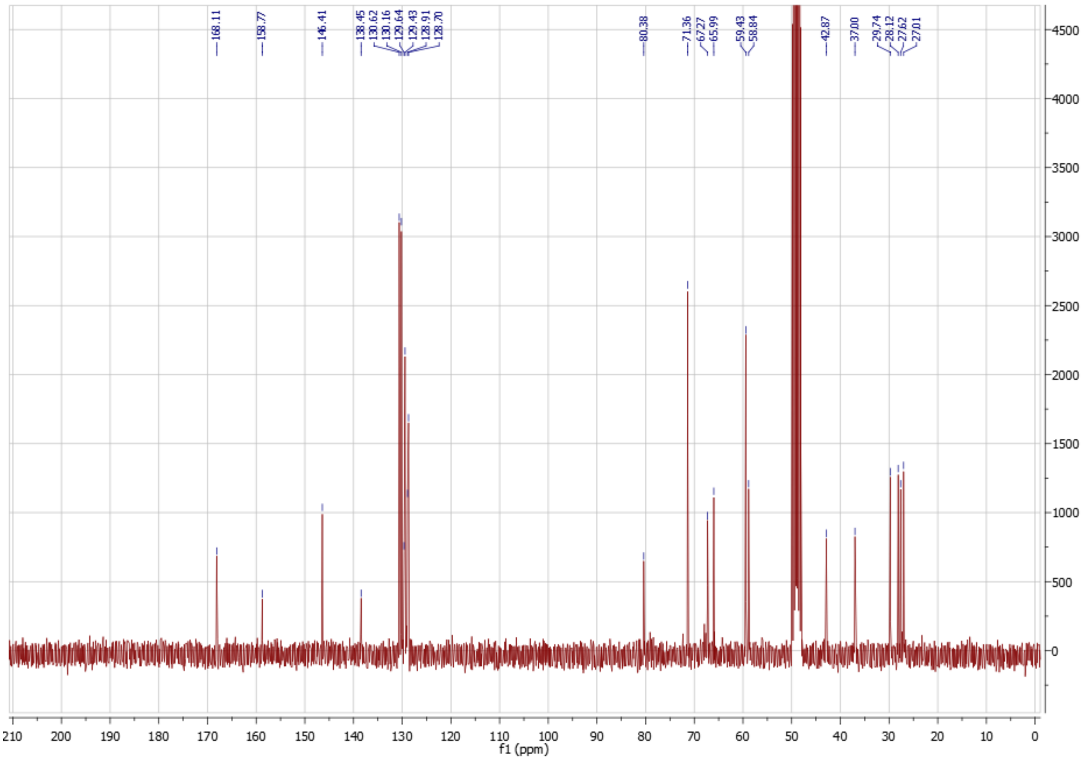

COSY (CD_3OD): Compound **54**

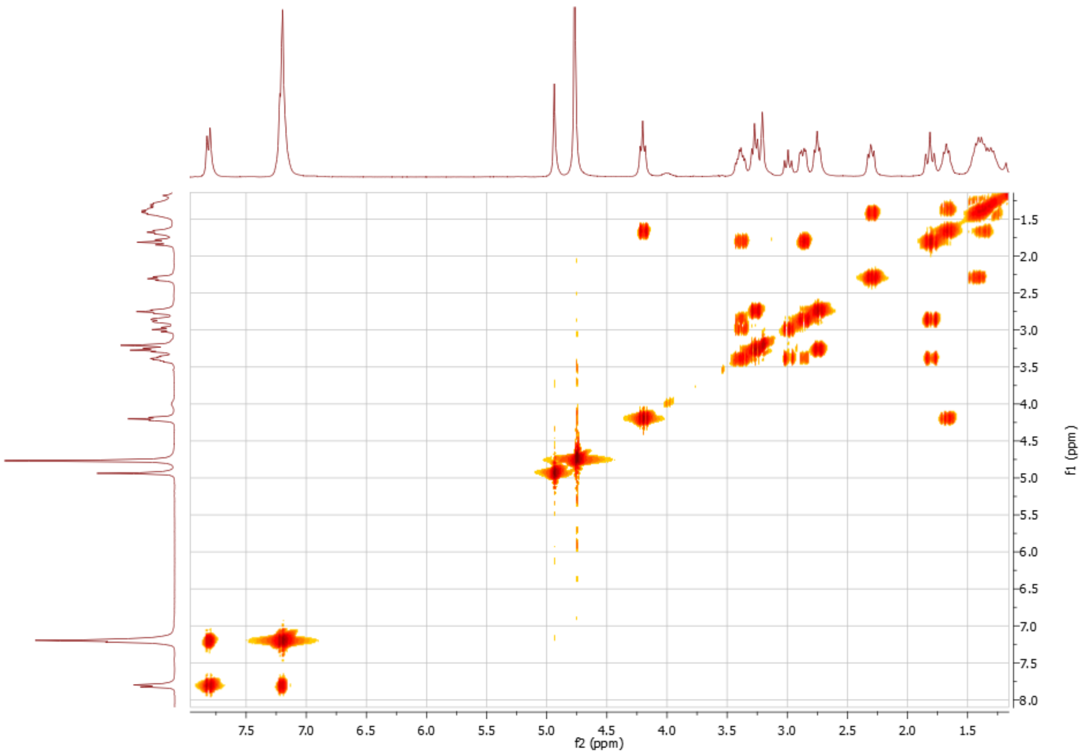

HSQC (CD_3OD): Compound **54**

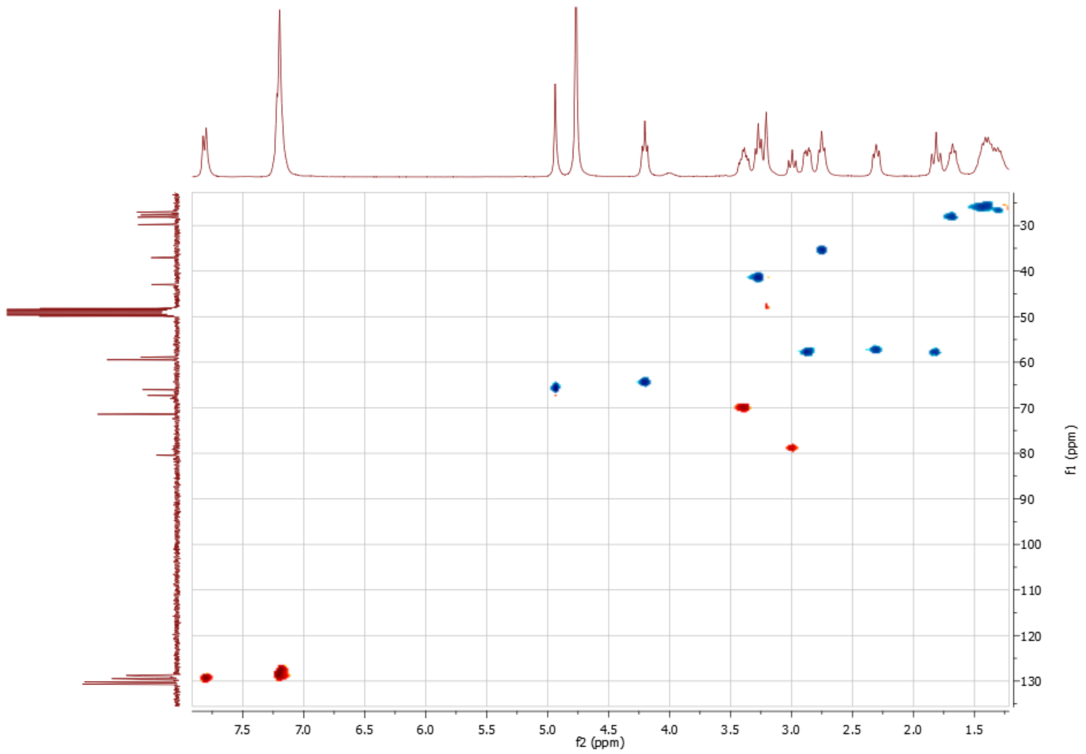

N-(6-((4-(2-(Dansylamino)ethyl)benzoyl)oxy)hexyl)-1,5-dideoxy-1,5-imino-D-glucitol (55)

¹H-NMR (300 MHz, CD_3OD): Compound **55**

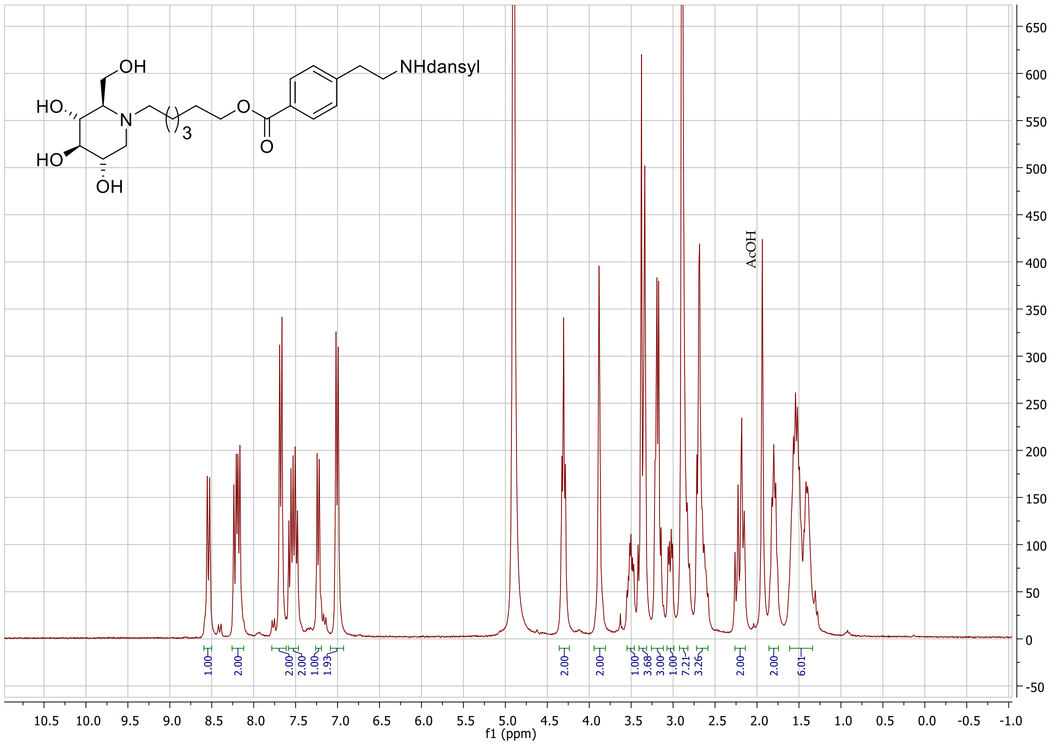

¹³C-NMR (75.5 MHz, CD_3OD): Compound **55**

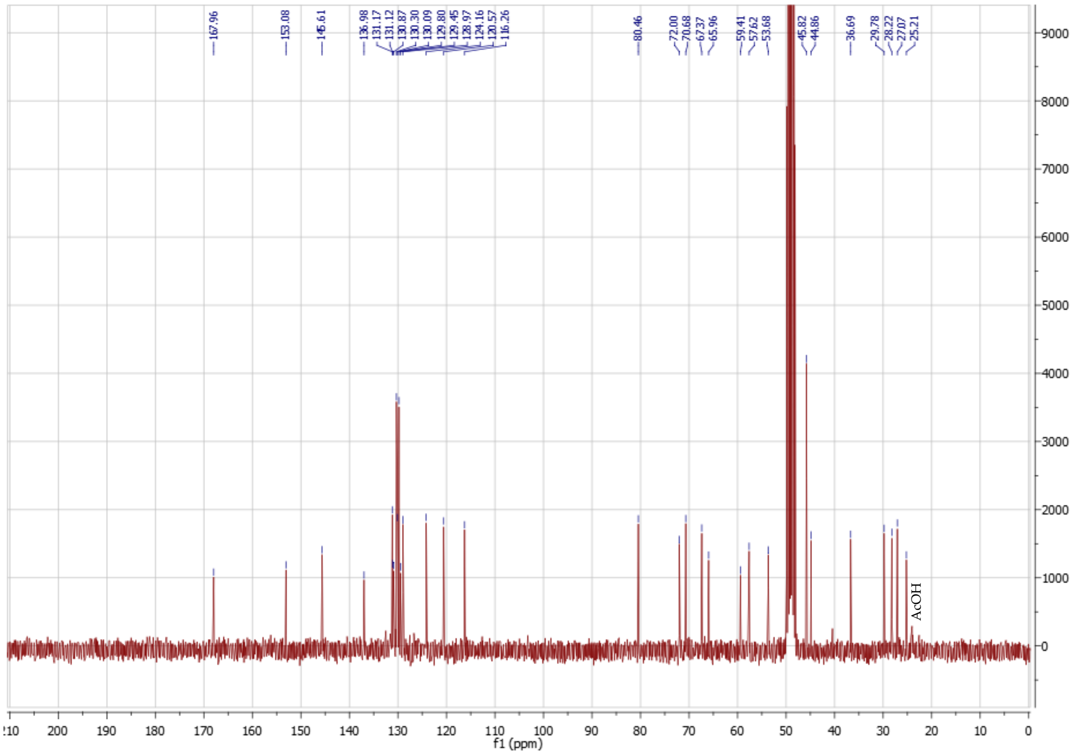

COSY (CD_3OD): Compound **55**

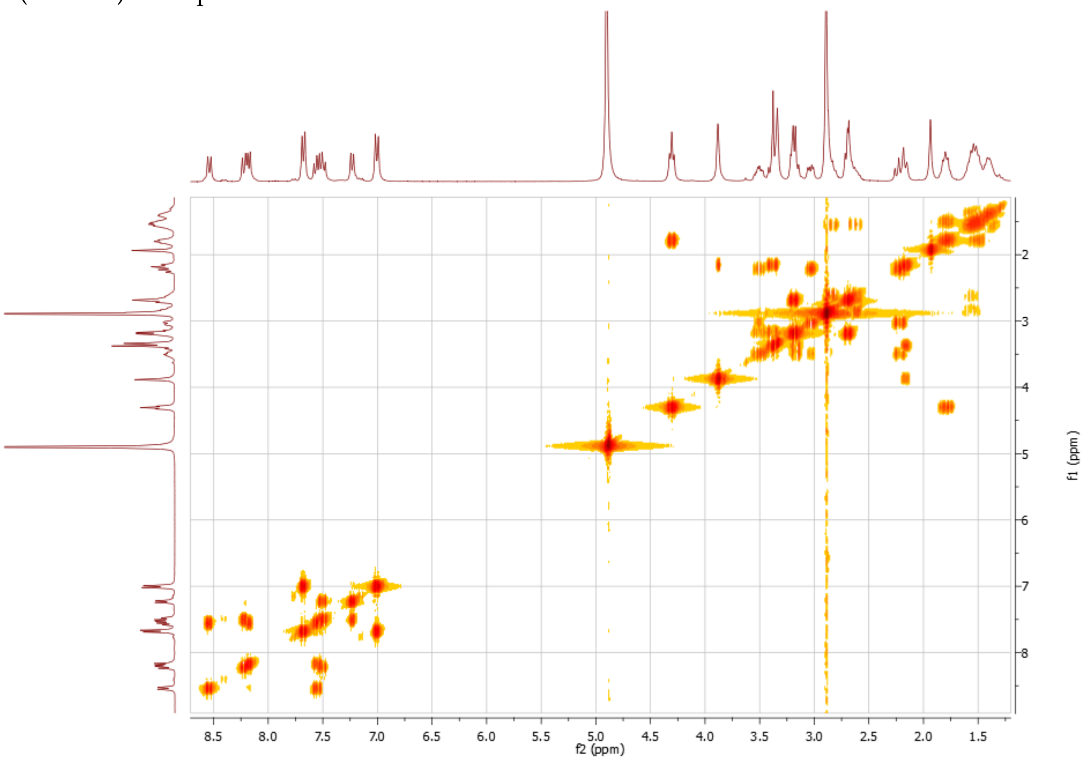

HSQC (CD_3OD): Compound **55**

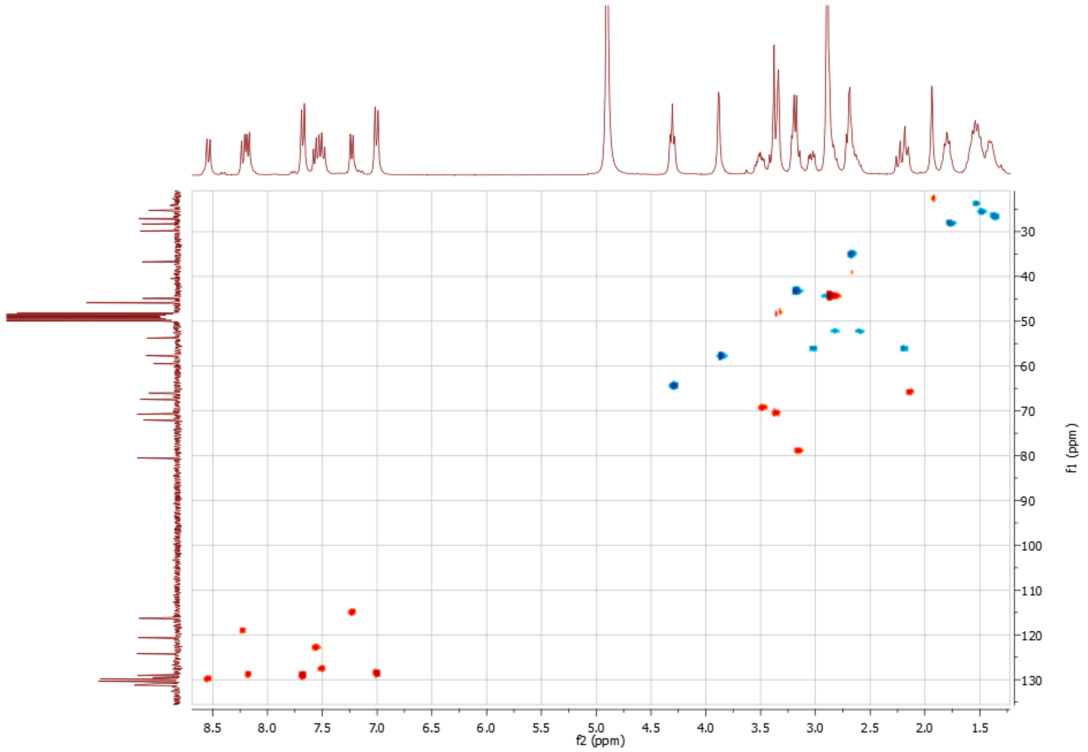

***N*-(6-((4-(2-(Dansylamino)ethyl)benzoyl)oxy)hexyl)-1,5-dideoxy-1,5-imino-D-xylitol (56)**

^{1}H-NMR (500 MHz, CD_3OD): Compound **56**

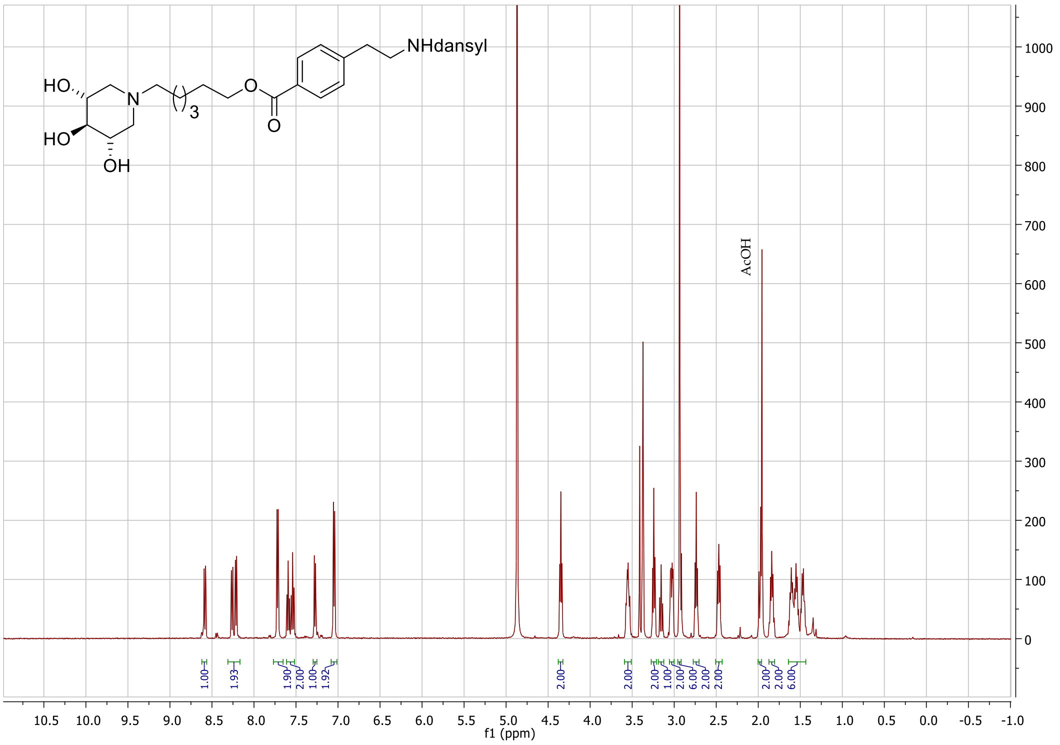

^{13}C-NMR (75.5 MHz, CD_3OD): Compound **56**

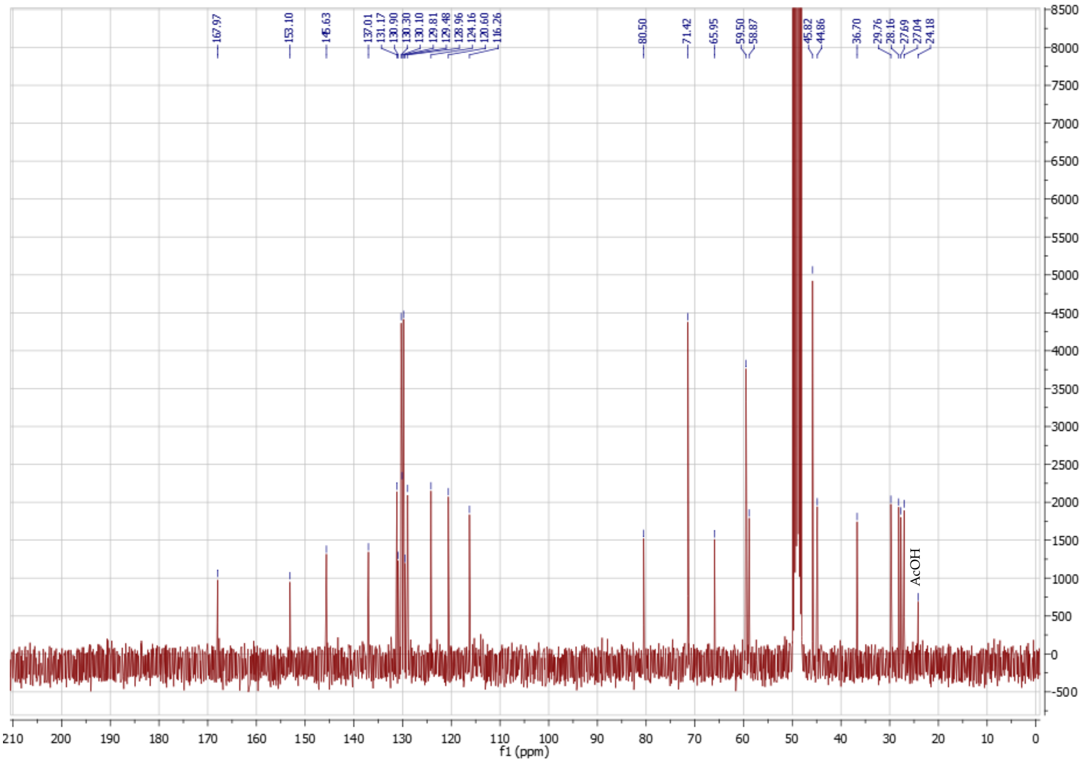

COSY (CD_3OD): Compound **56**

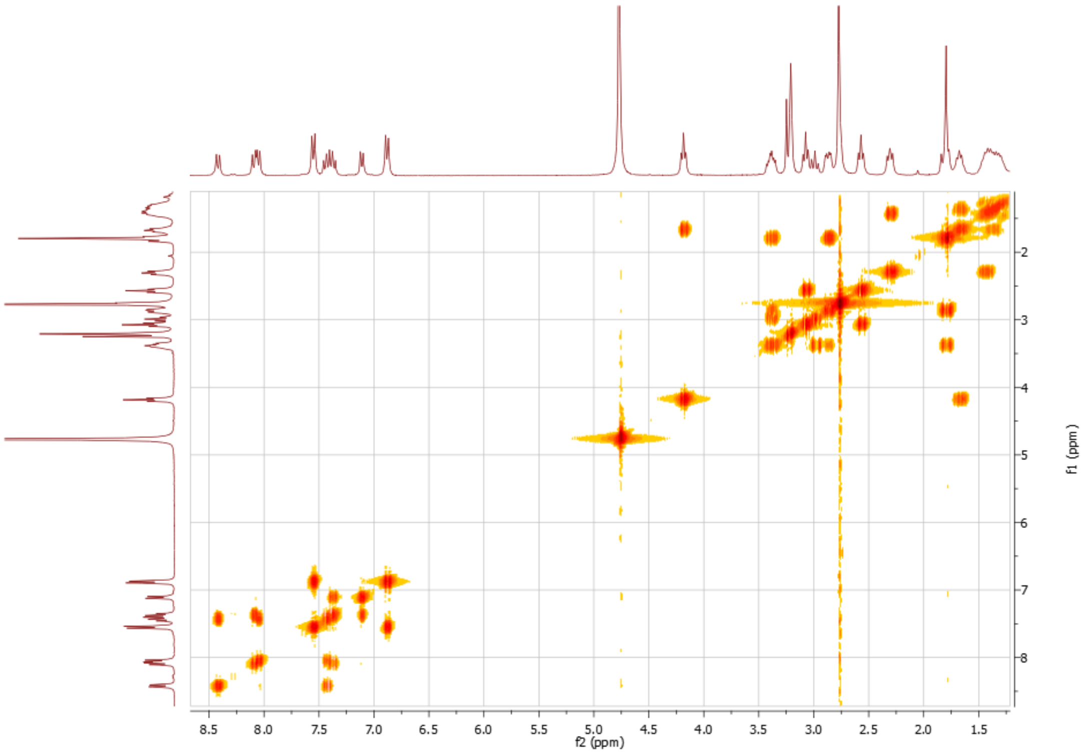

HSQC (CD_3OD): Compound **56**

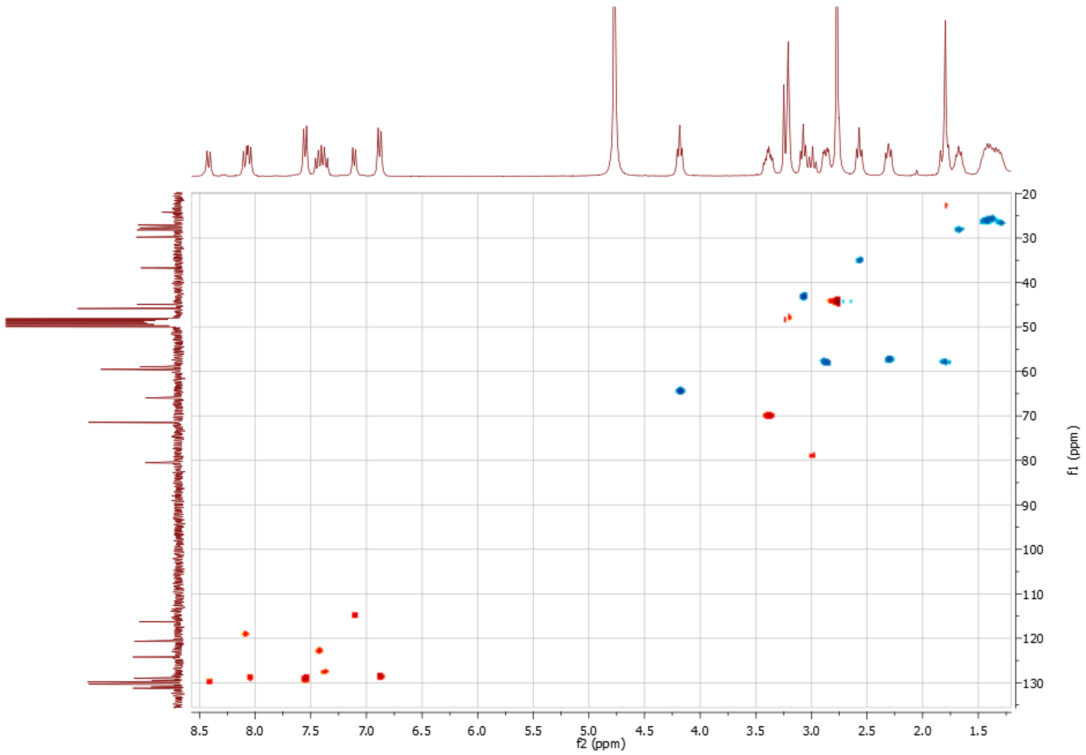

2) X-ray crystallography of compound 35:

For single crystal X-ray diffractometry suitable crystals were covered with a layer of silicone oil. Under a microscope a single crystal was selected, mounted on a glass rod on a copper pin, and placed in the cold N_2 stream provided by an Oxford Cryosystems cryometer (T=100 K). XRD data collection was performed on a Bruker APEX II [Bruker AXS Inc., Madison, Wisconsin, USA] diffractometer with use of Mo Kα radiation (λ = 0.71073 Å) from an IμS microsource and an APEX II CCD area detector. Data integration was carried out using SAINT [1]. Empirical absorption corrections were applied using SADABS [2,3].

The structure was solved by the dual space algorithm implemented in SHELXT [4]. Fourier analysis and refinement were performed by the full-matrix least-squares methods based on F^2 implemented in SHELXL [5] as implemented in SHELXLE [6].

The space group assignments and structural solutions were checked and evaluated using PLATON [7,8]. All non-hydrogen atoms were refined anisotropically. All hydrogen atoms were placed in calculated positions corresponding to standard bond lengths and angles using riding models. CIF files were edited, validated and formatted with the programs encipher [9] and OLEX2 [10].
Structural plots and figures were generated with MERCURY [11].

Details on refinement of compound **35**.

Crystal Data for **35**, $C_{16}H_{18}O_4$ (M = 274.30 g/mol): triclinic, space group P-1 (no. 2), a = 8.0910(5) Å, b = 8.9607(7) Å, c = 10.9606(7) Å, α = 76.641(4)°, β = 71.122(4)°, γ = 74.549(4)°, V = 715.54(9) $Å^3$, Z = 2, T = 99.97 K, μ(MoK$_\alpha$) = 0.091 mm^{-1}, *Dcalc* = 1.273 g/cm^3. A total of 16243 reflections were measured (3.978° ≤ 2Θ ≤ 57.998°), of which 3777 were unique (R_{int} = 0.0506, R_{sigma} = 0.0534) which were used in all calculations. Refinement proceeded normally without any constraints. The final R_1 was 0.0496 (I > 2σ(I)) and wR_2 was 0.1309 (all data).

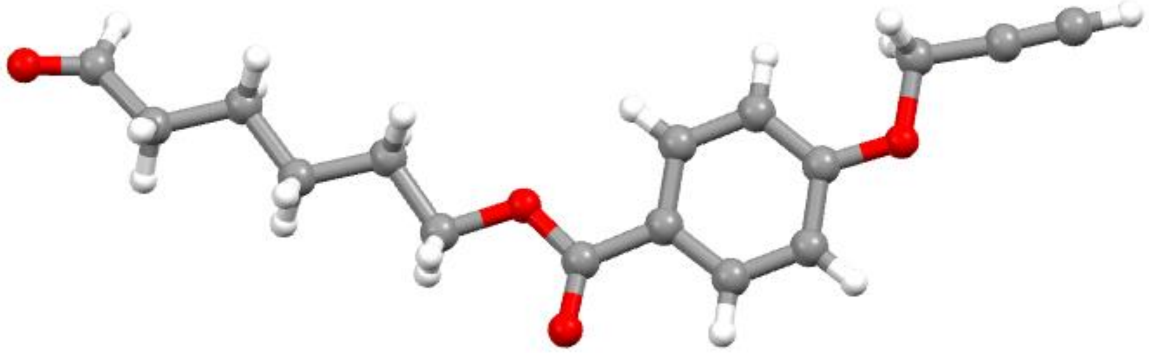

Figure S1. Structure of compound **35** confirmed by XRD analysis (CCDC 2021385).

Table 1: Crystal data and structure refinement for 35.

CCDC	2021385
Empirical formula	$C_{16}H_{18}O_4$
Formula weight	274.30
Temperature/K	99.97
Crystal system	triclinic
Space group	P-1
a/Å	8.0910(5)
b/Å	8.9607(7)
c/Å	10.9606(7)
α/°	76.641(4)
β/°	71.122(4)
γ/°	74.549(4)
Volume/Å^3	715.54(9)
Z	2
ρ_{calc}g/cm^3	1.273
μ/mm^{-1}	0.091
F(000)	292.0
Crystal size/mm^3	0.26 × 0.17 × 0.1
Radiation	MoKα (λ = 0.71073)
2Θ range for data collection/°	3.978 to 57.998
Index ranges	-11 ≤ h ≤ 11, -12 ≤ k ≤ 12, -14 ≤ l ≤ 14
Reflections collected	16243
Independent reflections	3777 [R_{int} = 0.0506, R_{sigma} = 0.0534]
Data/restraints/parameters	3777/0/181
Goodness-of-fit on F^2	1.041
Final R indexes [I>=2σ (I)]	R_1 = 0.0496, wR_2 = 0.1133
Final R indexes [all data]	R_1 = 0.0779, wR_2 = 0.1309
Largest diff. peak/hole / e Å^{-3}	0.35/-0.24

3) References:

[1] Bruker, *SAINT*, Bruker AXS Inc., Madison, Wisconsin, USA, 2012.

[2] Blessing, R.H. An Empirical Correction for Absorption Anisotropy. *Acta Crystallogr. Sect. A* **1995**, *A51*, 33–38, doi:10.1107/S0108767394005726.

[3] Sheldrick, G.M. *SADABS Version 2.10 Siemens Area Detector Correction.*; Universität Göttingen, Göttingen, Germany. 2003.

[4] Sheldrick, G.M. SHELXT - Integrated space-group and crystal-structure determination. *Acta Crystallogr. Sect. A Found. Crystallogr.* **2015**, *71*, 3–8, doi:10.1107/S2053273314026370.

[5] Sheldrick, G.M. Crystal structure refinement with SHELXL. *Acta Crystallogr. Sect. C Struct. Chem.* **2015**, *71*, 3–8, doi:10.1107/S2053229614024218.

[6] Hübschle, C.B.; Sheldrick, G.M.; Dittrich, B. SchelXle: a Qt graphical user interface for SHELXL. *J. Appl. Cryst.* **2011**, *44*, 1281–1284, doi:10.1107/S0021889811043202.

[7] Spek, A.L. Structure validation in chemical crystallography. *Acta Crystallogr. Sect. D Biol. Crystallogr.* **2009**, *65*, 148–155, doi:10.1107/S090744490804362X.

[8] Spek, A.L. Single-crystal structure validation with the program PLATON. *J. Appl. Crystallogr.* **2003**, *36*, 7–13, doi:10.1107/S0021889802022112.

[9] Allen, F.H.; Johnson, O.; Shields, G.P.; Smith, B.R.; Towler, M. CIF applications. XV. enCIFer: a program for viewing, editing and visualizing CIFs. *J. Appl. Crystallogr.* **2004**, *37*, 335–338,

doi:10.1107/S0021889804003528.

[10] Dolomanov, O.V.; Bourhis, L.J.; Gildea, R.J.; Howard, J.A.K.; Puschmann, H. OLEX2: A complete structure solution, refinement and analysis program. *J. Appl. Crystallogr.* **2009**, *42*, 339–341, doi:10.1107/S0021889808042726.

[11] MacRae, C.F.; Sovago, I.; Cottrell, S.J.; Galek, P.T.A.; McCabe, P.; Pidcock, E.; Platings, M.; Shields G.P.; Stevens, J.S.; Towler, M.; Wood, P.A. Mercury 4.0: from visualization to analysis, design and prediction. *J. Appl. Crystallogr.* **2020**, *53*, 226–235, doi:10.1107/S1600576719014092.
